# Effectiveness and safety of East Asian herbal medicine for menopausal insomnia: a systematic review and meta-analysis

**DOI:** 10.3389/fphar.2024.1414700

**Published:** 2024-08-08

**Authors:** Chan-Young Kwon, Boram Lee, Ji-Yeon Lee

**Affiliations:** ^1^ Department of Oriental Neuropsychiatry, Dong-eui University College of Korean Medicine, Busan, Republic of Korea; ^2^ KM Science Research Division, Korea Institute of Oriental Medicine, Daejeon, Republic of Korea; ^3^ Department of Obstetrics and Gynecology, College of Korean Medicine, Daejeon University, Daejeon, Republic of Korea

**Keywords:** insomnia, sleep Initiation and maintenance disorders, climacteric, herbal medicine, East Asian traditional medicine

## Abstract

**Background:** Menopausal insomnia significantly impacts the quality of life in women. East Asian herbal medicines (EAHMs) have been traditionally used in Asian countries, but their efficacy and safety require systematic evaluation. This systematic review and meta-analysis aimed to evaluate the effectiveness and safety of EAHM for treating menopausal insomnia.

**Methods:** A comprehensive literature search was conducted across 10 electronic databases from inception until 19 July 2023. Randomized controlled trials (RCTs) investigating EAHM for menopausal insomnia were included. Two reviewers independently screened studies, extracted data, and assessed the risk of bias using the Cochrane risk of bias tool. The primary outcome was sleep quality, insomnia severity, and sleep architecture. Secondary outcomes included total effective rate (TER), menopausal symptoms, and adverse effects. Meta-analysis was conducted using a random-effects model, and the results were calculated as mean differences (MDs) or risk ratios (RRs) and their 95% confidence intervals (CIs). Also, the certainty of evidence was assessed using the GRADE approach.

**Results:** A total of 70 RCTs involving 6,035 participants met the inclusion criteria. The most frequently used EAHMs were modified Suan Zao Ren Tang, and the most frequently used herbal component was Zizyphus jujuba Miller var. spinosa Hu ex H. F. Chou [Rhamnaceae; Zizyphi Semen]. Compared with sedative-hypnotics, EAHM significantly improved sleep quality, as measured by the Pittsburgh Sleep Quality Index (PSQI) (MD –2.18, 95% CI –2.56 to −1.80), and reduced menopausal symptoms, as assessed by the Kupperman Index (MD –4.92, 95% CI –6.03 to −3.80). Similar results were seen when EAHM was added to sedative-hypnotics. When EAHM was additionally used in sedative-hypnotics, similar benefits were shown for PSQI (MD –2.46, 95% CI –3.09 to −1.82) and the Kupperman Index (MD –4.64, 95% CI –5.07 to −4.21). EAHM was generally safer than sedative-hypnotics, with significantly fewer adverse reactions (RR 0.15, 95% CI 0.07–0.34). However, the certainty of evidence was moderate to low.

**Conclusion:** EAHMs, alone or with sedative-hypnotics, may be effective and safe for improving sleep quality and managing menopausal symptoms. Future studies should include diverse populations, rigorous methodologies, and explore mechanisms of action to confirm these findings.

Systematic Review Registration: [https://www.crd.york.ac.uk/prospero/display_record.php?], identifier [CRD42023446708].

## 1 Introduction

Rapid change in sex hormones that begins during the menopausal transition can affect many biological systems, causing clinical signs and symptoms in various areas of the body, including the central nervous system, endocrine system, genitourinary system, musculoskeletal system, and cardiovascular system (Monteleone et al., 2018). Changes in sleep patterns are also common during menopause, and according to a study including the Study of Women Across the Nation cohort, 30.8% of menopausal women suffer from one or more sleep disorders (Kravitz and Joffe, 2011). A recent meta-analysis showed that the prevalence of sleep disorders during menopause is 51.6% (Salari et al., 2023). The presence of this sleep disorder is clinically important because it not only seriously impairs quality of life in itself but is also associated with negative health conditions such as cardiometabolic outcomes in this population (Ciano et al., 2017).

Treatment for menopausal insomnia includes cognitive-behavioral treatment of insomnia, hormone therapy, non-hormonal pharmacological medications, and non-pharmacological and self-management strategies (Baker et al., 2018). Meanwhile, complementary and integrative medicine (CIM) is considered a popular and evidence-based menopausal symptom management method (Ebrahimi et al., 2020), with approximately one in two women using CIM specifically for menopausal symptoms (Posadzki et al., 2013). South Korea is a country where Korean medicine (KM), which is a form of traditional East Asian medicine (TEAM), plays a major role in the national medical system (Kim et al., 2021), and KM doctors utilize some CIM modalities including East Asian herbal medicine (EAHM) to treat menopausal disorders (Jun et al., 2019). In TEAM theory and practice, individualized treatment is emphasized with EAHM, using mixtures of whole plants or specific parts of herbs in a holistic approach (Marshall, 2020; Li et al., 2021). This contrasts with Western practices, where dietary supplements are typically developed using one or two medicinal herbs (Li et al., 2021).

Recent studies have shown that EAHM can result in significant clinical benefits by improving menopausal symptoms (Li et al., 2019) as well as insomnia (Ni et al., 2015). The sedative-hypnotic effects of some EAHMs that have been used for insomnia, *Zizyphus jujuba Miller* var. *spinosa Hu ex H. F. Chou* [Rhamnaceae; Zizyphi Semen], *Glycyrrhiza glabra L.* [Fabaceae; Glycyrrhizae Radix et Rhizoma], and *Paeonia lactiflora Pall.* [Paeoniaceae; Paeoniae Radix], have been demonstrated (Shi et al., 2016). Several alkaloids, terpenoids, and volatile oils, flavonoids, lignanoids and coumarins, and saponins have been found to be responsible for the sedative-hypnotic effects of these EAHMs in experimental studies (Shi et al., 2016). For example, EAHMs based on *Zizyphus jujuba M.* var. *spinosa Hu ex H. F. Chou* [Rhamnaceae; Zizyphi Semen] have been demonstrated to have clinical benefit in the treatment of insomnia as a monotherapy or in combination with sedative-hypnotics, and its therapeutic mechanism has been found to mainly mediate the GABAergic and serotonergic systems (Zhou et al., 2018). Therapeutic mechanisms of EAHM are often described as multiple components-multiple targets-multiple pathways (Zhang et al., 2019), suggesting the multiple effectiveness of this therapeutic intervention.

However, the clinical effects of EAHM on menopausal insomnia and/or concurrent menopausal symptoms have not been sufficiently studied. Although an earlier systematic review have investigated the effects of EAHM on sleep dysfunction in peri- and post-menopause (Khadivzadeh et al., 2018), TEAM-based herbal medicine (i.e., EAHM) was rarely considered in this study. The databases searched in the existing study were MEDLINE, Scopus and the Cochrane Library (Khadivzadeh et al., 2018), which lacked databases related to EATM, and the search strategy used was not specifically designed for TEAM modalities. For example, a meta-epidemiological study on acupuncture studies, another representative TEAM modality, found the use of intervention-specific databases such as China National Knowledge Infrastructure (CNKI) and WanFang, as well as PubMed and the Cochrane Library, enabled efficient literature search (Guo et al., 2022). Also, as TEAM has a significant impact on population health in Asian countries such as South Korea, China, Japan, and Taiwan (Park et al., 2012), this issue is clinically relevant and needs urgent attention. Systematic reviews are considered an appropriate research methodology to enable the integration of TEAM-based therapeutic interventions, such as EAHM, into evidence-based clinical practice (Zhang et al., 2011).

Therefore, the purpose of this study is to systematically review the effectiveness and safety of oral EAHM, based on TEAM, as monotherapy or adjunctive therapy (i.e., intervention) in improving sleep outcomes (i.e., outcomes) among climacteric women with insomnia (i.e., population), compared with wait-list, placebo, or active controls (i.e., comparator). This review focused on EAHM based on TEAM, which was insufficiently covered in a previous systematic review (Khadivzadeh et al., 2018).

## 2 Methods

This systematic review adhered to the Preferred Reporting Items for Systematic reviews and Meta-Analyses statement (Page et al., 2021).

### 2.1 Protocol registration

The protocol of the systematic review was registered in PROSPERO (CRD42023446708), and there were no deviations from the protocol.

### 2.2 Eligibility criteria

The inclusion criteria for this review are summarized in the following P-I-C-O-S format.

#### 2.2.1 Population

Climacteric (perimenopausal, menopausal, or postmenopausal) women diagnosed with insomnia or complaining of insomnia symptoms were included, without limitation on age, race, and nationality. Patients with insomnia having severe mental disorders such as schizophrenia were excluded from the study.

#### 2.2.2 Intervention

Studies involving oral EAHMs as monotherapy or adjunctive therapies to conventional medication such as sedative-hypnotics, with or without routine care for menopausal conditions were included. Any dosage form of oral EAHMs prescribed based on TEAM theories was allowed.

#### 2.2.3 Comparator

Studies involving wait-list, placebo EAHM, or conventional medication such as sedative-hypnotics, with or without routine care for menopausal conditions, as a control group interventions were included. However, studies that compared the effects of different EAHMs or studies that used TEAM-based interventions such as acupuncture as a control group intervention were excluded.

#### 2.2.4 Primary outcome

Sleep-specific outcomes: These include measures of sleep quality, insomnia severity, and sleep architecture assessed after treatment. These outcomes are evaluated using both subjective measures [such as the Pittsburgh Sleep Quality Index (PSQI) (Buysse et al., 1989) and the Insomnia Severity Index (ISI) (Bastien et al., 2001)] and objective measures (such as polysomnography and actigraphy data). The PSQI evaluates overall sleep quality and disturbances, while the ISI assesses the severity of insomnia symptoms.

#### 2.2.5 Secondary outcome

(1) Total effective rate (TER): This is calculated based on improvements in insomnia symptoms post-treatment; (2) menopausal symptoms index scores: These scores assess the severity and frequency of menopausal symptoms; (3) incidence of adverse events: This measures the occurrence of any adverse events reported during or after treatment.

#### 2.2.6 Study design

Only randomized controlled clinical trials (RCTs) with appropriate randomization methods were included. In other words, referring to existing studies that cautioned against the lack of appropriate description of randomization in some RCTs (Wu et al., 2009), this review included studies that described specific randomization methods, such as random number table generation.

#### 2.2.7 Others

There were no restrictions on publication language. In addition to articles published in journals, conference proceedings were also included.

### 2.3 Search sources and strategy

The following 10 electronic databases were searched on 19 July 2023: Medline via PubMed, Embase via Elsevier, Cochrane Central Register of Controlled Trials via Cochrane Library, Allied and Complementary Medicine Database via EBSCO, Oriental Medicine Advanced Searching Integrated System, Research Information Sharing Service, Korean Medical Database, ScienceON, CNKI, and Wanfang data. Search terms were set under discussion between a specialist in obstetrics and gynecology of KM and systematic review experts. The references cited in the relevant studies were searched to find additional eligible studies. The full search strategies and search results are described in Supplementary Material S1.

### 2.4 Study selection and data collection

Using EndNote 20 (Clarivate Analytics, PA, United States), the titles and abstracts of studies searched from the databases and those identified from additional sources were screened. The full texts of the eligible studies were retrieved and assessed for final inclusion. A pilot-tested Excel form was used to extract data from the included studies. The extracted information included study characteristics (name of the first author, year of publication, country, ethical approval, and sample size), details of participants, interventions, comparators, outcomes of interest, results, and information for assessment of the risk of bias. Two researchers (CYK and BL) conducted the study selection independently. One researcher (CYK) conducted data extraction, and another researcher (BL) reviewed the results. All discrepancies were resolved through discussion with the corresponding author (JYL).

### 2.5 Risk of bias assessment

The Cochrane risk of bias tool was used to assess the risk of bias in the included studies (Higgins et al., 2011). The following seven domains were assessed and categorized as low, unclear, and high risk of bias for each included study: random sequence generation, treatment allocation concealment, blinding of participants and personnel, blinding of outcome assessment, completeness of outcome data, selective outcome reporting, and other sources of bias. Other sources of bias were assessed, especially focusing on the statistical homogeneity of baseline characteristics between the treatment and control groups. One researcher (CYK) conducted a risk of bias assessment, and another researcher (BL) reviewed the results. Any discrepancies were resolved through discussion with the corresponding author (JYL).

### 2.6 Data analysis and synthesis

Descriptive analysis was performed on all included studies. Where two or more studies compared the same type of interventions and comparators, with the same outcome measures, meta-analysis was conducted using Review Manager software, version 5.4 (Cochrane, London, UK). Continuous variables were synthesized using mean difference (MD), and dichotomous variables were synthesized using risk ratio (RR), with 95% confidence intervals (CIs). Statistical heterogeneity between the studies was assessed using the I^2^ statistic, and an I^2^ value greater than 50% was considered substantial heterogeneity. The results were pooled using a random-effects model considering the inevitable clinical heterogeneity of EAHMs used in each study. A subgroup analysis was planned according to the EAHM administration period [≤1 mo, >1 mo ≤ 2 mo, and >2 mo (Kwon et al., 2019)] and baseline insomnia severity of participants. When 10 or more studies were included in the meta-analysis, the evidence for publication bias was tested through the asymmetry of the funnel plot and Egger’s test.

### 2.7 Certainty assessment

The certainty of evidence of effect estimates was assessed using the Grading of Recommendations, Assessment, Development, and Evaluations (GRADE) approach (Balshem et al., 2011). The risk of bias, inconsistency, indirectness, imprecision, and publication bias were assessed for each estimate, and the certainty of evidence was presented as High, Moderate, Low, and Very Low.

## 3 Results

### 3.1 Study selection and characteristics

**FIGURE 1:**
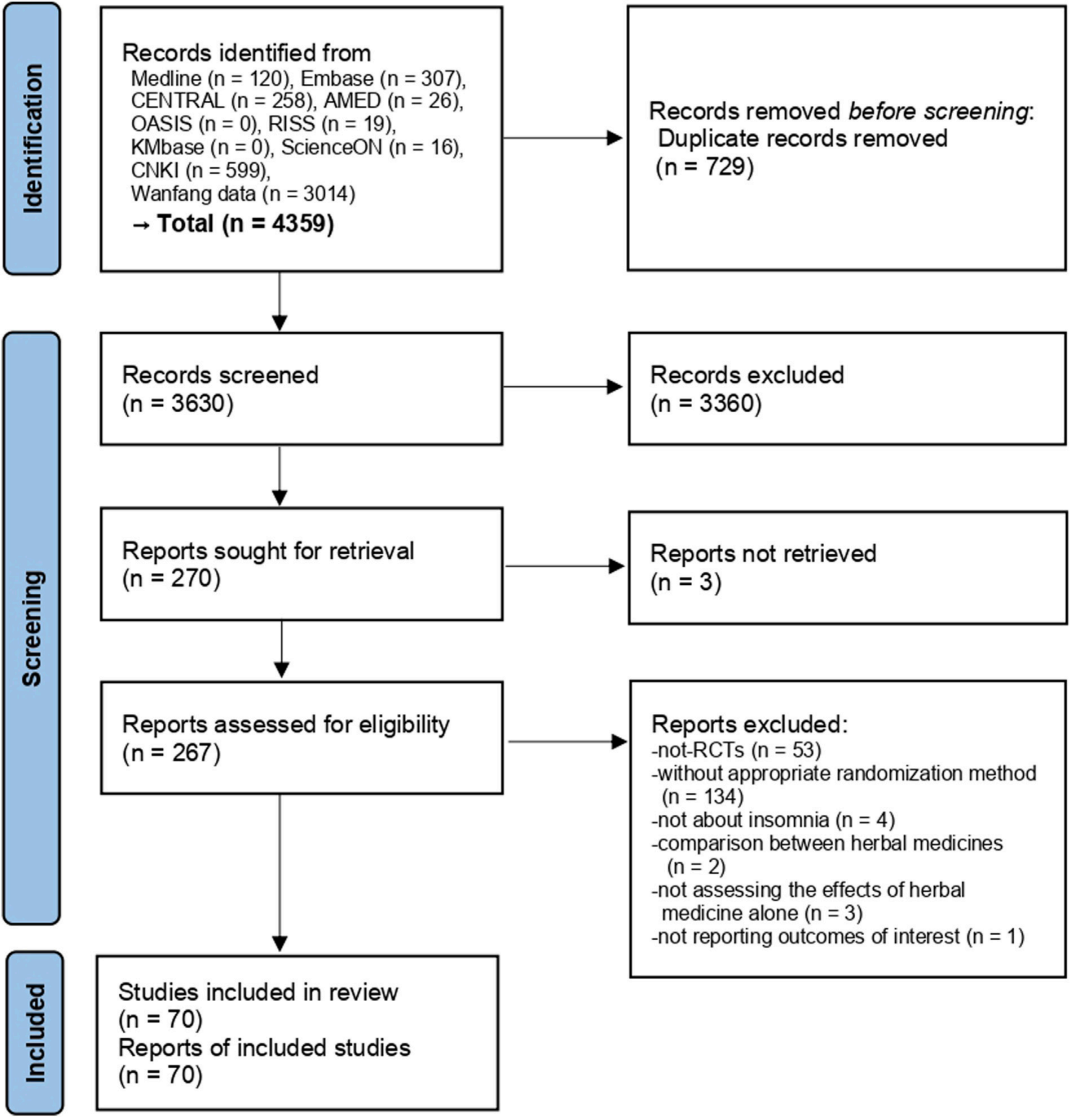
Study selection process. RCT, randomized controlled clinical trial.

A total of 4,359 studies were searched from various information sources and after the removal of duplicates, the titles and abstracts of 3,630 studies were screened. In total, 267 studies met our selection criteria, and the full texts of these studies were retrieved and assessed. After excluding 53 not-RCTs, 134 studies without appropriate randomization method, four studies not targeting insomnia, two studies comparing EAHMs, three studies where the effects of EAHM alone were not considered, and one study not reporting outcomes of interest (Supplementary Material S2), 70 studies involving 6,035 participants (Chen and Du, 2007; Lu, 2007; Jia and Yang, 2008; Zhang and Chen, 2009; Lai et al., 2011; Chen and Yang, 2012; Zhang et al., 2012; Lei, 2013; Bai et al., 2014; Geng and Yu, 2014; Jia, 2014; Wang, 2014; Yao et al., 2014; Li and Luo, 2015; Ye et al., 2015; Guo, 2016; Li, 2016; Sun and Huang, 2016; Weng, 2016; Zheng, 2016; Hua, 2017; Rui et al., 2017; Zhao et al., 2017; Wang F. et al., 2018; Wang L. et al., 2018; Li and Gu, 2018; Ma, 2018; Shen and Wang, 2018; Shi, 2018; Xing, 2018; Zhao, 2018; Cai et al., 2019; Chen et al., 2019; Hu, 2019; Lu, 2019; Pang, 2019; Pu and Wang, 2019; Li X., 2020; Li Z., 2020; Hu, 2020; Huang et al., 2020; Lan, 2020; Mahmoudi et al., 2020; Mao, 2020; Shan and Du, 2020; Sun and Tian, 2020; Wei and Yang, 2020; Zhang et al., 2020; Zheng, 2020; Zhu and Wang, 2020; Wang M.-R. et al., 2021; Wang Q. et al., 2021; Li, 2021; Liu, 2021; Luo et al., 2021; Qiao et al., 2021; Su et al., 2021; Zhang et al., 2021; Lai et al., 2022; Li and Zhang, 2022; Mi et al., 2022; Pan et al., 2022; Wang et al., 2022; Wang and Wang, 2022; You et al., 2022; Zeng et al., 2022; Jia et al., 2023; Qin, 2023; Wang and Ma, 2023; Xu et al., 2023) were finally included (Figure 1).

One study was conducted in Iran (Mahmoudi et al., 2020), and the remaining sixty-nine studies were conducted in China. In addition to menopausal insomnia, three studies (Wang L. et al., 2018; Wei and Yang, 2020; Li and Zhang, 2022) targeted patients with accompanied anxiety, one study (Luo et al., 2021) targeted patients with accompanied depression, and three studies (Yao et al., 2014; Li and Gu, 2018; Qiao et al., 2021) targeted patients with accompanied hypertension. The remaining studies only targeted patients with menopausal insomnia. There were 27 studies that targeted participants with specific pattern identification, of which 10 were related to the liver and kidney (Zhang et al., 2012; Lei, 2013; Wang F. et al., 2018; Pu and Wang, 2019; Wei and Yang, 2020; Zhang et al., 2020; Lai et al., 2022; Mi et al., 2022; Wang and Ma, 2023; Xu et al., 2023), two were liver- and spleen-related (Chen et al., 2019; Qin, 2023), two were liver-related (Wang M.-R. et al., 2021; Pan et al., 2022), six were heart- and kidney-related (Zhang and Chen, 2009; Li and Luo, 2015; Mao, 2020; Qiao et al., 2021; Zeng et al., 2022; Jia et al., 2023), three were about yin deficiency and fire excess (Geng and Yu, 2014; Cai et al., 2019; Lu, 2019), two were kidney-related (Li and Gu, 2018; Su et al., 2021), one was about deficiency of blood and yin (Weng, 2016), and one about was phlegm-heat (Sun and Huang, 2016). Twenty studies (Rui et al., 2017; Shen and Wang, 2018; Shi, 2018; Xing, 2018; Cai et al., 2019; Chen et al., 2019; Li X., 2020; Huang et al., 2020; Mahmoudi et al., 2020; Sun and Tian, 2020; Wei and Yang, 2020; Zhu and Wang, 2020; Wang M.-R. et al., 2021; Li, 2021; Qiao et al., 2021; Li and Zhang, 2022; Mi et al., 2022; Pan et al., 2022; Zeng et al., 2022; Jia et al., 2023) reported receiving ethical approval from an institutional review board, and the remaining studies had no relevant mentions about approval.

Overall, 39 studies compared EAHM and conventional medication, of which 36 used sedative-hypnotics (Chen and Du, 2007; Zhang and Chen, 2009; Lai et al., 2011; Zhang et al., 2012; Lei, 2013; Bai et al., 2014; Geng and Yu, 2014; Wang, 2014; Li and Luo, 2015; Ye et al., 2015; Guo, 2016; Li, 2016; Sun and Huang, 2016; Weng, 2016; Hua, 2017; Zhao et al., 2017; Wang F. et al., 2018; Li and Gu, 2018; Xing, 2018; Zhao, 2018; Chen et al., 2019; Hu, 2019; Lu, 2019; Li Z., 2020; Lan, 2020; Sun and Tian, 2020; Wei and Yang, 2020; Zheng, 2020; Zhu and Wang, 2020; Wang Q. et al., 2021; Liu, 2021; Zhang et al., 2021; Li and Zhang, 2022; Wang et al., 2022; Jia et al., 2023; Xu et al., 2023), one used estradiol (Wang and Ma, 2023), and two used oryzanol (Lu, 2007; Li, 2021) as the control group. In 28 studies, the authors evaluated the effectiveness of EAHM as an add-on therapy to conventional medication, of which 23 studies used sedative-hypnotics (Chen and Yang, 2012; Jia, 2014; Zheng, 2016; Rui et al., 2017; Ma, 2018; Shen and Wang, 2018; Shi, 2018; Cai et al., 2019; Pang, 2019; Pu and Wang, 2019; Hu, 2020; Huang et al., 2020; Mao, 2020; Shan and Du, 2020; Zhang et al., 2020; Wang M.-R. et al., 2021; Qiao et al., 2021; Su et al., 2021; Lai et al., 2022; Pan et al., 2022; Wang and Wang, 2022; You et al., 2022; Qin, 2023), three studies used antidepressants (Jia and Yang, 2008; Wang L. et al., 2018; Luo et al., 2021), one study used estradiol (Li X., 2020), and one study used losartan potassium (Yao et al., 2014) as the control group. In three studies (Mahmoudi et al., 2020; Mi et al., 2022; Zeng et al., 2022), EAHM was compared with placebo EAHM (Supplementary Material S3).

### 3.2 Characteristics of EAHM used

A total of 71 kinds of EAHMs were used, including one study that used 2 EAHMs in succession (Chen et al., 2019). Depending on the study, various EAHMs were administered to participants for various treatment periods, and the most frequently used EAHMs were modified Suan Zao Ren Tang (Wang, 2014; Li, 2016; Zhao et al., 2017; Zhao, 2018; Sun and Tian, 2020; Wang M.-R. et al., 2021), followed by modified Gan Mai Da Zao Tang (Lu, 2007; Wang F. et al., 2018; Hu, 2019; Zheng, 2020; Li, 2021), and modified Chaihu Jia Longgu Muli Tang (Chen et al., 2019; Wang Q. et al., 2021; Pan et al., 2022; Wang and Wang, 2022). In 35 studies, specific botanical drugs were added or removed based on patients’ specific symptoms or pattern identification (Lu, 2007; Jia and Yang, 2008; Zhang and Chen, 2009; Chen and Yang, 2012; Bai et al., 2014; Geng and Yu, 2014; Wang, 2014; Sun and Huang, 2016; Zheng, 2016; Rui et al., 2017; Zhao et al., 2017; Ma, 2018; Shen and Wang, 2018; Shi, 2018; Xing, 2018; Zhao, 2018; Cai et al., 2019; Chen et al., 2019; Hu, 2019; Lu, 2019; Li Z., 2020; Hu, 2020; Shan and Du, 2020; Sun and Tian, 2020; Zheng, 2020; Zhu and Wang, 2020; Wang M.-R. et al., 2021; Wang Q. et al., 2021; Li, 2021; Qiao et al., 2021; Zhang et al., 2021; Lai et al., 2022; Pan et al., 2022; Wang et al., 2022; Qin, 2023). As for the dosage form of EAHM, capsules were used in three (Li X., 2020; Mahmoudi et al., 2020; Li and Zhang, 2022), granules in (Zeng et al., 2022; Jia et al., 2023), mixtures in two (Li and Luo, 2015; Liu, 2021), and pills in three studies (Li and Gu, 2018; Pang, 2019; Luo et al., 2021); various decoction methods were used in the remaining studies. Only 11 studies (Li and Gu, 2018; Li X., 2020; Mahmoudi et al., 2020; Zhang et al., 2020; Wang M.-R. et al., 2021; Luo et al., 2021; Qiao et al., 2021; Li and Zhang, 2022; Mi et al., 2022; You et al., 2022; Jia et al., 2023) reported the pharmaceutical producer of EAHM used. Among the included studies, none reported quality control measures or chemical analysis of EAHM. Among the EAHMs used, three were approved by relevant health authorities: Qiju Dihuang Pill (Chinese medicine no. Z41021905) (Li and Gu, 2018), Kuntai Capsule (Z20000083) (Li X., 2020; Li and Zhang, 2022), and Danzhi Xiaoyao Pill (Z53020866) (Luo et al., 2021). The treatment period in the included studies varied from 7 days to 3 months. In one study, the treatment period was not specified (Hua, 2017), in another study, the specified treatment period was 4–8 weeks (Jia and Yang, 2008). In the remaining 68 studies, the average treatment period was 35.18 days (Supplementary Material S4).

The most frequently used botanical drugs in the included studies were *Zizyphus jujuba M.* var. *spinosa Hu ex H. F. Chou* [Rhamnaceae; Zizyphi Semen] (42 studies), followed by *G. glabra L.* [Fabaceae; Glycyrrhizae Radix et Rhizoma] (39 studies), *P. lactiflora Pall.* [Paeoniaceae; Paeoniae Radix] (32 studies), *Poria cocos Wolf* [Polyporaceae; Poria Sclerotium] (29 studies), *Bupleurum falcatum L.* [Apiaceae; Bupleuri Radix] (28 studies), *Angelica gigas Nakai* [Apiaceae; Angelicae Gigantis Radix] (24 studies), and *Ostrea gigas Thunberg* [Ostreidae; Ostreae Testa] (24 studies) (Supplementary Material S5).

### 3.3 Risk of bias assessment

**FIGURE 2:**
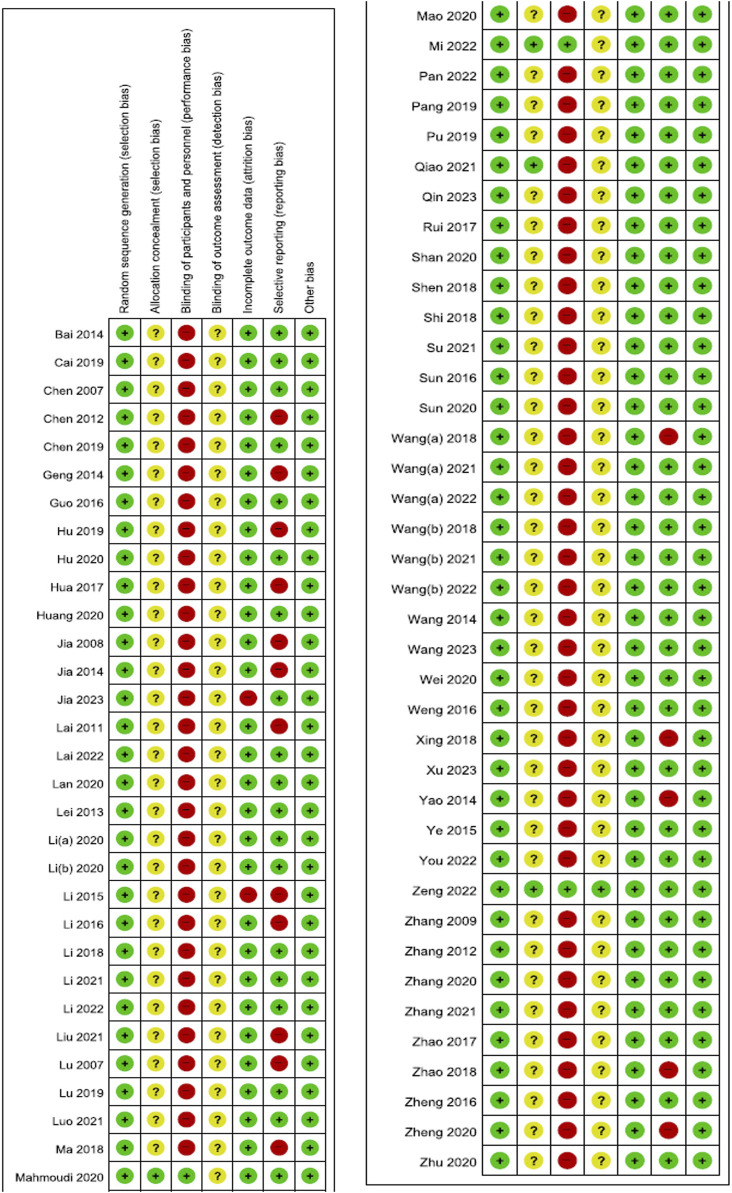
Risk of bias summary Low, unclear, and high risk, respectively, are represented with the following symbols: “+”, “?”, and “-”.

All studies were deemed to generate random sequences appropriately by using random number tables. In four studies (Mahmoudi et al., 2020; Qiao et al., 2021; Mi et al., 2022; Zeng et al., 2022), allocation concealment was conducted using envelopes, and only three studies (Mahmoudi et al., 2020; Mi et al., 2022; Zeng et al., 2022) where EAHM and placebo EAHM were compared conducted blinding of participants and personnel appropriately. Blinding of outcome assessor was reported only in one study (Zeng et al., 2022). Two studies (Li and Luo, 2015; Jia et al., 2023) included dropouts but no information on the reasons for their withdrawal was stated, and per-protocol analysis was performed, resulting in high risk of attrition bias. In seventeen studies (Lu, 2007; Jia and Yang, 2008; Lai et al., 2011; Chen and Yang, 2012; Geng and Yu, 2014; Jia, 2014; Yao et al., 2014; Li and Luo, 2015; Li, 2016; Hua, 2017; Wang L. et al., 2018; Ma, 2018; Xing, 2018; Zhao, 2018; Hu, 2019; Zheng, 2020; Liu, 2021), validated evaluation tools were not used and secondary indicators, such as TER, were evaluated as high risk of reporting bias (Figure 2; Supplementary Material S6).

### 3.4 Effectiveness and safety of herbal medicine as a monotherapy

1) *Versus conventional medications for insomnia.* Compared with sedative-hypnotics, EAHM significantly improved sleep quality measured using the PSQI [20 studies, mean difference (MD) −2.18, 95% confidence interval (CI) −2.56 to −1.80]. However, no significant difference was observed in the Self-rating Scale of Sleep (two studies, MD –0.97, 95% CI –3.26 to 1.32) and the Athens Insomnia Scale (two studies, MD –1.80, 95% CI –4.10 to 0.50) scores. In one study (Xu et al., 2023), sleep quality was measured using the Spiegel Sleep Questionnaire, and it was significantly improved with EAHM compared with sedative-hypnotics (p < 0.05). TER based on insomnia symptoms was significantly higher in the EAHM group [30 studies, RR 1.16, 95% CI 1.13 to 1.20] than in the sedative-hypnotics group. In addition, menopausal symptoms, measured using the Kupperman Index (five studies, MD –4.92, 95% CI –6.03 to −3.80) and Greene climacteric scale (Li and Luo, 2015) (p < 0.05), significantly improved in the EAHM group. Furthermore, the incidence of adverse events was significantly lower in the EAHM group (14 studies, RR 0.15, 95% CI 0.07–0.34) than in the sedative-hypnotics group (Table 1).
2) *Versus conventional medications for menopausal disorders.* Compared with estradiol, EAHM significantly improved PSQI scores (p < 0.01). However, estradiol significantly improved the Kupperman Index scores than EAHM (p < 0.01), and no difference in TER based on insomnia symptoms were observed (EAHM, 93.3%; estradiol, 90.0%) (Wang and Ma, 2023). Compared with oryzanol, EAHM significantly improved PSQI (Li, 2021) and Kupperman Index scores (Li, 2021), along with TER based on insomnia symptoms (Lu, 2007; Li, 2021) (p < 0.05, all), with no difference in incidence of adverse events (Li, 2021).
3) *Versus placebo.* Compared with placebo EAHM, EAHM significantly improved PSQI scores (two studies, MD –5.05, 95% CI –9.06 to −1.04) (Table 1). In addition, TER based on insomnia symptoms (Mi et al., 2022) and Kupperman Index scores (Mi et al., 2022; Zeng et al., 2022) were significantly improved (p < 0.05, all), without any adverse events (Mi et al., 2022; Zeng et al., 2022).
4) *Subgroup analysis.* Overall, subgroup analysis based on the EAHM administration period did not show significant changes in statistical heterogeneity and effect size (Table 1).

**TABLE 1:** Level of evidence for EAHM as monotherapy.

Outcomes	Subgroup	No. participants (RCTs)	Effect estimate [95% CI]		*I* ^*2*^ value (%)	Certainty of evidence	Reasons for downgrading
EAHM versus sedative-hypnotics							
PSQI	Total	1826 (20)	MD	**−2.18 [-2.56, -1.80]**	93	Moderate	Risk of bias
	≤1mo	1554 (17)	MD	**−2.05 [-2.44, -1.67]**	89	Moderate	Risk of bias
	>2mo	272 (3)	MD	**−2.78 [-3.87, -1.68]**	91	Low	Risk of bias, Imprecision
SRSS	Total (≤1mo)	160 (2)	MD	−0.97 [-3.26, 1.32]	68	Very low	Risk of bias, Inconsistency, Imprecision
AIS	Total	140 (2)	MD	−1.80 [-4.10, 0.50]	74	Low	Risk of bias, Imprecision
	≤1mo	60 (1)	MD	**−2.90 [-4.35, -1.45]**	NA	Low	Risk of bias, Imprecision
	>1mo ≤ 2mo	80 (1)	MD	−0.55 [-2.41, 1.31]	NA	Low	Risk of bias, Imprecision
TER (insomnia)	Total	2615 (30)	RR	**1.16 [1.13, 1.20]**	0	Low	Risk of bias, Publication bias
	≤1mo	2223 (25)	RR	**1.16 [1.12, 1.20]**	4	Low	Risk of bias, Publication bias
	>1mo ≤ 2mo	200 (2)	RR	**1.25 [1.07, 1.45]**	0	Moderate	Risk of bias
	>2mo	152 (2)	RR	**1.15 [1.01, 1.31]**	0	Moderate	Risk of bias
	Not reported	40 (1)	RR	1.19 [0.93, 1.51]	NA	Low	Risk of bias, Imprecision
Kupperman index	Total (≤1mo)	537 (5)	MD	**−4.92 [-6.03, -3.80]**	40	Moderate	Risk of bias
Adverse events	Total	1149 (14)	RR	**0.15 [0.07, 0.34]**	64	Moderate	Risk of bias
	≤1mo	909 (12)	RR	**0.13 [0.05, 0.30]**	56	Moderate	Risk of bias
	>1mo ≤ 2mo	120 (1)	RR	0.06 [0.00, 1.00]	NA	Low	Risk of bias, Imprecision
	>2mo	120 (1)	RR	0.67 [0.29, 1.51]	NA	Low	Risk of bias, Imprecision
EAHM *versus* placebo EAHM							
PSQI	Total (≤1mo)	128 (2)	MD	**−5.05 [-9.06, -1.04]**	94	Moderate	Imprecision

AIS, athens insomnia scale; CI, confidence interval; EAHM, east asian herbal medicine; MD, mean difference; NA, not applicable; PSQI, pittsburgh sleep quality index; RCT, randomized controlled trial; RR, risk ratio; SRSS, sleep state self-rating scale; TER, total effective rate.

Bold values mean significant differences between two groups.

### 3.5 Effectiveness and safety of herbal medicine as an adjunctive therapy

1) *Combined with conventional medications for insomnia.* Compared with sedative-hypnotics alone, EAHM plus sedative-hypnotics significantly improved PSQI scores (16 studies, MD –2.46, 95% CI –3.09 to −1.82), TER based on insomnia symptom (20 studies, RR 1.18, 95% CI 1.14–1.23), and Kupperman Index scores (3 studies, MD –4.64, 95% CI –5.07 to −4.21), with no difference in the incidence of adverse events between the two therapies (13 studies, RR 0.70, 95% CI 0.47–1.05) (Table 2). In addition, polysomnography data including total sleep time, rapid-eye-movement sleep time, sleep latency, waking time, and sleep efficiency (%) (Rui et al., 2017), and the Spiegel Sleep Questionnaire scores (Su et al., 2021) significantly improved in the EAHM combination group (p < 0.05, all).
2) *Combined with other psychotropics.* Compared with antidepressants alone, EAHM plus antidepressants significantly improved TER based on insomnia symptoms (two studies, RR 1.16, 95% CI 1.02–1.31), with no difference in incidence of adverse events between the two therapies (two studies, RR 0.52, 95% CI 0.14–1.89) (Table 2). In addition, PSQI also significantly improved after treatment in the EAHM combination group (*p* < 0.05) (Luo et al., 2021).
3) Combined with other conventional medications. Compared with estradiol alone, EAHM combined with other medications significantly improved PSQI and Kupperman Index scores after treatment (p < 0.05, all) (Li X., 2020). In addition, compared with losartan potassium alone, EAHM combined with other medications significantly improved TER based on insomnia symptoms in patients with menopausal insomnia and hypertension (p < 0.05) (Yao et al., 2014).
4) *Subgroup analysis.* Overall, subgroup analysis based on the EAHM administration period did not show significant changes in statistical heterogeneity and effect size (Table 2).

**TABLE 2:** Level of evidence for EAHM as adjuvant therapy.

Outcomes	Subgroup	No. participants (RCTs)	Effect estimate [95% CI]		*I* ^*2*^ value (%)	Certainty of evidence	Reasons for downgrading
EAHM plus sedative-hypnotics *versus* sedative-hypnotics alone							
PSQI	Total	1401 (16)	MD	**−2.46 [-3.09, -1.82]**	95	Low	Risk of bias, Publication bias
	≤1mo	975 (11)	MD	**−2.01 [-2.49, -1.53]**	86	Low	Risk of bias, Publication bias
	>1mo ≤ 2mo	426 (5)	MD	**−3.61 [-4.04, -3.18]**	55	Moderate	Risk of bias
TER (insomnia)	Total	1678 (20)	RR	**1.18 [1.14, 1.23]**	0	Moderate	Risk of bias
	≤1mo	1238 (15)	RR	**1.17 [1.12, 1.22]**	0	Moderate	Risk of bias
	>1mo ≤ 2mo	440 (5)	RR	**1.23 [1.13, 1.33]**	0	Moderate	Risk of bias
Kupperman index	Total	243 (3)	MD	**−4.64 [-5.07, -4.21]**	0	Low	Risk of bias, Imprecision
	≤1mo	97 (1)	MD	**−4.35 [-5.31, -3.39]**	NA	Low	Risk of bias, Imprecision
	>1mo ≤ 2mo	146 (2)	MD	**−4.78 [-5.42, -4.13]**	33	Low	Risk of bias, Imprecision
Adverse events	Total	1118 (13)	RR	0.70 [0.47, 1.05]	0	Low	Risk of bias, Imprecision
	≤1mo	978 (11)	RR	0.68 [0.44, 1.04]	0	Low	Risk of bias, Imprecision
	>1mo ≤ 2mo	140 (2)	RR	0.86 [0.32, 2.33]	NA	Low	Risk of bias, Imprecision
EAHM plus antidepressants *versus* antidepressants alone							
TER (insomnia)	Total	242 (2)	RR	**1.16 [1.02, 1.31]**	0	Low	Risk of bias, Imprecision
	≤1mo	77 (1)	RR	1.15 [0.91, 1.45]	NA	Low	Risk of bias, Imprecision
	>1mo ≤ 2mo	165 (1)	RR	**1.16 [1.01, 1.34]**	NA	Moderate	Risk of bias
Adverse events	Total	245 (2)	RR	0.52 [0.14, 1.89]	68	Low	Risk of bias, Imprecision
	>1mo ≤ 2mo	165 (1)	RR	0.84 [0.55, 1.28]	NA	Low	Risk of bias, Imprecision
	>2mo	80 (1)	RR	**0.22 [0.05, 0.96]**	NA	Low	Risk of bias, Imprecision

CI, confidence interval; EAHM, east asian herbal medicine; MD, mean difference; NA, not applicable; PSQI, pittsburgh sleep quality index; RCT, randomized controlled trial; RR, risk ratio; TER, total effective rate.

Bold values mean significant differences between two groups.

### 3.6 Publication bias

**FIGURE 3:**
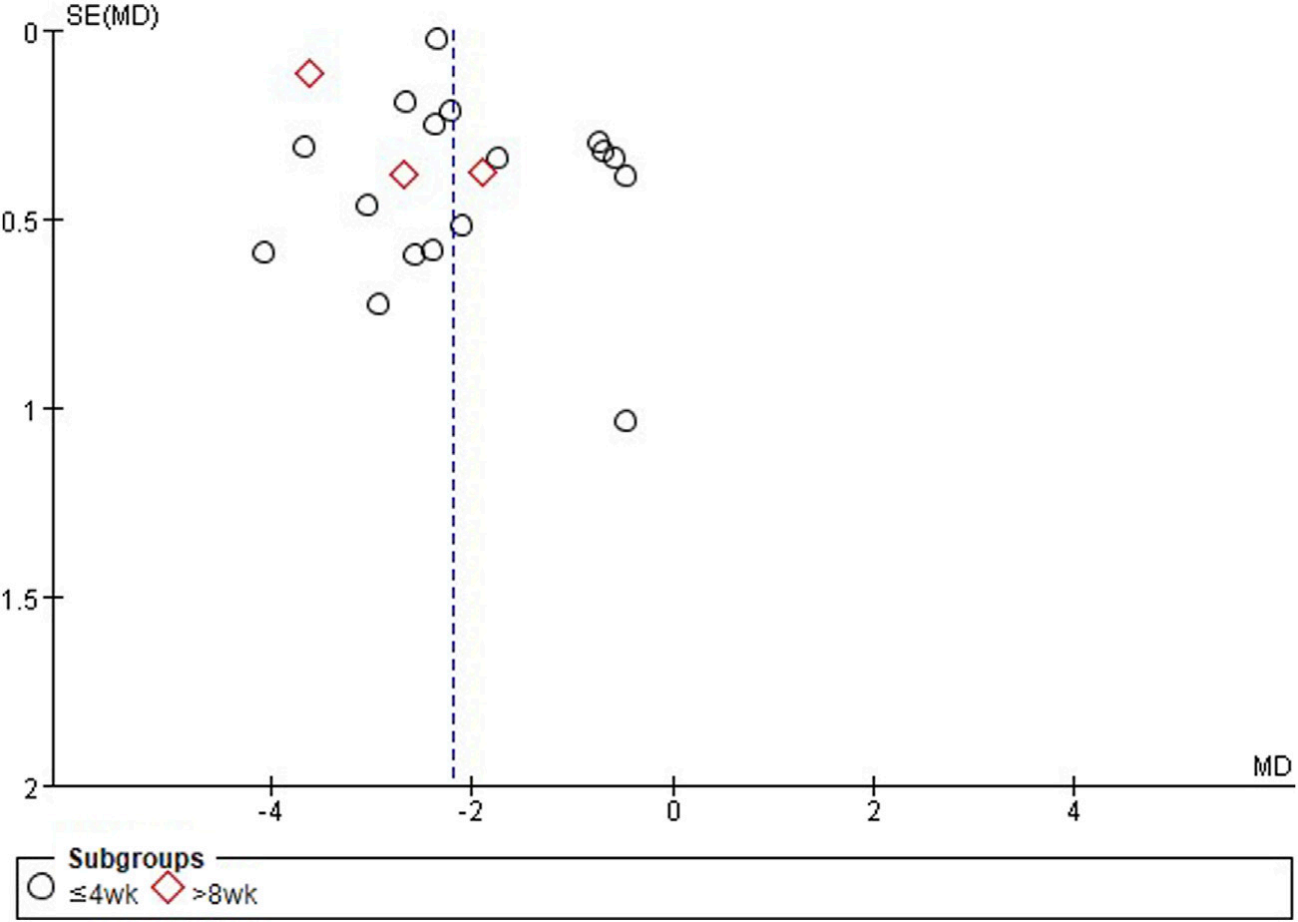
Title: Funnel plots of EAHM compared with sedative-hypnotics on PSQI. Footnotes: EAHM, East Asian herbal medicine; PSQI, Pittsburgh sleep quality index.

According to the funnel plots and Egger’s tests, when comparing EAHM and sedative-hypnotics, the results did not suggest publication bias in PSQI scores (*p* = 0.667) and adverse events (*p* = 0.058) (Figure 3); however, publication bias was suggested in TER based on insomnia symptom (*p* = 0.001). When comparing EAHM plus sedative-hypnotics and sedative-hypnotics alone, publication bias was suggested in PSQI scores (*p* = 0.043) but not in TER based on insomnia symptoms (*p* = 0.151) and adverse events (*p* = 0.52) (Supplementary Material S7).

### 3.7 Certainty of evidence

The certainty of evidence for effect estimates comparing EAHM with sedative-hypnotics and comparing EAHM plus sedative-hypnotics with sedative-hypnotics alone was generally moderate to low because of the risk of bias of the included studies, imprecision due to small sample size and wide confidence intervals, and publication bias. The certainty of evidence for effect estimates comparing EAHM with placebo EAHM was moderate because of the imprecision due to the small sample size (Tables 1, 2).

## 4 Discussion

### 4.1 Summary of findings

Our main finding is that EAHM is uniquely effective in the treatment of menopausal disorders. Specifically, according to the meta-analysis, EAHM as a monotherapy was not only significantly effective in improving sleep quality, but it also showed significant superiority in terms of Kupperman Index score improvement and safety profile compared to sedative-hypnotics. As an adjuvant therapy, EAHM showed significantly better results in improving both sleep quality and Kupperman Index scores than those of conventional medicine alone. However, the methodological quality of the included studies was limited, and the certainty of evidence assessed based on GRADE ranged from “very low” to “moderate,” with no case being evaluated as “high.”

### 4.2 Clinical interpretation

Based on our current findings, for patients with menopausal insomnia, EAHM, as monotherapy or adjuvant therapy, was significantly more effective in improving sleep quality and menopausal syndrome symptoms compared with sedative-hypnotics alone. The superiority of EAHM may be due to the unique pathology of menopausal insomnia. Specifically, menopausal insomnia is associated with vasomotor and physical menopausal symptoms in this population (Kim et al., 2018; DePree et al., 2023). In this context, improvement of menopausal symptoms may contribute to the treatment of menopausal insomnia. EAHM is composed of various botanical drugs, and its therapeutic mechanism is characterized by multiple components-multiple targets-multiple pathways (Zhang et al., 2019). Based on our findings, the most frequently used botanical drug in EAHM for the treatment of menopausal insomnia is *Zizyphus jujuba M.* var. *spinosa Hu ex H. F. Chou* [Rhamnaceae; Zizyphi Semen], and this has been confirmed by other researchers (Liu et al., 2015). Additionally, during a double-blind RCT conducted in Iran, capsules of this botanical drug were compared with a placebo and found that they improve sleep quality in postmenopausal women (Mahmoudi et al., 2020). Interestingly, this botanical drug was one of the most frequently studied botanical drugs as a component of EAHM for menopausal symptoms (Zhu et al., 2016). During an *in silico* study where the anti-insomnia mechanism of EAHMs was investigated, including this botanical drug (i.e., the Suan Zao Ren Prescription), the researchers found that most of the EAHMs exert broad hormone-regulating effects by targeting estrogen receptors, progesterone receptors, and adrenergic receptors (Gao et al., 2019). We found that the second most commonly used botanical drug was *G. glabra L.* [Fabaceae; Glycyrrhizae Radix et Rhizoma], and its effect on improving sleep and menopausal symptoms has been reported. Glabrol, one of the main flavonoid metabolites of this botanical drug, was found to have a sleep-inducing effect through a positive allosteric modulation of γ-aminobutyric acid (GABA) type A-benzodiazepine receptors (Cho et al., 2012). Additionally, this botanical drug contains liquiritigenin, a phytoestrogen, in various amounts depending on the species, and is known to have partial estrogen agonist activity (Hajirahimkhan et al., 2013b). These multiple pharmacological actions of EAHM can be considered as the underlying mechanism that may be responsible for the improved effectiveness of EAHM compared with sedative-hypnotics in menopausal insomnia.

The results of the current review show that EAHM was superior to estradiol in improving sleep quality but inferior in improving the Kupperman Index scores. This suggests that EAHM can be considered an effective alternative when insomnia is present as a menopausal symptom. Additionally, although not investigated in this study, EAHM is reported to be effective in improving menopause-related health problems, such as sleep deprivation and osteoporosis (Yi et al., 2018). Meanwhile, it has been shown that phytoestrogens, contained in some botanical drugs, can increase sex hormone levels, although the levels are much lower than those induced by hormone therapy; therefore, caution is needed when prescribing to individuals with contraindications (Saghafi et al., 2017). The use of dietary supplements, including herbal products, to treat menopausal disorders is common, and it is often done without consultation with doctors or pharmacists (Chiba, 2023). Therefore, it is advisable to ensure the safe use of EAHM in the menopausal population under the guidance of an expert such as a KM doctor who has sufficient knowledge about TEAM. If EAHM cannot be used, acupressure or acupuncture and other modalities of TEAM can be considered as they are known to be effective in improving menopausal symptoms (Ebrahimi et al., 2020; Zhao et al., 2021).

Based on our current findings, the combination of EAHM and conventional medication showed better results than conventional medication alone in most cases, including sleep quality and the Kupperman Index scores. Despite these positive findings, the herb-drug interaction should be considered and their combination is recommended in clinical settings. For instance, *Zizyphus jujuba M.* var. *spinosa Hu ex H. F. Chou* [Rhamnaceae; Zizyphi Semen], the botanical drug most frequently used in the studies included in this review, may have mechanisms similar to that of sedative-hypnotics by affecting GABAergic signaling (Bruni et al., 2021). According to a previous study, concurrent use of EAHMs and sedative-hypnotics is associated with a favorable safety profile, including a reduced risk of hip fracture in individuals with insomnia (Lee et al., 2013). Moreover, a recent clinical trial revealed that the combined use of EAHM, commonly used for insomnia, and zolpidem tartrate increased efficacy without a significant change in the occurrence of adverse events (Zhu et al., 2022). It is desirable to disseminate information from an evidence-based medicine perspective on the interaction between EAHM and sedative-hypnotics among both prescribing doctors and the public.

Phytoestrogens have been shown to not only improve menopausal symptoms but also have positive benefits for the health of various organs, and they are considered an alternative to hormone replacement therapy (Desmawati and Sulastri, 2019). However, the therapeutic effect of EAHM on menopause cannot be explained solely by the estrogenic effect. EAHM may also affect the improvement of menopausal symptoms through non-estrogenic mechanisms, such as alleviation of oxidative stress, inhibition of prostaglandin D2 receptor 1, and serotonergic activities (Hajirahimkhan et al., 2013a; Tsoumani et al., 2022). Additionally, in this population using estrogen plus progestin, the use of EAHM is not significantly associated with an increased risk of breast cancer (Tsai et al., 2014) but rather with a decreased risk (Tsai et al., 2017). In general, the use of EAHM in the menopausal population seems to be safe, but information sharing and evidence-based education with physicians are important (Chiba, 2023). The East Asian countries using TEAM already operate a system of medical personnel who specialize in EAHM, highlighting their important role in the healthcare of menopausal women.

### 4.3 Suggestions for further studies

Based on the findings from this review, we propose the following recommendations for further research. First, considering that the effect of EAHM on menopausal insomnia is characterized by multiple components-multiple targets-multiple pathways (Zhang et al., 2019) and EAHM based on TEAM is not composed of a single botanical drug but of multiple botanical drugs, understanding the interaction between botanical drugs in therapeutic mechanism is crucial. For instance, *G. glabra L.* [Fabaceae; Glycyrrhizae Radix et Rhizoma] and *Ziziphus jujuba Mill.* [Rhamnaceae; Zizyphi Fructus] are an important combination in the treatment of menopausal symptoms, and the sedative, antidepressant, estrogenic, and antiprogestogenic effects of this combination have been revealed through non-clinical experiments (Coyle et al., 2021). However, there are limited studies that consider the therapeutic mechanism of the combination of important botanical drugs in menopausal insomnia, and this is an important future research area. Second, efficacy of EAHM in improving insomnia in patients with menopausal insomnia requires a thorough understanding. This includes investigating not only the direct effects of EAHM on insomnia but also the secondary effect through the improvement of other menopausal symptoms. For instance, an attempt could be made to further analyze the relationship between the changes in patients’ menopausal symptoms and degree of improvement in insomnia. Third, uncovering the differential effects of EAHM on menopausal symptoms, including menopausal insomnia, will enhance the effective use of EAHM in future clinical settings. Fourth, because this systematic review targeted RCTs that investigated the effectiveness and safety of EAHM on menopausal insomnia, findings on the underlying therapeutic mechanisms of EAHM used are lacking. Future literature review will be able to clearly define the therapeutic mechanism of EAHM for menopausal insomnia by comprehensively examining related preclinical experiments.

### 4.4 Limitations

We acknowledge the following limitations in our systematic review. First, the overall risk of bias in the included studies was not optimal. Although this review included RCTs with appropriate randomization, most of the other risk of bias domains showed unfavorable methodological quality. Specifically, clinical trials using placebo were rare, and only one trial implemented blinding of the outcome assessor. Therefore, the results obtained in these studies are potentially vulnerable to the placebo effect. Owing to the methodological limitations of most of the included studies, the certainty of evidence assessed by GRADE was also generally moderate to low. This suggests that our findings could be greatly influenced by future high-quality research in this field. Additionally, the fact that most of the included studies were published in local journals in China suggests a potential risk of publication bias (Jia et al., 2020). Second, the composition of EAHMs used in the included studies was heterogeneous. Therefore, it was not possible to find the optimal EAHM for menopausal insomnia in this review. Third, because most of the included studies were conducted in China, these results are difficult to generalize to the global population. This is because familiarity, knowledge, and attitudes toward EAHM based on TEAM may differ between Chinese and non-Asian populations. Fourth, among the included studies, no studies that reported on insomnia severity using categorizable indicators such as ISI, so subgroup analysis according to insomnia severity was not possible. However, further investigation is needed to determine the possibility of differential effects of EAHM according to baseline insomnia severity in menopausal insomnia patients.

## 5 Conclusion

This systematic review is the most comprehensive and recent work examining the effectiveness and safety of EAHM for menopausal insomnia. The current findings provide limited evidence that EAHM, based on TEAM, may help improve both sleep quality and other menopausal symptoms for treating menopausal insomnia. Specifically, EAHM showed significantly superior effects in improving sleep quality and menopausal symptoms in individuals with menopausal insomnia as monotherapy or combination therapy with sedative-hypnotics, and had few adverse reactions. However, the certainty of the evidence is moderate to low, indicating a need for further high-quality research. Future studies should aim to improve the quality by implementing blinding of participants, investigators, and outcome assessors. Additionally, a deeper understanding of the mechanisms of action of EAHM is needed. Expanding research to include diverse populations is essential to confirm the generalizability of these findings. These steps will be critical in substantiating the potential of EAHM as a valuable treatment option for menopausal insomnia.

## Data Availability

The original contributions presented in the study are included in the article/Supplementary Material, further inquiries can be directed to the corresponding author.

## References

[B1] Bai H. Wang J. Liu F. (2014). Clinical observation on the treatment of refractory insomnia in perimenopausal women with bushen lemian decoction. Shaanxi J. Traditional Chin. Med. 35, 666–668.

[B2] Baker F. C. Lampio L. Saaresranta T. Polo-Kantola P. (2018). Sleep and sleep disorders in the menopausal transition. Sleep. Med. Clin. 13, 443–456. 10.1016/j.jsmc.2018.04.011 30098758 PMC6092036

[B3] Balshem H. Helfand M. Schünemann H. J. Oxman A. D. Kunz R. Brozek J. (2011). GRADE guidelines: 3. Rating the quality of evidence. J. Clin. Epidemiol. 64, 401–406. 10.1016/j.jclinepi.2010.07.015 21208779

[B4] Bastien C. H. Vallières A. Morin C. M. (2001). Validation of the Insomnia Severity Index as an outcome measure for insomnia research. Sleep. Med. 2, 297–307. 10.1016/s1389-9457(00)00065-4 11438246

[B5] Bruni O. Ferini-Strambi L. Giacomoni E. Pellegrino P. (2021). Herbal remedies and their possible effect on the GABAergic system and sleep. Nutrients 13, 530. 10.3390/nu13020530 33561990 PMC7914492

[B6] Buysse D. J. Reynolds Iii C. F. Monk T. H. Berman S. R. Kupfer D. J. (1989). The Pittsburgh Sleep Quality Index: a new instrument for psychiatric practice and research. Psychiatry Res. 28, 193–213. 10.1016/0165-1781(89)90047-4 2748771

[B7] Cai Y. Ye Y.-M. Zhang T. Yang W. Wang G.-Y. Shi W. (2019). Clinical efficacy of modified guizhi gancao Longgu Muli Tang in treating menopausal insomnia and its effect on sleep quality and neurotransmitter. Chin. J. Exp. Traditional Med. Formulae 25, 38–42.

[B8] Chen H. Yang Y. (2012). 42 cases of perimenopausal insomnia treated with integrated traditional Chinese and Western medicine. Fujian J. Traditional Chin. Med. 43, 33–34.

[B9] Chen X. Du X.-F. (2007). Nourishing Kidney and Soothing Heart method for treating perimenopausal insomnia. Pract. J. Clin. Med. 4, 60–61.

[B10] Chen Y. Li T. Chen Y. Lin Y. (2019). Clinical study on shugan jianpi multi-level therapy for patients with insomnia in perimenopause. Chin. J. Inf. TCM 26, 18–22.

[B11] Chiba T. (2023). Patients are using dietary supplement for the treatment of their diseases without consultation with their physicians and pharmacists. Pharm. (Basel) 11, 179. 10.3390/pharmacy11060179 PMC10661250 37987389

[B12] Cho S. Park J. H. Pae A. N. Han D. Kim D. Cho N. C. (2012). Hypnotic effects and GABAergic mechanism of licorice (Glycyrrhiza glabra) ethanol extract and its major flavonoid constituent glabrol. Bioorg Med. Chem. 20, 3493–3501. 10.1016/j.bmc.2012.04.011 22543233

[B13] Ciano C. King T. S. Wright R. R. Perlis M. Sawyer A. M. (2017). Longitudinal study of insomnia symptoms among women during perimenopause. J. Obstet. Gynecol. Neonatal Nurs. 46, 804–813. 10.1016/j.jogn.2017.07.011 PMC5776689 28886339

[B14] Coyle M. E. Liu J. Yang H. Wang K. Zhang A. L. Guo X. (2021). Licorice (Glycyrrhiza spp.) and jujube (Ziziphus jujuba Mill.) formula for menopausal symptoms: classical records, clinical evidence and experimental data. Complement. Ther. Clin. Pract. 44, 101432. 10.1016/j.ctcp.2021.101432 34237667

[B15] Depree B. Shiozawa A. King D. Schild A. Zhou M. Yang H. (2023). Association of menopausal vasomotor symptom severity with sleep and work impairments: a US survey. Menopause 30, 887–897. 10.1097/GME.0000000000002237 37625086 PMC10487384

[B16] Desmawati D. Sulastri D. (2019). Phytoestrogens and their health effect. Open Access Maced. J. Med. Sci. 7, 495–499. 10.3889/oamjms.2019.044 30834024 PMC6390141

[B17] Ebrahimi A. Tayebi N. Fatemeh A. Akbarzadeh M. (2020). Investigation of the role of herbal medicine, acupressure, and acupuncture in the menopausal symptoms: an evidence-based systematic review study. J. Fam. Med. Prim. Care 9, 2638–2649. 10.4103/jfmpc.jfmpc_1094_19 PMC7491766 32984100

[B18] Gao J. Wang Q. Huang Y. Tang K. Yang X. Cao Z. (2019). *In silico* study of anti-insomnia mechanism for suanzaoren prescription. Front. Pharmacol. 10, 925. 10.3389/fphar.2019.00925 31507421 PMC6713715

[B19] Geng J. Yu Q. (2014). Observation on the efficacy of modified Danggui Liuhuang Decoction in the treatment of 32 cases of female menopausal insomnia. J. Traditional Chin. Med. 55, 1581–1583.

[B20] Guo F. (2016). Discussion on the application value of Anshen Decoction in patients with sleep disorders in menopausal women. World Latest Med. Inf. 16, 132 + 131.

[B21] Guo Q. Cheng Y. Zhang C. Yang H. Chen X. Wang X. (2022). A search of only four key databases would identify most randomized controlled trials of acupuncture: a meta-epidemiological study. Res. Synth. Methods 13, 622–631. 10.1002/jrsm.1581 35716041

[B22] Hajirahimkhan A. Dietz B. M. Bolton J. L. (2013a). Botanical modulation of menopausal symptoms: mechanisms of action? Planta Med. 79, 538–553. 10.1055/s-0032-1328187 23408273 PMC3800090

[B23] Hajirahimkhan A. Simmler C. Yuan Y. Anderson J. R. Chen S. N. Nikolić D. (2013b). Evaluation of estrogenic activity of licorice species in comparison with hops used in botanicals for menopausal symptoms. PLoS One 8, e67947. 10.1371/journal.pone.0067947 23874474 PMC3709979

[B24] Higgins J. P. Altman D. G. Gøtzsche P. C. Jüni P. Moher D. Oxman A. D. (2011). The Cochrane Collaboration's tool for assessing risk of bias in randomised trials. Bmj 343, d5928. 10.1136/bmj.d5928 22008217 PMC3196245

[B25] Hu L. (2019). Analysis of the effect of huanglian ejiao decoction combined with ganmai dazao decoction in the treatment of menopausal insomnia. Inn. Mongol J. Traditional Chin. Med. 38, 49–50.

[B26] Hu L. (2020). Analysis of the efficacy of Shengyu Decoction in treating menopausal syndrome combined with insomnia. J. Med. Theory Pract. 33, 1279–1281.

[B27] Hua L. (2017). Clinical efficacy of Jieyu Anshen Decoction on menopausal insomnia in women. J. Pract. Gynecol. Endocrinol. 4, 102 + 104.

[B28] Huang X.-Q. Li F.-M. Liang Z.-Q. (2020). Clinical value of Heixiaoyao Powder combined with Shule Anding in treating female perimenopausal insomnia and its effect on neuroendocrine system. World J. Integr. Traditional West. Med. 15, 1688–1691.

[B29] Jia L. Tang L. Liu Q. Wang Y. Ma H. (2023). Clinical effect of gengxin decoction on perimenopausal insomnia with heart-kidney non-interaction syndrome. World Chin. Med. 18, 839–843.

[B30] Jia X. Yang X. (2008). 110 cases of menopausal insomnia treated with Gengnian Anshen Decoction and Mirtazapine. Traditional Chin. Med. Res. 21, 30–31.

[B31] Jia Y. (2014). Observation on the efficacy of self-prepared traditional Chinese medicine combined with alprazolam in the treatment of 40 cases of female menopausal insomnia. Med. J. Chin. People's Health 26, 71–72.

[B32] Jia Y. Huang D. Wen J. Wang Y. Rosman L. Chen Q. (2020). Assessment of language and indexing biases among Chinese-sponsored randomized clinical trials. JAMA Netw. Open 3, e205894. 10.1001/jamanetworkopen.2020.5894 32463469 PMC7256669

[B33] Jun J. H. Lee H. W. Choi J. Choi T. Y. Lee J. A. Go H. Y. (2019). Perceptions of using herbal medicines for managing menopausal symptoms: a web-based survey of Korean medicine doctors. Integr. Med. Res. 8, 229–233. 10.1016/j.imr.2019.08.004 31646139 PMC6804440

[B34] Khadivzadeh T. Abdolahian S. Ghazanfarpour M. Kargarfard L. Dizavandi F. R. Khorsand I. (2018). A systematic review and meta-analysis on the effect of herbal medicine to manage sleep dysfunction in peri- and postmenopause. J. Menopausal Med. 24, 92–99. 10.6118/jmm.2018.24.2.92 30202758 PMC6127017

[B35] Kim D. Shih C. C. Cheng H. C. Kwon S. H. Kim H. Lim B. (2021). A comparative study of the traditional medicine systems of South Korea and Taiwan: focus on administration, education and license. Integr. Med. Res. 10, 100685. 10.1016/j.imr.2020.100685 33665088 PMC7903058

[B36] Kim M. J. Yim G. Park H. Y. (2018). Vasomotor and physical menopausal symptoms are associated with sleep quality. PLoS One 13, e0192934. 10.1371/journal.pone.0192934 29462162 PMC5819793

[B37] Kravitz H. M. Joffe H. (2011). Sleep during the perimenopause: a SWAN story. Obstet. Gynecol. Clin. North Am. 38, 567–586. 10.1016/j.ogc.2011.06.002 21961720 PMC3185248

[B38] Kwon C.-Y. Lee B. Chung S.-Y. Kim J. W. Kim S.-H. (2019). Herbal medicine for insomnia in elderly with hypertension: a systematic review and meta-analysis. Eur. J. Integr. Med. 30, 100961. 10.1016/j.eujim.2019.100961

[B39] Lai M. Ma H. Liu H. Xie J. Ding T. (2011). Observation on the efficacy of modified Ji Kuang Meng Xing Decoction in the treatment of 40 cases of perimenopausal insomnia in women. Int. J. Traditional Chin. Med. 33, 250–251.

[B40] Lai X. He L. Chen L. (2022). Clinical efficacy of Zishui Bugan Decoction combined with eszopiclone in the treatment of perimenopausal insomnia due to liver and kidney deficiency syndrome. Shenzhen J. Integr. Traditional Chin. West. Med. 32, 41–43.

[B41] Lan J. (2020). Clinical study on Xiaochaihu decoction in treating menopausal insomnia. China Health Care and Nutr. 30, 358.

[B42] Lee K. H. Tsai Y. T. Lai J. N. Lin S. K. (2013). Concurrent use of hypnotic drugs and Chinese herbal medicine therapies among Taiwanese adults with insomnia symptoms: a population-based study. Evid. Based Complement. Altern. Med. 2013, 987862. 10.1155/2013/987862 PMC3800591 24204397

[B43] Lei J. (2013). Clinical observation on treating menopausal syndrome insomnia with nourishing the kidney and soothing the liver method. Fujian J. Traditional Chin. Med. 44, 11–12.

[B44] Li C. Gu X. (2018). Clinical study of Qiju Dihuang pills combined with losartan potassium tablets for menopause insomnia with hypertension. J. New Chin. Med. 50, 61–63.

[B45] Li K. (2016). Modified suanzaorentang 56 cases improve the quality of sleep in patients with insomnia menopause effectiveness analysis. Syst. Med. 1, 31–33.

[B46] Li M. Hung A. Lenon G. B. Yang A. W. H. (2019). Chinese herbal formulae for the treatment of menopausal hot flushes: a systematic review and meta-analysis. PLoS One 14, e0222383. 10.1371/journal.pone.0222383 31536531 PMC6752783

[B47] Li T. Zhang Y. (2022). Clinical study on stellate ganglion block combined with Kuntai capsule in the treatment of perimenopausal sleep disorder accompanied by anxiety state. Maternal Child Health Care China 37, 3656–3659.

[B48] Li W. Luo H. (2015). “Observation on the efficacy of Gengnian Anshen Cream in the treatment of perimenopausal insomnia due to heart-kidney disharmony,” in Proceedings of the 2015 academic annual conference of the gansu provincial society of integrated traditional Chinese and western medicine. (Lanzhou).

[B49] Li X. (2020a). Effects of Kuntai capsule on mental state and sleep quality in menopausal syndrome patients with insomnia. J. Liaoning Univ. TCM 22, 162–165.

[B50] Li X. Chen W. Simal-Gandara J. Georgiev M. I. Li H. Hu H. (2021). West meets east: open up a dialogue on phytomedicine. Chin. Med. 16, 57. 34281584 10.1186/s13020-021-00467-6 PMC8287783

[B51] Li X.-Q. (2021). Clinical evaluation of ganmai dazhao decoction and baihe Dihuang decoction in the treatment of perimenopausal insomnia. World Latest Med. Inf. 21, 161–163.

[B52] Li Z. (2020b). Clinical observation on the treatment of menopausal insomnia with Xiaochaihu decoction. China Health Care and Nutr. 30, 91–92.

[B53] Liu L. (2021). Effects of anshen yangxin paste combined with cognitive behavioral therapy on IL-1β, IL-2, IL-6 levels in perimenopausal patients with sleep disorders. World J. Sleep Med. 8, 1343–1345.

[B54] Liu L. Liu C. Wang Y. Wang P. Li Y. Li B. (2015). Herbal medicine for anxiety, depression and insomnia. Curr. Neuropharmacol. 13, 481–493. 10.2174/1570159x1304150831122734 26412068 PMC4790408

[B55] Lu H. (2019). The efficacy of modified Guiganlongmu decoction in treating menopausal insomnia due to yin deficiency type. Health Care Today, 184–185.

[B56] Lu J. (2007). Observation on the efficacy of modified Ganmai Dazao decoction in treating insomnia in menopausal women. J. Third Mil. Med. Univ., 1634–1635.

[B57] Luo J. Wu Y. Song L. Chen X. (2021). Effect of Danzhi Xiaoyao Pills combined with Flupentixol and Melitracen Tablets in the treatment of perimenopausal depression combined with sleep disorders. Shenzhen J. Integr. Traditional Chin. West. Med. 31, 55–57.

[B58] Ma X. (2018). Analysis of the effect of Tiaochong Anshen Decoction on sleep quality in patients with menopausal syndrome and insomnia. World Latest Med. Inf. 18, 142–143.

[B59] Mahmoudi R. Ansari S. Haghighizadeh M. H. Maram N. S. Montazeri S. (2020). Investigation the effect of jujube seed capsule on sleep quality of postmenopausal women: a double-blind randomized clinical trial. Biomed. (Taipei) 10, 42–48. 10.37796/2211-8039.1038 PMC7735973 33854934

[B60] Mao F. (2020). Effects of modified Huanglian Ejiao decoction on sleep quality and sex hormone levels in perimenopausal patients with insomnia. Maternal Child Health Care China 35, 4297–4300.

[B61] Marshall A. C. (2020). Traditional Chinese medicine and clinical pharmacology. Drug Discov. Eval. Methods Clin. Pharmacol., 455–482. 10.1007/978-3-319-68864-0_60

[B62] Mi X. Fang J. Yu X. Luo Z. Tang J. Chen J. (2022). Clinical efficacy of zishui bugan decoction on perimenopausal insomnia patients with liver-kidney deficiency. Chin. J. Exp. Traditional Med. Formulae 28, 116–122.

[B63] Monteleone P. Mascagni G. Giannini A. Genazzani A. R. Simoncini T. (2018). Symptoms of menopause - global prevalence, physiology and implications. Nat. Rev. Endocrinol. 14, 199–215. 10.1038/nrendo.2017.180 29393299

[B64] Ni X. Shergis J. L. Guo X. Zhang A. L. Li Y. Lu C. (2015). Updated clinical evidence of Chinese herbal medicine for insomnia: a systematic review and meta-analysis of randomized controlled trials. Sleep. Med. 16, 1462–1481. 10.1016/j.sleep.2015.08.012 26611943

[B65] Page M. J. Mckenzie J. E. Bossuyt P. M. Boutron I. Hoffmann T. C. Mulrow C. D. (2021). The PRISMA 2020 statement: an updated guideline for reporting systematic reviews. Bmj 372, n71. 10.1136/bmj.n71 33782057 PMC8005924

[B66] Pan X. Sun T. Yang J. Liu W. (2022). Observation on the effect of modified Bupleurum plus Longgu Oyster Decoction in treating menopausal insomnia due to liver qi stagnation. Mod. Med. Health Res. Electron. J. 6, 95–98.

[B67] Pang Y. (2019). To explore the therapeutic effect of traditional Chinese medicine Anshen Pills on menopausal insomnia in women. Inn. Mongol J. Traditional Chin. Med. 38, 18–20.

[B68] Park H. L. Lee H. S. Shin B. C. Liu J. P. Shang Q. Yamashita H. (2012). Traditional medicine in China, Korea, and Japan: a brief introduction and comparison. Evid. Based Complement. Altern. Med. 2012, 429103. 10.1155/2012/429103 PMC3486438 23133492

[B69] Posadzki P. Lee M. S. Moon T. W. Choi T. Y. Park T. Y. Ernst E. (2013). Prevalence of complementary and alternative medicine (CAM) use by menopausal women: a systematic review of surveys. Maturitas 75, 34–43. 10.1016/j.maturitas.2013.02.005 23497959

[B70] Pu H. Wang Y. (2019). Observation on the efficacy of Buxin Xiaoyao Drink in treating perimenopausal insomnia in women. World Latest Med. Inf. 19, 230–231.

[B71] Qiao W. Gao Y. Sun K.-F. Ding J.-J. Wang D. Chen H.-J. (2021). Clinical study of modified treatment with tianwang buxin pills plus jiaotai pills for perimenopausal hypertension accompanied by insomnia. J. Guangzhou Univ. Traditional Chin. Med. 38, 1840–1846.

[B72] Qin G. (2023). Clinical study on modified wendan decoction combined with Chaihu shugan powder for menopause insomnia. New Chin. Med. 55, 72–75.

[B73] Rui M. Wang L. Wang C. Hou Y. Song L. Cao L. (2017). Study on the clinical efficacy of modified Qinggan Xiexin Decoction in the treatment of perimenopausal insomnia and its impact on serum 5-HT levels. Mod. J. Integr. Traditional Chin. West. Med. 26, 2904–2906.

[B74] Saghafi N. Ghazanfarpour M. Sadeghi R. Hosseini Najarkolaei A. Ghaffarian Omid M. Azad A. (2017). Effects of phytoestrogens in alleviating the menopausal symptoms: a systematic review and meta-analysis. Iran. J. Pharm. Res. 16, 99–111. 29844781 PMC5963651

[B75] Salari N. Hasheminezhad R. Hosseinian-Far A. Rasoulpoor S. Assefi M. Nankali S. (2023). Global prevalence of sleep disorders during menopause: a meta-analysis. Sleep. Breath. 27, 1883–1897. 10.1007/s11325-023-02793-5 36892796 PMC9996569

[B76] Shan Y. Du S.-H. (2020). Clinical observation of modified yanghe decoction combined with estazolam for treatment climacteric sleep disorders. J. Guangzhou Univ. Traditional Chin. Med. 37, 1658–1662.

[B77] Shen Y. Wang Z. (2018). Shugan jianpi decoctionin treatment of sleep disorders in perimenopausal patients andKupperman, SAS and SDS scores. Chin. Archives Traditional Chin. Med. 36, 496–498.

[B78] Shi M. (2018). Clinical observation on the treatment of perimenopausal insomnia with zishen jieyu ningxin rcecipe. Yiyao Qianyan 8, 365–366.

[B79] Shi M. M. Piao J. H. Xu X. L. Zhu L. Yang L. Lin F. L. (2016). Chinese medicines with sedative-hypnotic effects and their active components. Sleep. Med. Rev. 29, 108–118. 10.1016/j.smrv.2015.10.001 26866454

[B80] Su W. Zhang L. Ji J. Liu A. Ye J. Tang J. (2021). Observation on the clinical effect of erxian decoction on the treatment of menopausal insomnia with kidney deficiency. China Health Stand. Manag. 12, 117–120.

[B81] Sun K. Tian Y. (2020). Analysis of the clinical therapeutic effect of Suanzaoren Decoction on menopausal patients with insomnia. Gems Health 40.

[B82] Sun X. Huang H. (2016). Clinical study on the treatment of perimenopausal insomnia with phlegm-heat internal disturbance by modified Wendan decoction. J. New Chin. Med. 48, 115–116.

[B83] Tsai Y. T. Lai J. N. Lo P. C. Chen C. N. Lin J. G. (2017). Prescription of Chinese herbal products is associated with a decreased risk of invasive breast cancer. Med. Baltim. 96, e7918. 10.1097/MD.0000000000007918 PMC5585506 28858112

[B84] Tsai Y. T. Lai J. N. Wu C. T. Lin S. K. (2014). Concurrent use in taiwan of Chinese herbal medicine therapies among hormone users aged 55 Years to 79 Years and its association with breast cancer risk: a population-based study. Evid. Based Complement. Altern. Med. 2014, 683570. 10.1155/2014/683570 PMC4058844 24987432

[B85] Tsoumani M. Nikolaou P. E. Argyropoulou A. Tseti I. Mitakou S. Andreadou I. (2022). Novel evidence-based combination of plant extracts with multitarget mechanisms of action for the elimination of hot flashes during menopause. Molecules 27, 1221. 10.3390/molecules27041221 35209016 PMC8874944

[B86] Wang F. Duan J. Wang B. Cui S. (2018a). Clinical observation on the treatment of perimenopausal insomnia due to kidney deficiency and liver stagnation with modified Ganmai Dazao Decoction. Chin. J. Traditional Med. Sci. Technol. 25, 106–107.

[B87] Wang J. (2014). Observation on the clinical effect of modified Suanzaoren Decoction in the treatment of menopausal insomnia patients. China Health Ind. 11, 180–181.

[B88] Wang J. Wang D. (2022). Effects of modified Bupleurum plus Longgu Oyster Decoction on sleep quality, negative emotions and endocrine hormones in patients with perimenopausal insomnia. Mod. J. Integr. Traditional Chin. West. Med. 31, 1842–1845.

[B89] Wang L. Fan L. Zhao Y. Ma A. (2018b). Observation on the efficacy of Qingxin Zhengan Decoction in treating perimenopausal insomnia. J. Imaging Res. Med. Appl. 2, 228–229.

[B90] Wang M.-R. Fang Z.-H. Han H. Ding X.-J. (2021a). Effect of modified Yiganxue Suanzaoren Decoction on levels of sex hormones and sleep quality of perimenopausal women with insomnia. J. Hunan Norm. Univ. 18, 9–13.

[B91] Wang Q. Ye X. Huang X. (2021b). The efficacy of modified Bupleurum plus Longgu Oyster Decoction in the treatment of menopausal insomnia and its improvement in clinical symptoms. Inn. Mongol J. Traditional Chin. Med. 40, 47–48.

[B92] Wang X. Zhu L. Yuan Y. (2022). Yin-nourishing fire-purging decoction in treating 39 cases of sleep disorders in menopausal women. West. J. Traditional Chin. Med. 35, 100–102.

[B93] Wang Y. Ma K. (2023). Clinical study on Liuwei Dihuang decoction combined with modified Xiaoyao powder in the treatment of perimenopausal sleep disorder of kidney deficiency and liver depression. Shaanxi J. Traditional Chin. Med. 44, 584–586 + 611.

[B94] Wei J. Yang X. (2020). Effect of supplemented Chaihu Guizhi Longgu Muli Decoction in treatment of insomnia induced by anxiety in perimenopausal period. J. Clin. Med. Pract. 24, 43–45.

[B95] Weng X. (2016). Clinical curative observation of using suanzao baihe decoction in the treatment of menopause insomnia with blood and yin deficiency type. J. Sichuan Traditional Chin. Med. 34, 180–182.

[B96] Wu T. Li Y. Bian Z. Liu G. Moher D. (2009). Randomized trials published in some Chinese journals: how many are randomized? Trials 10, 46. 10.1186/1745-6215-10-46 19573242 PMC2716312

[B97] Xing J. (2018). Clinical observation of flavoring antihepatic powder therapeutic effect to perimenopausal insomnia in female. World J. Sleep Med. 5, 662–664.

[B98] Xu W. Zhou Y. Jiang R. Wang Q. Qin Y. (2023). Clinical efficacy of tonifying kidney, soothing liver and tranquilizing spirit in treatment of perimenopausal insomnia. Shaanxi J. Traditional Chin. Med. 44, 41–44.

[B99] Yao X. Hu W. Qian Y. Cheng Z. (2014). Clinical analysis of 45 cases of menopausal hypertension combined with insomnia treated with Guidibaihe decoction combined with Losartan. J. New Chin. Med. 46, 57–58.

[B100] Ye W. Ye R. Yuan Z. Wang H. Ni X. Fang M. (2015). Clinical observation on the treatment of perimenopausal insomnia with modified jiayi guizang decoction. Guangxi J. Traditional Chin. Med. 38, 19–20.

[B101] Yi S. S. Chung S. H. Kim P. S. (2018). Sharing pathological mechanisms of insomnia and osteoporosis, and a new perspective on safe drug choice. J. Menopausal Med. 24, 143–149. 10.6118/jmm.2018.24.3.143 30671405 PMC6336562

[B102] You Y. Wang D. Yuan J. Hua Q. (2022). 43 cases of perimenopausal insomnia treated with Zigui Tiaogeng Anshen Decoction combined with western medicine. Chin. J. Traditional Med. Sci. Technol. 29, 330–332.

[B103] Zeng C. Xie Y. Dai C. Yuan Z. (2022). Double-blind randomized controlled study on jiaotai pills for insomnia of NonInteraction between the heart and the kidney type in perimenopausal period in clinic. New Chin. Med. 54, 41–44.

[B104] Zhang A. Xue C. Fong H. (2011). Chapter 22: integration of herbal medicine into evidence-based clinical practice. Herb. Med. Biomol. Clin. Aspects, 1–15.

[B105] Zhang C. Guo J. Zheng W. (2021). 40 cases of perimenopausal insomnia treated with self-prepared Bushen Shugan Ningxin Decoction. CJGMCM 36, 3291–3293.

[B106] Zhang W. Huai Y. Miao Z. Qian A. Wang Y. (2019). Systems pharmacology for investigation of the mechanisms of action of traditional Chinese medicine in drug discovery. Front. Pharmacol. 10, 743. 10.3389/fphar.2019.00743 31379563 PMC6657703

[B107] Zhang W.-H. Liu Q.-L. Zhang Z.-X. Huang T. Cai Z.-X. (2012). Randomized control research on theory of Yunqi in differentiating and treating menopausal insomnia of liver-kidney deficiency pattern. Sh. J. TCM 46, 51–53.

[B108] Zhang Y.-M. Chen L. (2009). Clinical observation of “guyuan ningshen decoction” on perimenopausal insomnia of heart and kidney disharmony. Sh. J. TCM 43, 39–40.

[B109] Zhang Y.-M. Huang J.-S. Zhang M. Zhang Y.-F. Gao Y.-D. Deng J.-J. (2020). Clinical study on Songyu Yinxu Recipe in the treatment of perimenopausal insomnia syndrome of kidney deficiency and liver stagnation. China J. Traditional Chin. Med. Pharm. 35, 2102–2105.

[B110] Zhao F. Y. Fu Q. Q. Kennedy G. A. Conduit R. Wu W. Z. Zhang W. J. (2021). Comparative utility of acupuncture and western medication in the management of perimenopausal insomnia: a systematic review and meta-analysis. Evid. Based Complement. Altern. Med. 2021, 5566742. 10.1155/2021/5566742 PMC8093060 33986818

[B111] Zhao G.-P. Zhang H.-L. Xue C. Feng W.-J. (2017). Efficacy observation of semen ziziphi spinosae decoction on climacteric insomnia. Shanxi J TCM 33, 20–21 + 31.

[B112] Zhao X. (2018). Analysis of the effectiveness of modified Suanzaoren Decoction in the treatment of menopausal patients with insomnia. Psychol. Dr. 24, 24–25.

[B113] Zheng M. (2020). Observation on the efficacy and safety of huanglian ejiao decoction combined with ganmai dazao decoction in the treatment of menopausal insomnia. Gems Health 212.

[B114] Zheng Y. (2016). Observation on the efficacy of Guizhi plus Longgu Oyster Decoction in the treatment of perimenopausal patients with anxiety insomnia disorder. Chin. Prim. Health Care 30, 59–60.

[B115] Zhou Q. H. Zhou X. L. Xu M. B. Jin T. Y. Rong P. Q. Zheng G. Q. (2018). Suanzaoren formulae for insomnia: updated clinical evidence and possible mechanisms. Front. Pharmacol. 9, 76. 10.3389/fphar.2018.00076 29479317 PMC5811769

[B116] Zhu J. Wang Y. (2020). Clinical study on Buxin Xiaoyao yin in treating perimenopausal insomnia. J. Basic Chin. Med. 26, 1121–1122 + 1199.

[B117] Zhu X. Liew Y. Liu Z. L. (2016). Chinese herbal medicine for menopausal symptoms. Cochrane Database Syst. Rev. 3, Cd009023. 10.1002/14651858.CD009023.pub2 26976671 PMC4951187

[B118] Zhu X. Tao M. Hu H. Gao J. Chen J. Lu T. (2022). The efficacy and safety of zaoren anshen capsule in combination with zolpidem for insomnia: a multicentre, randomized, double-blinded, placebo-controlled trial. Evid. Based Complement. Altern. Med. 2022, 5867523. 10.1155/2022/5867523 PMC9711951 36467553

