# Supplementary material for: Effectiveness and safety of East Asian herbal medicine for menopausal insomnia: a systematic review and meta-analysis
Source: Front Pharmacol. 2024 Aug 8;15:1414700. doi: 10.3389/fphar.2024.1414700 (PMC11338872; doi:10.3389/fphar.2024.1414700)

**Supplement 1. Search terms used in each database and results**

**Medline via PubMed**

|  | Searches | Results |
| --- | --- | --- |
| #1 | Climacteric[MH] OR Menopause[MH] OR Postmenopause[MH] OR Perimenopause[MH] OR postmenopaus*[TIAB] OR post‐menopaus*[TIAB] OR "post menopaus*"[TIAB] OR menopaus*[TIAB] OR perimenopaus*[TIAB] OR peri‐menopaus*[TIAB] OR "peri menopaus*"[TIAB] OR climacter*[TIAB] | 122,314 |
| #2 | "Sleep Wake Disorders"[MH] OR Sleep[MH] OR sleep*[TIAB] OR insomnia*[TIAB] OR dyssomn*[TIAB] OR awake*[TIAB] OR wake*[TIAB] OR waking[TIAB] OR awaking[TIAB] | 323,136 |
| #3 | “Plants, Medicinal”[MH] OR “Drugs, Chinese Herbal”[MH] OR “Medicine, Chinese Traditional”[MH] OR “Medicine, Kampo”[MH] OR “Medicine, Korean Traditional”[MH] OR “Herbal Medicine”[MH] OR “traditional Korean medicine”[TIAB] OR “traditional Chinese medicine”[TIAB] OR “traditional oriental medicine”[TIAB] OR “Kampo medicine”[TIAB] OR “medicinal plants”[TIAB] OR herb*[TIAB] OR prescription[TIAB] OR decoction[TIAB] OR tang[TIAB] OR capsule[TIAB] OR powder[TIAB] OR botanic*[TIAB] | 509,482 |
| #4 | “randomized controlled trial”[PT] OR “controlled clinical trial”[PT] OR randomized[TIAB] OR placebo[TIAB] OR “drug therapy”[SH] OR randomly[TIAB] OR trial[TIAB] OR groups[TIAB] | 5,787,240 |
| #5 | animals[MH] NOT humans[MH] | 5,137,847 |
| #6 | #1 AND #2 AND #3 AND #4 NOT #5 | **120** |

**Embase via Elsevier**

|  | Searches | Results |
| --- | --- | --- |
| #1 | 'menopausal syndrome'/exp OR 'menopause and climacterium'/exp OR postmenopaus*:ab,ti OR post‐menopaus*:ab,ti OR 'post menopaus*':ab,ti OR menopaus*:ab,ti OR perimenopaus*:ab,ti OR peri‐menopaus*:ab,ti OR 'peri menopaus*':ab,ti OR climacter*:ab,ti | 198,100 |
| #2 | sleep/exp OR 'sleep disorder'/exp OR insomnia/exp OR sleep*:ab,ti OR insomnia*:ab,ti OR dyssomn*:ab,ti OR awake*:ab,ti OR wake*:ab,ti OR waking:ab,ti OR awaking:ab,ti | 602,898 |
| #3 | ‘medicinal plant’/exp OR ‘medicinal plant’:ab,ti OR ‘herbaceous agent’/exp OR ‘herbaceous agent’:ab,ti OR ‘chinese medicine’/exp OR ‘chinese medicine’:ab,ti OR ‘kampo medicine’/exp OR ‘kampo medicine’:ab,ti OR ‘kampo medicine (drug)’/exp OR ‘kampo medicine (drug)’:ab,ti OR ‘korean medicine’/exp OR ‘korean medicine’:ab,ti OR ‘herbal medicine’/exp OR ‘herbal medicine’:ab,ti OR ‘oriental medicine’/exp OR ‘oriental medicine’:ab,ti OR herb/exp OR herb*:ab,ti OR prescription:ab,ti OR decoction:ab,ti OR tang:ab,ti OR capsule:ab,ti OR powder:ab,ti OR botanic*:ab,ti | 871,753 |
| #4 | 'crossover procedure':de OR 'double-blind procedure':de OR 'randomized controlled trial':de OR 'single-blind procedure':de OR (random* OR factorial* OR crossover* OR cross NEXT/1 over* OR placebo* OR doubl* NEAR/1 blind* OR singl* NEAR/1 blind* OR assign* OR allocat* OR volunteer*):de,ab,ti | 3,167,551 |
| #5 | #1 AND #2 AND #3 AND #4 | **307** |

**CENTRAL**

|  | Searches | Results |
| --- | --- | --- |
| #1 | MeSH descriptor: [Climacteric] explode all trees | 9,828 |
| #2 | MeSH descriptor: [Menopause] explode all trees | 9,585 |
| #3 | MeSH descriptor: [Postmenopause] explode all trees | 6,459 |
| #4 | MeSH descriptor: [Perimenopause] explode all trees | 195 |
| #5 | (postmenopaus* OR post‐menopaus* OR menopaus* OR perimenopaus* OR peri‐menopaus* OR climacter*):ti,ab,kw | 30,963 |
| #6 | #1 OR #2 OR #3 OR #4 OR #5 | 31,699 |
| #7 | MeSH descriptor: [Sleep Wake Disorders] explode all trees | 10,999 |
| #8 | MeSH descriptor: [Sleep] explode all trees | 9,474 |
| #9 | (sleep* OR insomnia* OR dyssomn* OR awake* OR wake* OR waking OR awaking):ti,ab,kw | 66,269 |
| #10 | #7 OR #8 OR #9 | 66,669 |
| #11 | MeSH descriptor: [Plants, Medicinal] explode all trees | 1,040 |
| #12 | MeSH descriptor: [Drugs, Chinese Herbal] explode all trees | 4,185 |
| #13 | MeSH descriptor: [Medicine, Chinese Traditional] explode all trees | 1,554 |
| #14 | MeSH descriptor: [Medicine, Kampo] explode all trees | 58 |
| #15 | MeSH descriptor: [Medicine, Korean Traditional] explode all trees | 41 |
| #16 | MeSH descriptor: [Herbal Medicine] explode all trees | 243 |
| #17 | (“traditional Korean medicine” OR “traditional Chinese medicine” OR “traditional oriental medicine” OR “Kampo medicine” OR “medicinal plants” OR herb* OR prescription OR decoction OR tang OR capsule OR powder OR botanic*):ti,ab,kw | 76,711 |
| #18 | #11 OR #12 OR #13 OR #14 OR #15 OR #16 OR #17 | 77,941 |
| #19 | (#6 AND #10 AND #18) in Trials | **258** |

**AMED via EBSCO**

|  | Searches | Results |
| --- | --- | --- |
| #1 | SU climacteric OR SU menopause OR SU postmenopause OR SU perimenopause OR TX postmenopaus* OR TX post‐menopaus* OR TX "post menopaus*" OR TX menopaus* OR TX perimenopaus* OR TX peri‐menopaus* OR TX "peri menopaus*" OR TX climacter* | 1,293 |
| #2 | SU "Sleep Wake Disorders" OR SU sleep OR SU insomnia OR TX sleep* OR TX insomnia* OR TX dyssomn* OR TX awake* OR TX wake* OR TX waking OR TX awaking | 4,593 |
| #3 | SU “Herbal Medicine” OR SU “Herbal Drugs” OR SU herbs OR SU “Herbal Preparations” OR SU herbalism OR SU “Traditional Medicine Korean” OR SU “Traditional Medicine Chinese” OR SU “Kampo Medicine” OR SU Kampo OR SU “Medicine Kampo” OR SU “Traditional Medicine Oriental” OR TX “herbal medicine” OR TX “medicinal plants” OR TX herb* OR TX “traditional Chinese medicine” OR TX “traditional Korean medicine” OR TX Kampo OR TX “Oriental medicine*” OR TX prescription OR TX decoction OR TX tang OR TX capsule OR TX powder OR TX botanic* | 32,154 |
| #4 | #1 AND #2 AND #3 | **26** |

**OASIS**

|  | Searches | Results |
| --- | --- | --- |
| #1 | (갱년기\|폐경) (불면\|수면) (한약\|약초\|본초\|탕\|환\|산) | **0** |

**RISS**

|  | Searches | Results |
| --- | --- | --- |
| #1 | (갱년기\|폐경) (불면\|수면) (한약\|약초\|본초\|탕\|환\|산) | **19** |

**KMbase**

|  | Searches | Results |
| --- | --- | --- |
| #1 | ([ALL=갱년기] OR [ALL=폐경]) | 2,418 |
| #2 | ([ALL=불면] OR [ALL=수면]) | 3,151 |
| #3 | ((((([ALL=한약] OR [ALL=약초]) OR [ALL=본초]) OR [ALL=탕]) OR [ALL=환]) OR [ALL=산]) | 118,526 |
| #4 | #1 AND #2 AND #3 | **0** |

**ScienceON**

|  | Searches | Results |
| --- | --- | --- |
| #1 | (갱년기\|폐경) (불면\|수면) (한약\|약초\|본초\|탕\|환\|산) | **16** |

**CNKI**

|  | Searches | Results |
| --- | --- | --- |
| #1 | (SU=‘更年期’+’绝经’) AND (SU=‘失眠’+’不寐’+’不眠’+’不睡’+’不得眠’+’不得卧’+’睡眠’) AND (SU=‘中医药’+’中医’+’中西医结合’+’中药’+’本草’+’汤’+’丸’+’散’+’饮’+’颗粒’+’胶囊’) | **599** |

**Wanfang data**

|  | Searches | Results |
| --- | --- | --- |
| #1 | (主题:更年期 OR 主题:绝经) AND (主题:失眠 OR 主题:不寐 OR 主题:不眠 OR 主题:不睡 OR 主题:不得眠 OR 主题:不得卧 OR 主题:睡眠) AND (主题:中医药 OR 主题:中医 OR 主题:中西医结合 OR 主题:中药 OR 主题:本草 OR 主题:汤 OR 主题:丸 OR 主题:散 OR 主题:饮 OR 主题:颗粒 OR 主题:胶囊) | **3014** |

**Supplement 2.** **Excluded studies after full-text review**

1) not-RCTs (n = 53)

- 耿鑫婷 (2018). 调冲安神汤治疗更年期综合征伴失眠临床研究. *养生保健指南* (19)**,** 104. doi: 10.3969/j.issn.1006-6845.2018.19.093.
- 曲秀芬, and 李莹 (2013). "滋肾安神汤治疗女性围绝经期心肾不交型失眠", in: *第十三次全国中医妇科学术大会论文集.* (贵阳).
- 邱雪凤, and 徐慧军 (2017). 浅析加味酸枣仁汤治疗围绝经期失眠症. *中国民族民间医药* 26(17)**,** 62-64.
- 金敏娟 (2009). 中西医结合治疗更年期失眠症55例. *浙江中医杂志* 44(02)**,** 125.
- 董敏阶, and 高建忠 (2007). 酸枣安神汤治疗女性围绝经期失眠. *山东中医杂志* (12)**,** 829.
- 杜鹃, and 王利平 (2021). 桂枝加龙骨牡蛎汤合黄连阿胶汤加味治疗围绝经期失眠. *实用中西医结合临床* 21(11)**,** 38-39.
- 罗彩凤, 魏清琳, 王正婷, 边小平, 马泉, and 罗彩芸 (2020). 中医综合疗法治疗围绝经期失眠症. *中医药临床杂志* 32(10)**,** 1986-1990.
- 梁晓星, 丁凤, and 刘秀芬 (2005). 加味三才饮治疗围绝经期不寐60例. *河北中医* (09)**,** 650.
- 鲁娜 (2018). 中西医结合治疗更年期失眠34例疗效观察. *中国民族民间医药* 27(12)**,** 112-114.
- 刘建胜 (2020). 六味地黄汤加味治疗更年期阴虚火旺型失眠66例疗效观察. *中国保健营养* 30(26)**,** 362-363.
- 刘东晖 (2018). 温胆汤加减治疗痰热型围绝经期失眠临床观察. *临床医药文献电子杂志* 5(24)**,** 169.
- 李竞 (2013). “疏肝安神汤”治疗女性更年期不寐51例. *江苏中医药* 45(07)**,** 77.
- 李竞 (2015). "疏肝安神汤治疗女性更年期不寐51例", in: *2015年浙江省心身医学学术年会论文汇编.* (中国浙江金华).
- 李志军, and 海日汗 (2020). 女珍颗粒联合佐匹克隆治疗更年期失眠症的临床研究. *药物评价研究* 43(01)**,** 120-124.
- 李佩林, 袁雯, 魏丹, and 徐慧军 (2023). 基于开阖枢选用柴胡桂枝干姜汤治疗围绝经期失眠. *新中医* 55(9)**,** 208-212. doi: 10.13457/j.cnki.jncm.2023.09.043.
- 林艳 (2009). 步长稳心颗粒与阿普唑仑联合治疗更年期心悸失眠36例. *陕西中医* 30(10)**,** 1365-1366.
- 马堃, 陈燕霞, and 董美玲 (2017). 从肾虚血瘀与心肾不交论治围绝经期睡眠障碍. *中国中药杂志* 42(23)**,** 4455-4458.
- 马湖蕊, and 贾云 (2012). 中医药情志干预治疗女性更年期失眠症. *医药前沿* 2(1)**,** 337-338. doi: 10.3969/j.issn.2095-1752.2012.01.428.
- 蒙艳村, 李善霞, 黄孆蝉, and 崔芳榕 (2022). 基于"调肝肾,和阴阳"应用滋水清肝饮论治围绝经期失眠. *中国民间疗法* 30(17)**,** 7-10. doi: 10.19621/j.cnki.11-3555/r.2022.1703.
- 史丽萍 (2016). 当归六黄汤加减治疗女性更年期失眠的临床疗效观察. *健康之路* (8).
- 谢杭珍, 林光斌, and 郑婷婷 (2017). 归脾丸在围绝经期失眠症治疗中的临床应用. *临床合理用药杂志* 10(25)**,** 99-100.
- 宋艳杰, 邢佳丽, and 宋元元 (2009). 桂枝加龙骨牡蛎汤合黄连阿胶汤加味治疗围绝经期失眠36例. *河北中医* 31(02)**,** 222-223.
- 杨小良, and 王展 (2018). 探讨酸枣仁汤合甘麦大枣汤加减治疗更年期不寐的临床效果. *家庭医药.就医选药* (06)**,** 41.
- 杨荣波 (2017). 柏子养心汤加减治疗中老年围绝经期失眠症的临床研究. *云南中医中药杂志* 38(09)**,** 29-31.
- 杨永年 (1998). 丹栀逍遥散加减治疗更年期失眠72例. *江苏中医* (01)**,** 22.
- 闫秀萍 (2003). 自拟滋阴降火汤治疗更年期不寐疗效观察. *山西职工医学院学报* (03)**,** 25.
- 吴茜 (2002). 自拟妇复宁汤治疗更年期妇女顽固性失眠60例. *中国中医药科技* (02)**,** 89.
- 王钢, 沈燕, and 董滟 (2011). 小议丹栀逍遥散合百合地黄汤在治疗更年期失眠中应用. *医药前沿* 1(23)**,** 360-361. doi: 10.3969/j.issn.2095-1752.2011.23.313.
- 王新 (2012). 综合疗法治疗妇女更年期失眠的探索. *中国卫生产业* (25)**,** 114.
- 王青 (2013). 自拟更年安神汤治疗围绝经期失眠临床观察. *中医临床研究* 5(21)**,** 68-69.
- 王春浩 (2019). 为更年期综合征所致失眠患者使用同仁安神丸联合参芪五味子片进行治疗的效果探究. *当代医药论丛* 17(13)**,** 204-205.
- 于斌 (2004). 百合清心调志汤治疗妇女更年期虚烦失眠证32例. *江苏中医药* (07)**,** 31.
- 于晓妹 (2006). 更年康胶囊治疗女性更年期失眠症62例疗效观察. *中华临床医学杂志* 007(3)**,** 58-59.
- 韦坚 (2012). 滋阴安更汤治疗更年期失眠症疗效观察. *医学新知·综合版* (1).
- 韦婉 (2013). 温胆汤加减治疗痰瘀阻滞型更年期失眠. *吉林中医药* 33(07)**,** 675-677.
- 章敬芳, and 黄伟明 (2018). 四逆散加味治疗更年期失眠的临床观察. *光明中医* 33(14)**,** 2037-2039.
- 张丽娟 (2005). 女神散对更年期失眠的疗效. *国外医学(中医中药分册)* (03)**,** 172.
- 张旭东, 张淑慧, and 张彬 (2008). 复方刺五加糖浆治疗心脾两虚型更年期妇女不寐的研究. *现代中西医结合杂志* 17(35)**,** 5413-5414,5430. doi: 10.3969/j.issn.1008-8849.2008.35.001.
- 张宁, 刘佳, 刘凡琪, 陈娟, 祁宇, 刘爱琪, et al. (2020). 围绝经期失眠论治以肝肾为先. *环球中医药* 13(7)**,** 1250-1253. doi: 10.3969/j.issn.1674-1749.2020.07.030.
- 庄昌辉, 陈建胜, and 毛丹丹 (2015). 乌灵胶囊治疗围绝经期高血压伴睡眠障碍68例. *浙江中西医结合杂志* 25(12)**,** 1136-1138.
- 张秋明, and 张雪冰 (2019). 甘麦大枣汤合桂枝龙骨牡蛎汤治疗围绝经期失眠疗效观察. *中西医结合心血管病电子杂志* 7(10)**,** 150.
- 全香花, 彭小菊, 韩建香, 于振英, and 汪川 (2010). 安神养心免煎颗粒治疗更年期妇女不寐37例. *新中医* 42(04)**,** 43-44.
- 占翠红, 梁永凯, 马俊, and 彭钊 (2010). 中西医结合治疗更年期失眠的临床观察. *黑龙江医药* 23(06)**,** 994-995.
- 赵树理 (2015). 黄连阿胶汤加减治疗更年期不寐100例临床观察. *内蒙古中医药* 34(04)**,** 30.
- 左金玲, 田慧萍, 武永强, and 朱崇华 (2018). 甘麦大枣汤合百合地黄龙牡汤治疗围绝经期失眠临床观察. *实用中医药杂志* 34(04)**,** 424-425.
- 陈乐君, and 戴建国 (2014). 从肝肾阴虚论治围绝经期失眠症. *河南中医* 34(0)**,** 360-361.
- 沈晓英, 陈菊华, and 张力 (2021). 柴胡龙骨牡蛎汤加减治疗肝郁型更年期睡眠障碍临床观察. *光明中医* 36(06)**,** 878-880.
- 冯立新 (2002). 逍遥散加味治疗女性更年期失眠35例. *中国中医基础医学杂志* (11)**,** 71.
- 冯雅莉 (2006). 桂枝加龙骨牡蛎汤治疗更年期失眠18例. *光明中医* (08)**,** 45-46.
- 郝振华, and 王银燕 (2019). 酸枣仁汤治疗更年期失眠患者的疗效研究. *世界最新医学信息文摘* 19(A5)**,** 192+196.
- 胡连根, 李国岩, and 秦琬玲 (2007). 逍遥散合百合地黄汤加减治疗女性更年期失眠19例. *江西中医药* (06)**,** 52.
- 黄进学, 赵改红, 郭雪松, 杨文媛, 于小刚, 解欢欢, et al. (2022). 从肾虚肝郁论治更年期失眠. *中医临床研究* 14(10)**,** 88-90. doi: 10.3969/j.issn.1674-7860.2022.10.026.
- 黄晓莺 (2007). 归脾合剂合左归丸治疗妇女更年期失眠症33例. *中成药* (03)**,** 483-484.

2) without appropriate randomization method (n = 134)

- Chen, J., Weng, Q., Liang, Y., and Wu, Y. (2022). Effect of Using Modified Ganmai Dazao Decoction and Erzhi Pill Combined with Estrogen and Progesterone in the Treatment of Menopausal Syndrome and Its Influence on Sleep Quality. *Journal of Sichuan of Traditional Chinese Medicine* 40(07)**,** 156-159.
- Jokar, A., Kargosha, A., Akarzadeh, M., Asadi, N., and Setoudeh, Z. (2016). Comparing the influence of relaxation training and consumption of valerian on insomnia of menopause women: a randomized clinical trial. *African journal of traditional, complementary and alternative medicines* 13(1)**,** 40‐44. doi: 10.4314/ajtcam.v13i1.6.
- Yang, S., Liang, Y., and Xheng, J. (2020). Analysis of the Effect of Modified Ganmai Dazao Decoction on the Improvement of Sleep Quality of Female Menopause Patients. *World Journal of Sleep Medicine* 7(11)**,** 1920-1921.
- Zheng, X. (2020). Effects of Bushenlemian Decoction Combined with Hormone Replacement Therapy on Hormone Levels and Sleep Quality in Peri-menopausal Insomnia Patients. *World Journal of Sleep Medicine* 7(10)**,** 1776-1777.
- 康珺楠 (2015). 逍遥散加味治疗更年期不寐症的疗效分析. *中国初级卫生保健* 29(03)**,** 93-94.
- 居跃君 (2011). 从肝肾论治围绝经期失眠症临床观察. *光明中医* 26(7)**,** 1403-1404. doi: 10.3969/j.issn.1003-8914.2011.07.057.
- 古衍, 姚吉龙, and 姜向坤 (2007). 补肾安神方治疗围绝经期失眠(阴虚火旺型)32例疗效观察. *新中医* 39(9)**,** 28-29. doi: 10.3969/j.issn.0256-7415.2007.09.017.
- 曲桂红, and 孙润蛟 (2018). 中西医结合疗法治疗更年期合并睡眠障碍临床研究. *世界睡眠医学杂志* 5(01)**,** 62-65.
- 孔月晴, and 胡建鹏 (2011). 加味抑肝散治疗更年期妇女失眠42例临床观察. *中国妇幼保健* 26(34)**,** 5380-5382.
- 霍群菲 (2019). 围绝经期失眠症应用滋水清肝饮治疗的有效性分析. *健康必读* (15)**,** 70.
- 郭芳 (2010). 中药结合生物反馈仪治疗更年期失眠87例. *光明中医* 25(11)**,** 2014-2015.
- 郭安忆, 鲁玙, and 张瑜 (2022). 固本安神汤治疗心肾不交型更年期失眠43例. *浙江中医杂志* 57(08)**,** 602.
- 邱丽, 管雁丞, and 刘玉 (2015). 归肾两地汤加减治疗肝肾阴虚型绝经前后诸证疗效观察. *新中医* 47(7)**,** 191-193. doi: 10.13457/j.cnki.jncm.2015.07.085.
- 仇燕飞 (2015). 黄连阿胶汤合甘麦大枣汤治疗更年期失眠50例. *河南中医* 35(08)**,** 1764-1766.
- 丘翠玲, and 易伟剑 (2011). 综合疗法治疗围绝经期妇女失眠症40例. *云南中医中药杂志* 32(4)**,** 25-25. doi: 10.3969/j.issn.1007-2349.2011.04.014.
- 瞿夏凡, and 胡国恒 (2022). 中西医结合治疗肝郁化火型围绝经期失眠临床观察. *中国中医药现代远程教育* 20(21)**,** 128-130.
- 金赵爽 (2019). 加味酸枣仁汤合温胆汤治疗女性更年期失眠疗效分析. *家庭医药.就医选药* (01)**,** 149.
- 梁艳, and 郑丽娟 (2022). 百合地黄汤联合常规西医治疗女性围绝经期失眠的疗效观察. *现代医药卫生* 38(14)**,** 2451-2454. doi: 10.3969/j.issn.1009-5519.2022.14.025.
- 吕梦亮, 彭思菡, and 何淑娴 (2017). 桂枝甘草龙骨牡蛎汤加减治疗围绝经失眠的临床分析. *光明中医* 32(09)**,** 1262-1263.
- 吕俊廷, 石洲宝, 高娜, and 陈长浩 (2015). 甘麦大枣汤加减对围绝经期女性睡眠障碍的临床研究. *光明中医* 30(06)**,** 1229-1230.
- 卢英翔 (2003). 加味杞菊地黄汤治疗更年期失眠症40例. *国医论坛* 18(5)**,** 23. doi: 10.3969/j.issn.1002-1078.2003.05.019.
- 赖瑜梅, 徐守权, 孙玉霞, 黄丽华, 张杰果, 赖炳丽, et al. (2017). 归脾汤加麦冬治疗围绝经期妇女失眠临床观察. *陕西中医* 38(7)**,** 831-832. doi: 10.3969/j.issn.1000-7369.2017.07.008.
- 刘杰 (2018). 百乐眠胶囊联合莉芙敏治疗伴失眠症更年期综合征的临床观察. *中国医药指南* 16(14)**,** 192.
- 刘娜娜, 王赛, and 康斐 (2016). 调冲安神汤治疗更年期综合征伴失眠临床研究. *河南中医* 36(10)**,** 1775-1777.
- 刘臣, and 翟坤光 (2009). 鳖龙汤治疗围绝经期失眠30例. *中国中医药科技* 16(02)**,** 89.
- 刘蕊, 曲丹, and 刘欢 (2015). 挹神汤加减治疗女性围绝经期失眠症36例的临床研究. *中国医药导刊* 17(01)**,** 52-53.
- 刘玉如 (2019). 引火汤辨证治疗女性更年期不寐的临床疗效. *名医* (05)**,** 275.
- 刘春霞 (2016). 酸枣仁汤合左归丸治疗妇女更年期失眠的效果探究. *世界中医药* 0(0)**,** 1753.
- 刘忠文, and 刘迎辉 (2010). 中西医结合治疗女性更年期失眠疗效观察. *中国实用医药* 5(28)**,** 168-169.
- 李健, and 魏格玲 (2016). 滋肾调肝法对围绝经期失眠的近期和远期疗效观察. *四川中医* 34(6)**,** 175-177.
- 李光跃, 彭芳, and 崔淑华 (2015). 参松养心胶囊治疗围绝经期妇女失眠42例疗效观察. *河北中医* 37(04)**,** 584-586.
- 李克建 (2017). 柏子养心汤加减治疗中老年围绝经期失眠症. *吉林中医药* 37(12)**,** 1219-1222.
- 李梦 (2011). 六味地黄丸合交泰丸治疗更年期失眠症40例. *浙江中医杂志* 46(06)**,** 408.
- 李宝玲, and 戴红 (2007). 解郁宁神汤治疗女性更年期失眠临床观察. *中国民间疗法* (10)**,** 30-31.
- 李秀华 (2021). 酸枣仁汤加谷维素治疗更年期失眠的效果研究. *世界睡眠医学杂志* 8(8)**,** 1353-1354. doi: 10.3969/j.issn.2095-7130.2021.08.022.
- 李晨晨, and 孙立军 (2015). 更年期方治疗围绝经期心肾不交型失眠50例总结. *湖南中医杂志* 31(11)**,** 79-80.
- 李双 (2019). 酸枣仁汤合甘麦大枣汤治疗更年期失眠伴焦躁患者效果观察. *实用中西医结合临床* 19(09)**,** 37-39.
- 李阳 (2021). 滋心养肾汤治疗围绝经期失眠症心肾不交证的疗效观察. *现代诊断与治疗* 32(19)**,** 3044-3046.
- 李玉舸 (2022). 柴胡龙骨牡蛎汤加减治疗围绝经期失眠的临床研究. *医学食疗与健康* 20(15)**,** 33-35+193.
- 李天雨 (2018). 加味酸枣仁汤合温胆汤治疗女性更年期失眠疗效观察. *临床医药文献电子杂志* 5(24)**,** 175.
- 李清媛, 冀德才, and 乔宇 (2013). 酸枣仁汤加减治疗更年期失眠症疗效观察. *中国实用医药* 8(7)**,** 154-155. doi: 10.3969/j.issn.1673-7555.2013.07.111.
- 李霞, 张俊, and 方晏平 (2016). 知柏地黄汤加味联合西药治疗围绝经期失眠肾阴虚证42例. *河南中医* 36(8)**,** 1422-1424. doi: 10.16367/j.issn.1003-5028.2016.08.0579.
- 蔺玉琴 (2021). 天王补心丹加减治疗心肾不交型围绝经期失眠25例疗效观察. *中医临床研究* 13(10)**,** 91-94. doi: 10.3969/j.issn.1674-7860.2021.10.031.
- 马海燕 (2015). 自拟解郁安神汤治疗围绝经期失眠症70例临床观察. *浙江中医杂志* 50(02)**,** 114.
- 马湖蕊, 熊彦辉, and 李成能 (2014). 舒乐安定配合芪参胶囊治疗女性更年期失眠症. *药物与人* 27(0)**,** 36.
- 麦秀军 (2012). 黄连阿胶汤治疗更年期失眠的临床体会. *现代诊断与治疗* 23(11)**,** 1872-1873.
- 闵肖岚 (2007). 更年安睡饮治疗围绝经期妇女失眠45例. *四川中医* (03)**,** 72-74.
- 房晓曼, and 刘瑞 (2022). 芬吗通联合坤泰胶囊对更年期综合征伴失眠患者的治疗效果及对其性激素水平和心理状态的影响. *首都食品与医药* 29(4)**,** 70-72. doi: 10.3969/j.issn.1005-8257.2022.04.033.
- 边娜, and 闫璇 (2023). 合欢解郁汤联合盐酸曲唑酮治疗更年期睡眠障碍的效果分析. *贵州医药* 47(05)**,** 766-767.
- 史亮亮 (2023). 柴胡加龙骨牡蛎汤加减治疗肝气郁滞型更年期失眠的疗效分析. *长寿* (3)**,** 97-99.
- 司艳君 (2021). 二仙汤合甘麦大枣汤加减治疗女性更年期失眠的疗效. *特别健康* (35)**,** 63-64.
- 司静文, 石子璇, 赵娇, and 王南丁 (2014). 百乐眠胶囊治疗女性更年期失眠症的疗效观察. *陕西中医* 35(01)**,** 46-47.
- 谢雅贞 (2022). 柴胡桂枝龙骨牡蛎汤加减治疗围绝经期肝郁型失眠30例临床观察. *中国民族民间医药* 31(24)**,** 114-118.
- 巢超君 (2017). 解郁安神汤治疗女性更年期失眠症的效果观察. *常州实用医学* 33(2)**,** 81-83.
- 孙亚平, 张建春, 王彩娟, 刘迎春, 张耕源, and 谢娜 (2018). 疏肝解郁法对肝郁型围绝经期失眠女性血清IL-6、NPY及负性情绪的影响. *世界中医药* 13(2)**,** 340-343,347. doi: 10.3969/j.issn.1673-7202.2018.02.022.
- 孙辉 (Year). "中药汤剂合并黛力新治疗女性围绝经期失眠的对照研究", in: *中国中西医结合学会精神疾病专业委员会第15届全国学术会议暨第2届京津冀中西医结合精神疾病学年会暨全国名老中医药专家王彦恒临床经验学习班*), 2.
- 宋立英, 曹腊梅, 殷欣, and 闫红梅 (2019). 桂枝甘草龙骨牡蛎汤加减治疗围绝经失眠的临床价值体会. *现代养生* (14)**,** 125-126.
- 荣斐 (2020). 补肾乐眠汤在围绝经期妇女顽固性失眠治疗中的效果观察. *中外女性健康研究* (05)**,** 111-112.
- 申斐, 韩冠先, and 郭水洁 (2018). 柴胡龙骨牡蛎汤治疗女性更年期失眠的临床疗效. *中国保健营养* 28(35)**,** 135-136. doi: 10.3969/j.issn.1004-7484.2018.35.161.
- 辛海艳, and 王燕龙 (2012). 加味甘麦大枣汤治疗更年期妇女失眠的临床体会. *中国保健营养* 22(12)**,** 2290-2291.
- 杨佳澎 (2016). 柏子养心汤加味治疗围绝经期心脾两虚型失眠症临床观察. *河北中医* 38(5)**,** 737-740. doi: 10.3969/j.issn.1002-2619.2016.05.027.
- 杨国红 (2015). 酸枣仁汤剂治疗女性更年期失眠的疗效观察. *医学美学美容（中旬刊）* (4)**,** 313-314.
- 杨雪玲 (2020). 更年期失眠患者应用桂枝甘草龙骨牡蛎汤加减治疗的效果观察. *家庭生活指南* (02)**,** 128.
- 杨衍涛 (2017). 坤宁益寐汤治疗女性更年期不寐76例. *西部中医药* 30(08)**,** 87-88.
- 杨芍 (2013). 安度更年丸治疗围绝经期失眠50例. *光明中医* 28(09)**,** 1855-1856.
- 杨茜 (2017). 中药安神丸治疗女性更年期失眠的临床效果观察. *内蒙古中医药* 36(10)**,** 34.
- 吴西志 (2015). 补肾安神汤治疗围绝经期妇女顽固性失眠症45例. *河南中医* 35(09)**,** 2142-2144.
- 王剑虹 (2002). 自拟水火既济汤治疗阴虚火旺型更年期不寐30例疗效观察. *北京中医* (05)**,** 283-284.
- 汪桂花 (2020). 调冲安神汤在更年期综合征伴失眠患者治疗中的临床研究. *中外女性健康研究* (12)**,** 109+114.
- 王琦 (2023). 甘麦大枣汤治疗肾虚肝郁型围绝经期失眠患者的疗效观察. *中国冶金工业医学杂志* 40(01)**,** 73-74.
- 王丽, 王彩娟, 芮绵, 侯月平, 宋立英, and 曹腊梅 (2017). 清心镇肝汤对围绝经期失眠血清5-HT及5-HIAA的影响. *陕西中医* 38(03)**,** 326-327.
- 汪立峰 (2011). 中西医结合治疗更年期妇女失眠症疗效分析. *医学信息（上旬刊）* 24(11)**,** 326-327. doi: 10.3969/j.issn.1006-1959.2011.11.420.
- 王普京 (2006). 柴胡加龙骨牡蛎汤加减治疗更年期失眠的疗效分析. *中国中医基础医学杂志* (05)**,** 369+382.
- 王雅红, 霍晓燕, 刘桂英, 张春香, and 曾小芳 (2018). 补肾清心汤治疗围绝经期睡眠障碍临床研究. *中国药业* 27(11)**,** 58-60.
- 王旭玲, 张晓昀, 原凡惠, and 宋彩红 (2012). 酸枣仁汤合甘麦大枣汤加减治疗更年期不寐. *吉林中医药* 32(07)**,** 701.
- 王晓萍 (2021). 补肾乐眠汤治疗围绝经期妇女顽固性失眠患者的临床效果. *中国医药指南* 19(02)**,** 139-140.
- 姚奏英, 陈慧, and 朱丽萍 (2018). 滋肾解郁宁心方加减治疗围绝经期失眠临床观察. *中成药* 40(1)**,** 240-242. doi: 10.3969/j.issn.1001-1528.2018.01.054.
- 姚辉菊 (2016). 化裁交泰丸治疗心肾不交型更年期失眠疗效观察. *实用中医药杂志* 32(07)**,** 657-658.
- 牛根山 (2011). 稳心颗粒治疗女性更年期心悸、失眠症的疗效观察. *实用心脑肺血管病杂志* 19(10)**,** 1763.
- 于首元, and 于兆安 (2009). 中医治疗围绝经期妇女失眠65例. *中医杂志* 50(S1)**,** 165.
- 袁杰, 林丽娜, and 朱建军 (2011). 中西医结合治疗围绝经期失眠症30例临床观察. *江苏中医药* 43(09)**,** 46-47.
- 苑英奎, and 钱仁义 (2016). 中西医结合治疗女性更年期失眠疗效观察. *中医临床研究* 8(15)**,** 59-60.
- 魏枫璐 (2020). 西药联合中药逍遥散加味治疗女性更年期失眠的效果. *中国保健营养* 30(11)**,** 309.
- 张丽萍, and 卢建 (2002). 酸枣仁汤合甘麦大枣汤治疗更年期失眠症25例. *浙江中西医结合杂志* (06)**,** 35-36.
- 张明扬 (2018). 补肾乐眠汤治疗围绝经期妇女顽固性失眠临床观察. *临床医药文献电子杂志* 5(24)**,** 159+162.
- 张文学 (2010). 小柴胡汤加减治疗更年期失眠症研究. *中国医学创新* 7(25)**,** 65-66.
- 张兵 (2018a). 观察栀子豉汤合甘麦大枣汤加减治疗更年期失眠症的临床疗效. *医药前沿* 8(20)**,** 309-310. doi: 10.3969/j.issn.2095-1752.2018.20.269.
- 张兵 (2018b). 栀子豉汤联合甘麦大枣汤加减治疗更年期失眠症的临床疗效观察. *医药前沿* (20)**,** 130-131.
- 张瑞波, and 曾秀娣 (2004). 中西医结合治疗妇女更年期失眠症30例疗效观察. *国际医药卫生导报* (12)**,** 81-82.
- 张素华 (2013). 探讨酸枣仁汤合甘麦大枣汤加减治疗更年期不寐的临床效果. *中国医药指南* 11(10)**,** 681-682.
- 张秀丽, and 王果平 (2007). 西点心脑康胶囊治疗围绝经期失眠症60例. *现代中医药* (02)**,** 21-22.
- 张新成 (2017). 逍遥散加减联合抗焦虑药治疗更年期失眠症的疗效观察. *实用中西医结合临床* 17(11)**,** 84-85.
- 张颖慧 (2009). 参松养心胶囊治疗更年期妇女失眠的疗效观察. *中国医学创新* 6(20)**,** 73-74.
- 张钰 (2018). 酸枣仁汤剂治疗女性更年期失眠效果分析. *医学食疗与健康* (6)**,** 148,151.
- 张瑶, 刘蕾, and 尹倩 (2021). 二仙汤合酸枣仁汤治疗围绝经期失眠疗效及对内分泌激素的影响. *现代中西医结合杂志* 30(32)**,** 3605-3608.
- 张蓉, and 宋李冬 (2012a). 柴胡加龙骨牡蛎汤治疗围绝经期患者失眠的疗效. *中国临床医学* 19(02)**,** 175-176.
- 张蓉, and 宋李冬 (2012b). "柴胡加龙骨牡蛎汤化裁治疗围绝经期失眠的临床疗效观察", in: *中华中医药学会亚健康分会换届选举会议暨第四次“治未病”及亚健康防治论坛论文集.* (昆明).
- 张珍 (2018). 甘麦大枣汤合酸枣仁汤加减治疗更年期脏躁型失眠的临床疗效. *健康必读* (21)**,** 151.
- 张慧芳, and 孟宝丽 (2023). 养肾疏肝解郁汤治疗围绝经期失眠患者的临床疗效及对性激素水平的调节作用. *四川生理科学杂志* 45(1)**,** 69-71.
- 张红芳 (2018). 中药安神丸治疗女性更年期失眠的临床效果观察. *心理医生* 24(10)**,** 146-147.
- 张欢, and 孙晓东 (2017). 黄连阿胶汤合酸枣仁汤治疗更年期失眠的疗效观察. *中国社区医师* 33(17)**,** 92+94.
- 张晓峰, and 张洁 (2020). 桂枝甘草龙骨牡蛎汤加减治疗阴虚火旺型围绝经期期失眠症的临床疗效观察. *保健文汇* (14)**,** 66-67. doi: 10.3969/j.issn.1671-5217.2020.14.046.
- 钱碧霞, 刘姣, 雷先兰, and 江松平 (2017). 丹栀逍遥丸联合坤泰胶囊治疗围绝经期睡眠障碍肝郁化火证临床观察. *新中医* 49(09)**,** 91-93.
- 钱振福 (2013). 二仙汤配合辨证治疗女性围绝经期不寐症70例临床观察. *河北中医* 35(09)**,** 1305-1307.
- 程梦婕, and 胡剑卓 (2017). 加味酸枣仁汤合温胆汤治疗女性更年期失眠疗效观察. *实用中医药杂志* 33(10)**,** 1131-1133.
- 郑捷 (2017). 酸枣仁汤治疗女性更年期失眠60例效果观察. *中国校医* 31(07)**,** 522+525.
- 郑晓人, and 刘南华 (2007). 中西医结合治疗更年期妇女失眠症的临床研究. *现代中西医结合杂志* (19)**,** 2675-2676.
- 曹健 (2008). 中西医结合疗法治疗妇女更年期失眠47例. *世界中医药* (04)**,** 230.
- 赵克华 (2009). 加味二仙汤治疗更年期综合征不寐症156例. *实用中医内科杂志* 23(11)**,** 87-88.
- 宗岩, and 刘枚 (2013). 中西医结合治疗妇女围绝经期睡眠障碍30例临床观察. *江苏中医药* 45(04)**,** 41-42.
- 朱棨耿, and 朱文燕 (2003). “更年助眠饮”治疗更年期妇女失眠50例临床观察. *江苏中医药* (02)**,** 29.
- 朱雯, and 许凌之 (2019). 滋肾解郁宁心方加减治疗围绝经期失眠临床价值分析. *实用妇科内分泌电子杂志* 6(29)**,** 143.
- 周瑞, 刘春丽, and 余欣慧 (2014). 柴胡加龙骨牡蛎汤治疗围绝经期失眠症的临床疗效观察. *山西中医学院学报* 15(04)**,** 70-71.
- 朱蕊, and 康旻 (2012). 补肾潜阳法治疗围绝经期失眠症30例. *陕西中医* 33(7)**,** 785-785. doi: 10.3969/j.issn.1000-7369.2012.07.010.
- 支献峰 (2011). 滋肾舒肝法治疗更年期失眠症71例的疗效观察. *贵阳中医学院学报* 33(2)**,** 40-42. doi: 10.3969/j.issn.1002-1108.2011.02.20.
- 秦斯 (2017). 柴胡加龙骨牡蛎汤加减治疗更年期失眠的疗效观察. *中国保健营养* 27(9)**,** 391-392. doi: 10.3969/j.issn.1004-7484.2017.09.652.
- 秦燕 (2016). 参松养心胶囊治疗更年期女性阴虚火旺型失眠虚症104例疗效观察. *饮食保健* 3(3)**,** 80.
- 陈燕芬, 卢军, 周春泉, 张伯涛, 秦育滨, 林仰锦, et al. (2021). 疏郁化痰法对围绝经期失眠症患者血脂代谢和炎症因子的影响. *中医临床研究* 13(13)**,** 66-69. doi: 10.3969/j.issn.1674-7860.2021.13.020.
- 陈燕芬, 卢军, 叶坚旭, 方米泥, 张伯涛, 林银川, et al. (2020a). 夏仙逍遥饮对围绝经期失眠症患者卵巢功能的影响. *中医临床研究* 12(04)**,** 72-74.
- 陈燕芬, 卢军, 叶坚旭, 方米泥, 张伯涛, 林银川, et al. (2020b). 夏仙逍遥饮对围绝经期失眠症患者胰岛素抵抗及炎症因子的影响. *中国中医药现代远程教育* 18(16)**,** 64-66.
- 陈萍, 王淼, and 吴佳璇 (2014). 李氏引火汤治疗女性更年期不寐58例. *西部中医药* 27(08)**,** 57-58.
- 陈萍, 王淼, and 何凤玲 (2016). 李氏引火汤联合经方治疗女性更年期不寐108例. *西部中医药* 29(12)**,** 66-67.
- 詹群, and 陈霞 (2010). “益坤饮”治疗更年期失眠症30例临床观察. *江苏中医药* 42(05)**,** 44-45.
- 肖高红 (2017). 调冲安神汤对更年期综合征伴失眠患者睡眠质量的影响. *临床医学研究与实践* 2(25)**,** 111-112.
- 肖郡芳 (2005). 加味温胆汤治疗更年期失眠80例. *河南中医* (05)**,** 64-65.
- 肖娟, 王彩娟, 孔祥芳, 王雷, and 平会坤 (2017). 疏肝解郁胶囊联合西医治疗围绝经期失眠疗效观察. *现代中西医结合杂志* 26(23)**,** 2584-2586.
- 彭全利 (2016). 补肾安神汤治疗围绝经期综合征-失眠76例. *临床医学研究与实践* 1(24)**,** 124-125. doi: 10.3969/j.issn.2096-1413.2016.24.061.
- 夏红梅, and 王荣华 (2020). 酸枣仁汤治疗更年期失眠患者的效果. *中外医学研究* 18(02)**,** 47-49.
- 郝锦红 (2011). 解郁安神汤治疗女性更年期失眠临床观察. *中华中医药学刊* 29(07)**,** 1691-1693.
- 韩佳伟 (2020). 用滋肾解郁宁心方加减治疗围绝经期失眠的效果. *当代医药论丛* 18(15)**,** 204-205. doi: 10.3969/j.issn.2095-7629.2020.15.153.
- 韩丽娟, 王思明, and 李胜前 (2014). 宁心安神汤治疗女性更年期失眠67例临床观察. *海峡药学* 26(09)**,** 84-86.
- 黄超, and 邹铭 (2013). 甘麦大枣汤合桂枝龙骨牡蛎汤治疗围绝经期睡眠障碍临床研究. *中国民康医学* 25(24)**,** 62+76.
- 侯京霞 (2018). 研究酸枣仁汤联合西药治疗更年期女性失眠的临床疗效观察. *特别健康* (19)**,** 252-253. doi: 10.3969/j.issn.2095-6851.2018.19.404.
- 侯荣, and 许明跃 (2015). 自拟滋阴降火汤治疗更年期妇女不寐63例疗效观察. *内蒙古中医药* 34(08)**,** 13.

3) not about insomnia (n = 4)

- Li, S., Liu, J., and Zhang, A. (2023). Effect of Kuntai Capsules Combined with Femoston on Sex Hormone Levels and Sleep Status of Patients with Menopausal Syndrome. *New Chinese Medicine* 55(03)**,** 118-121.
- Liang, J., and Li, Z. (2022). Effect of Shugan Bushen Huoxue Decoction on Serum Sex Hormones and Sleep Quality in Patients with Climacteric Syndrome. *RARM* 3(24)**,** 20-23.
- 李燕, 郑树霞, and 胡佩玲 (2022). 滋水清肝饮治疗绝经综合征失眠及焦虑抑郁疗效观察. *时珍国医国药* 33(12)**,** 2960-2962.
- 孙艳 (2013). 滋水清肝饮治疗围绝经期失眠的临床观察. *辽宁中医杂志* 40(12)**,** 2533-2534.

4) comparison between herbal medicines (n = 2)

- 梁桂林 (2019). 滋阴养血、调畅气机法治疗围绝经期失眠临床观察. *山西中医* (8)**,** 19-20,27.
- 杨海龙, 杨海明, and 汪洋 (2016). 百乐眠胶囊联合莉芙敏治疗伴失眠症的更年期综合征的临床观察. *中国医师杂志* 18(10)**,** 1544-1545. doi: 10.3760/cma.j.issn.1008-1372.2016.10.029.

5) not assessing the effects of herbal medicine alone (n = 3)

- 申梅, 赖家湖, and 李舟文 (2018). Clinical effective observation on treating perimenopausal insomnia with TCM medicine plus music. *Clinical Journal of Chinese Medicine* 10(35)**,** 98-99.
- 田文珠 (2023). 中医辨证护理对围绝经期不寐症患者睡眠质量及生活质量的影响. *黑龙江医学* 47(10)**,** 1253-1255.
- 郑万祥, 邵炜军, 吴颂, 王文灵, and 马彦波 (2023). 自拟更年静安膏联合中药怡眠枕治疗围绝经期妇女失眠症的临床研究. *辽宁中医杂志***,** 1-10.

6) not reporting outcomes of interest (n = 1)

- Cao, L., Guo, X., Zhu, P., Wang, L., Rui, M., Song, L., et al. (2018). Effects of Qingxin Zhengan Recipe on serum sex hormones and 5-hydroxytryptamine levels in perimenopausal patients with insomnia. Clinical Journal of Medical Officers 46(2), 139-141. doi: 10.16680/j.1671-3826.2018.02.06.

Supplement 3. Characteristics of the included studies

| **Study ID** | **Sample size analyzed (TG:CG)** | **Mean age (range)  (yr)** | **Insomnia period** | **Pattern identification** | **Comparison** | **Outcomes of interest** | **Adverse events** |
| --- | --- | --- | --- | --- | --- | --- | --- |
| Bai 2014 | 90(45:45) | TG: 49.8 ± 7.1 (42-65) CG: 49.6 ± 7.2 (40-63) | TG: 1.5 ± 0.3y (2m-5y) CG: 1.6 ± 0.2y (3m-5y) | None | EAHM vs. sedative-hypnotics | 1. PSQI 2. Kupperman Index | Not reported |
| Cai 2019 | 134(67:67) | TG: 49.4 ± 5.9 (40-59) CG: 50.3 ± 5.4 (42-60) | TG: 14.6 ± 3.3m (4-25m) CG: 14.1 ± 3.7m (2-23m) | Yin deficiency and fire excess | EAHM + sedative-hypnotics vs. sedative-hypnotics | 1. PSQI 2. TER (insomnia) 3. AE | TG: 3/67  CG: 5/67 |
| Chen 2007 | 80(42:38) | Not reported | TG: 6.4m CG: 7.2m | None | EAHM vs. sedative-hypnotics | 1. Athens Insomnia Scale | Not reported |
| Chen 2012 | 84(42:42) | TG: 49.31 ± 0.52 (45-57) CG: 49.23 ± 0.57 (44-56) | TG: 3m-7y CG: 3m-7y | None | EAHM + sedative-hypnotics vs. sedative-hypnotics | 1. TER (insomnia) | Not reported |
| Chen 2019 | 120(60:60) | 47.45 ± 12.43 (42-55) | 1.34 ± 0.57y (3m-3y) | Liver qi depression and spleen deficiency | EAHM vs. sedative-hypnotics | 1. PSQI 2. AE | TG: 8/60  CG: 12/60 |
| Geng 2014 | 62(32:30) | TG: 50.0 ± 2.3 (45-56) CG: 49.5 ± 2.1 (46-55) | TG: 3.0 ± 1.2y (2-4y) CG: 2.9 ± 1.3y (2-3y) | Yin deficiency and fire excess | EAHM vs. sedative-hypnotics | 1. TER (insomnia) 2. AE | TG: 0/32  CG: 21/30 |
| Guo 2016 | 114(57:57) | TG: 49.61 ± 1.32 (45-56) CG: 49.12 ± 1.62 (44-55) | TG: 27.62 ± 4.62m (6-45m) CG: 27.62 ± 4.31m (5-45m) | None | EAHM vs. sedative-hypnotics | 1. PSQI 2. TER (insomnia) | Not reported |
| Hu 2019 | 60(30:30) | TG: 49.4 ± 2.7 (43-56) CG: 49.4 ± 2.8 (43-55) | TG: 1.7 ± 0.5m (2m-4y) CG: 1.8 ± 0.3m (2m-3y) | None | EAHM vs. sedative-hypnotics | 1. TER (insomnia) 2. AE | TG: 0/30  CG: 2/30 |
| Hu 2020 | 94(47:47) | TG: 50.5 ± 4.4 (47-57) CG: 50.4 ± 4.8 (48-57) | TG: 6.5 ± 2.2m (3-12m) CG: 6.4 ± 2.3m (3-12m) | None | EAHM + sedative-hypnotics vs. sedative-hypnotics | 1. PSQI 2. TER (insomnia) 3. AE | TG: 2/47  CG: 2/47 |
| Hua 2017 | 40(20:20) | TG: 45.9 ± 3.4 CG: 46.3 ± 4.7 | TG: 1.9 ± 0.4y CG: 2.1 ± 0.7y | None | EAHM vs. sedative-hypnotics | 1. TER (insomnia) | Not reported |
| Huang 2020 | 90(45:45) | TG: 49.58 ± 2.16 CG: 50.01 ± 2.08 | TG: 8.17 ± 1.11m CG: 8.22 ± 1.14m | None | EAHM + sedative-hypnotics vs. sedative-hypnotics | 1. PSQI 2. TER (insomnia) 3. AE | TG: 6/45  CG: 7/45 |
| Jia 2008 | 165(110:55) | 48.5 (41-56) | 4.82 ± 3.13y (3m-8y) | None | EAHM + antidepressants vs. antidepressants | 1. TER (insomnia) 2. AE | TG: 37/110  CG: 22/55 |
| Jia 2014 | 80(40:40) | TG: 49.5 ± 2.3 (43-56) CG: 48.7 ± 2.1 (42-58) | TG: 3.5 ± 0.5m (1-5m) CG: 3.3 ± 0.5m (2-4m) | None | EAHM + sedative-hypnotics vs. sedative-hypnotics | 1. TER (insomnia) | Not reported |
| Jia 2023 | 37(20:17) | TG: 47.65 ± 3.83 (42-55) CG: 49.71 ± 3.84 (42-57) | TG: 11.85 ± 9.21m (2-36m) CG: 9.82 ± 7.00m (2-24m) | Non-interaction between the heart and kidney | EAHM vs. sedative-hypnotics | 1. PSQI 2. Kupperman Index 3. TER (insomnia) | Not reported |
| Lai 2011 | 80(40:40) | TG: 48.9 ± 3.0 CG: 48.5 ± 3.3 | TG: 7.9 ± 1.3m (3w-7y) CG: 8.0 ± 1.5m (2w-10y) | None | EAHM vs. sedative-hypnotics | 1. TER (insomnia) | Not reported |
| Lai 2022 | 86(43:43) | TG: 50.31 ± 3.09 (43-54) CG: 50.23 ± 3.18 (45-55) | TG: 9.69 ± 2.78m (5-14m) CG: 9.73 ± 2.83m (5-14m) | Liver-kidney depletion | EAHM + sedative-hypnotics vs. sedative-hypnotics | 1. PSQI (no global score presented) 2. TER (insomnia) | Not reported |
| Lan 2020 | 106(53:53) | TG: 48.22 ± 2.14 (41-56) CG: 48.26 ± 2.17 (40-57) | TG: 6.21 ± 0.57m (3-12m) CG: 6.18 ± 0.53m (3-11m) | None | EAHM vs. sedative-hypnotics | 1. PSQI 2. TER (insomnia) | Not reported |
| Lei 2013 | 68(35:33) | TG: 48.95 ± 3.08 (45-53) CG: 47.36 ± 3.96 (45-53) | TG: 31.5 ± 12.55m (1-108m) CG: 29.3 ± 14.10m (1.5-102m) | Liver depression and kidney deficiency | EAHM vs. sedative-hypnotics | 1. PSQI 2. TER (insomnia) 3. AE | TG: 0/35  CG: 6/33 |
| Li 2015 | 81(42:39) | TG: 48.08 ± 4.42 (44-55) CG: 47.75 ± 4.18 (45-55) | TG: 6.4 ± 2.6m (3m-2y) CG: 6.6 ± 2.2m (3m-2y) | Heart and kidney disharmony | EAHM vs. sedative-hypnotics | 1. Greene Climacteric Scale 2. TER (insomnia) | Not reported |
| Li 2016 | 111(55:56) | TG: 49.4 ± 3.5 (45-55) CG: 49.3 ± 3.5 (45-55) | TG: 7.5 ± 2.2m (4-15m) CG: 7.4 ± 2.2m (4-15m) | None | EAHM vs. sedative-hypnotics | 1. TER (insomnia) | Not reported |
| Li 2018 | 116(58:58) | TG: 50.4 ± 3.5 (45-55) CG: 50.1 ± 3.7 (46-55) | TG: 6.2 ± 1.6m (2-12m) CG: 6.4 ± 1.5m (2-13m) | Kidney yin deficiency | EAHM vs. sedative-hypnotics | 1. PSQI (no global score presented) 2. TER (insomnia) 3. AE | TG: 8/58  CG: 10/58 |
| Li 2021 | 120(60:60) | TG: 47.00 ± 1.85 (37-61) CG: 46.80 ± 2.00 (38-60) | TG: 0.95 ± 0.12y CG: 1.00 ± 0.20y | None | EAHM vs. oryzanol | 1. PSQI 2. Kupperman Index 3. TER (insomnia) 4. AE | TG: 1/60  CG: 2/60 |
| Li 2022 | 60(30:30) | TG: 53.0 ± 6.1 CG: 54.0 ± 5.3 | Not reported | None | EAHM vs. sedative-hypnotics | 1. SRSS 2. Kupperman Index 3. AE | TG: 1/30  CG: 7/30 |
| Li(a) 2020 | 50(25:25) | TG: 49.21 ± 5.03 (45-54) CG: 49.23 ± 4.97 (45-53) | Not reported | None | EAHM vs. sedative-hypnotics | 1. PSQI 2. Kupperman Index 3. TER (insomnia) | Not reported |
| Li(b) 2020 | 70(35:35) | TG: 51.30 ± 3.77 (45-57) CG: 52.31 ± 4.21 (45-58) | TG: 1.32 ± 0.55y (0.5-4.5y) CG: 1.21 ± 0.73y (0.5-5y) | None | EAHM + estradiol vs. estradiol | 1. PSQI (no global score presented) 2. Kupperman Index | Not reported |
| Liu 2021 | 62(29:33) | TG: 49.1 ± 1.2 (44-54) CG: 48.8 ± 1.2 (43-54) | TG: 10.5 ± 1.5m (2-20m) CG: 10.2 ± 1.5m (1-19m) | None | EAHM vs. sedative-hypnotics | 1. TER (insomnia) | Not reported |
| Lu 2007 | 99(52:47) | 51 (45-55) | 22m (6m-4y) | None | EAHM vs. oryzanol | 1. TER (insomnia) | Not reported |
| Lu 2019 | 86(43:43) | TG: 50.32 ± 5.57 (42-59) CG: 51.15 ± 6.83 (40-61) | TG: 12.32 ± 3.71m (6-22m) CG: 13.05 ± 4.18y (5-20m) | Yin deficiency and fire excess | EAHM vs. sedative-hypnotics | 1. PSQI 2. TER (insomnia) | Not reported |
| Luo 2021 | 80(40:40) | TG: 49.4 ± 2.2 (45-55) CG: 49.6 ± 2.5 (45-53) | TG: 3.4 ± 0.5d (1-14d) CG: 3.6 ± 0.3d (1-12d) | None | EAHM + antidepressants vs. antidepressants | 1. PSQI 2. AE | TG: 2/40  CG: 9/40 |
| Ma 2018 | 50(25:25) | 51.12 ± 4.32 (46-57) | 3.51 ± 0.13m (2-4m) | None | EAHM + sedative-hypnotics vs. sedative-hypnotics | 1. TER (insomnia) 2. AE | TG: 3/25  CG: 2/25 |
| Mahmoudi 2020 | 106(53:53) | TG: 58.45 ± 5.60 CG: 57.06 ± 6.54 | Not reported | None | EAHM vs. placebo EAHM | 1. PSQI | Not reported |
| Mao 2020 | 90(45:45) | TG: 50.82 ± 3.26 (45-57) CG: 50.30 ± 3.41 (43-59) | TG: 9.63 ± 2.15m (3-17m) CG: 9.27 ± 2.30m (4-18m) | Non-interaction between the heart and kidney | EAHM + sedative-hypnotics vs. sedative-hypnotics | 1. PSQI (no global score presented) 2. TER (insomnia) | Not reported |
| Mi 2022 | 68(34:34) | TG: 49.97 ± 4.49 CG: 51.15 ± 3.43 | TG: 12m CG: 12m | Liver-kidney deficiency | EAHM vs. placebo EAHM | 1. PSQI (SD value was not presented) 2. Kupperman Index (SD value was not presented) 3. TER (insomnia) 4. AE | TG: 0/34  CG: 0/34 |
| Pan 2022 | 130(65:65) | TG: 52.50 ± 0.26 (41-62) CG: 52.50 ± 0.28 (42-63) | TG: 0.50 ± 1.03m (5-36m) CG: 20.45 ± 1.02m (4-36m) | Liver qi depression | EAHM + sedative-hypnotics vs. sedative-hypnotics | 1. PSQI 2. TER (insomnia) | Not reported |
| Pang 2019 | 50(25:25) | TG: 54.23 ± 2.16 (44-65) CG: 54.28 ± 2.24 (43-66) | TG: 2.45 ± 0.34y (4m-5y) CG: 2.48 ± 0.37y (5m-5y) | None | EAHM + sedative-hypnotics vs. sedative-hypnotics | 1. PSQI 2. TER (insomnia) 3. AE | TG: 3/25  CG: 4/25 |
| Pu 2019 | 60(30:30) | TG: 46.87 ± 5.41 (41-58) CG: 46.98 ± 5.37 (42-56) | TG: 1.53 ± 0.51y (4m-5y) CG: 1.55 ± 0.46m (6m-4.5y) | Liver-kidney yin deficiency | EAHM + sedative-hypnotics vs. sedative-hypnotics | 1. PSQI (no global score presented) 2. TER (insomnia) | Not reported |
| Qiao 2021 | 58(29:29) | TG: 51.00 ± 2.79 (45-54) CG: 49.63 ± 3.35 (45-55) | TG: 8.93 ± 3.66m (5-14m) CG: 9.79 ± 3.73m (4-17m) | Heart-kidney noninteraction | EAHM + sedative-hypnotics vs. sedative-hypnotics | 1. PSQI 2. Kupperman Index (SD value was not presented) 3. TER (insomnia) 4. AE | TG: 5/29  CG: 13/29 |
| Qin 2023 | 80(40:40) | TG: 48.15 ± 2.15 (44-55) CG: 48.03 ± 1.83 (45-55) | TG: 11.55 ± 1.25m (6-24m) CG: 11.52 ± 1.22m (6-24m) | Liver depression and spleen deficiency | EAHM + sedative-hypnotics vs. sedative-hypnotics | 1. PSQI 2. TER (insomnia) 3. AE | TG: 6/40  CG: 7/40 |
| Rui 2017 | 78(39:39) | TG: 48.16 ± 3.59 (42-54) CG: 48.31 ± 3.65 (43-55) | TG: 4.78 ± 1.39m (1-12m) CG: 4.95 ± 1.40m (2-12m) | None | EAHM + sedative-hypnotics vs. sedative-hypnotics | 1. PSQI 2. PSG 1) total sleep time (min) 2) REM sleep time (min) 3) sleep latency (min) 4) waking time (min) 5) sleep efficiency (%) | There was no significant difference between groups. (No raw data was presented) |
| Shan 2020 | 60(30:30) | TG: 48.78 ± 2.71 CG: 49.34 ± 3.07 | TG: 3.76 ± 1.27y CG: 3.58 ± 1.18y | None | EAHM + sedative-hypnotics vs. sedative-hypnotics | 1. PSQI 2. TER (insomnia) 3. AE | TG: 3/30  CG: 5/30 |
| Shen 2018 | 97(49:48) | TG: 52.92 ± 3.21 (45-59) CG: 53.51 ± 3.47 (47-60) | TG: 2.17 ± 0.54y (3m-5y) CG: 2.09 ± 0.49y (4m-4y) | None | EAHM + sedative-hypnotics vs. sedative-hypnotics | 1. PSQI 2. Kupperman Index 3. TER (insomnia) 4. AE | TG: 0/49  CG: 0/48 |
| Shi 2018 | 70(35:35) | TG: 51.5 ± 1.2 (45-57) CG: 51.9 ± 1.1 (45-56) | TG: 7.8 ± 2.1m (4-12m) CG: 7.5 ± 2.0m (4-11m) | None | EAHM + sedative-hypnotics vs. sedative-hypnotics | 1. PSQI | Not reported |
| Su 2021 | 91(48:43) | TG: 47.3 ± 2.1 CG: 50.3 ± 3.2 | TG: 8.3 ± 1.2m CG: 7.9 ± 2.2m | Kidney yinyang imbalance | EAHM + sedative-hypnotics vs. sedative-hypnotics | 1. Spiegel Sleep Questionnaire 2. TER (insomnia) 3. AE | TG: 0/48  CG: 2/43 |
| Sun 2016 | 92(46:46) | TG: 47.8 ± 2.3 (40-55) CG: 48.2 ± 2.1 (42-55) | TG: 6.7 ± 1.5m (2-15m) CG: 6.5 ± 1.3m (1-13m) | Internal harassment of phlegm-heat | EAHM vs. sedative-hypnotics | 1. PSQI (no global score presented) 2. TER (insomnia) | Not reported |
| Sun 2020 | 86(43:43) | TG: 48.61 ± 7.09 (40-68) CG: 48.65 ± 7.13 (38-64) | TG: 2.79 ± 0.61y (8m-5y) CG: 2.81 ± 0.64y (6m-4y) | None | EAHM vs. sedative-hypnotics | 1. PSQI 2. TER (insomnia) | Not reported |
| Wang 2014 | 100(50:50) | TG: 47.1 ± 4.5 CG: 46.3 ± 4.2 | TG: 12.8 ± 7.2m (1-28m) CG: 13.4 ± 7.3m (1-28m) | None | EAHM vs. sedative-hypnotics | 1. PSQI 2. SRSS 3. AE | TG: 0/50  CG: 8/50 |
| Wang 2023 | 60(30:30) | TG: 48.87 ± 3.85 CG: 48.33 ± 3.35 | TG: 5.97 ± 4.03m CG: 6.20 ± 3.15m | Kidney deficiency and liver stagnation | EAHM vs. estradiol | 1. PSQI 2. Kupperman Index 3. TER (insomnia) | Not reported |
| Wang(a) 2018 | 77(39:38) | TG: 47 ± 5.87 (43-52) CG: 47 ± 6.21 (43-53) | TG: 3 ± 2.34m CG: 3 ± 3.47m | None | EAHM + antidepressants vs. antidepressants | 1. TER (insomnia) | Not reported |
| Wang(a) 2021 | 50(25:25) | TG: 62.6 ± 2.4 (54-78) CG: 62.3 ± 2.6 (53-79) | Not reported | None | EAHM vs. sedative-hypnotics | 1. PSQI 2. TER (insomnia) | Not reported |
| Wang(a) 2022 | 78(39:39) | TG: 48.92 ± 3.11 (45-60) CG: 49.42 ± 3.41 (44-59) | TG: 14.21 ± 3.61m (4-30m) CG: 13.56 ± 3.01m (5-28m) | None | EAHM vs. sedative-hypnotics | 1. PSQI 2. TER (insomnia) 3. AE | TG: 0/39  CG: 4/39 |
| Wang(b) 2018 | 52(26:26) | TG: 49.1 ± 7.1 (45-55) CG: 49.0 ± 6.9 (45-54) | TG: 0.9 ± 0.2y (3-24m) CG: 0.9 ± 0.3y (4-25m) | Kidney deficiency and liver stagnation | EAHM vs. sedative-hypnotics | 1. PSQI 2. TER (insomnia) | Not reported |
| Wang(b) 2021 | 138(69:69) | TG: 49.12 ± 2.20 (45-54) CG: 48.98 ± 2.45 (43-53) | 10.74 ± 1.56m (6-17m) | Liver blood deficiency | EAHM + sedative-hypnotics vs. sedative-hypnotics | 1. PSQI 2. TER (insomnia) 3. AE | TG: 4/69  CG: 7/69 |
| Wang(b) 2022 | 60(30:30) | TG: 49.6 ± 2.1 (46-54) CG: 49.8 ± 2.2 (45-54) | TG: 9.2 ± 1.2m (4-16m) CG: 9.4 ± 1.3m (4-17m) | None | EAHM + sedative-hypnotics vs. sedative-hypnotics | 1. PSQI 2. Kupperman Index 3. TER (insomnia) 4. AE | TG: 0/30  CG: 0/30 |
| Wei 2020 | 300(150:150) | TG: 51.6 ± 3.4 (47-60) CG: 52.2 ± 4.3 (45-61) | Not reported | Kidney deficiency and liver stagnation | EAHM vs. sedative-hypnotics | 1. PSQI 2. Kupperman Index 3. TER (insomnia) | Not reported |
| Weng 2016 | 120(60:60) | 47.6 ± 5.8 (45-55) | Not reported | Deficiency of blood and yin | EAHM vs. sedative-hypnotics | 1. PSQI (no global score presented) 2. TER (insomnia) 3. AE | TG: 0/60  CG: 8/60 |
| Xing 2018 | 56(28:28) | TG: (44-58) CG: (45-59) | TG: (2-26m) CG: (3-27m) | None | EAHM vs. sedative-hypnotics | 1. TER (insomnia) 2. AE | TG: 0/28  CG: 1/28 |
| Xu 2023 | 100(50:50) | 49.6 ± 2.5 (46-55) | 1.5 ± 0.4y (1-3y) | Liver-kidney yin deficiency | EAHM vs. sedative-hypnotics | 1. PSQI 2. Spiegel Sleep Questionnaire 3. TER (insomnia) | Not reported |
| Yao 2014 | 90(45:45) | TG: 51.9 ± 8.8 (41-60) CG: 52.6 ± 8.6 (42-60) | Not reported | None | EAHM + losartan potassium vs. losartan potassium | 1. TER (insomnia) | Not reported |
| Ye 2015 | 60(30:30) | TG: 50.78 ± 5.84 CG: 49.25 ± 4.47 | Not reported | None | EAHM vs. sedative-hypnotics | 1. PSQI 2. TER (insomnia) | Not reported |
| You 2022 | 86(43:43) | TG: 50.10 ± 2.41 (45-55) CG: 50.15 ± 2.36 (45-55) | TG: 7.01 ± 1.22m (3-12m) CG: 6.95 ± 1.24m (3-12m) | None | EAHM + sedative-hypnotics vs. sedative-hypnotics | 1. PSQI 2. Kupperman Index 3. TER (insomnia) | Not reported |
| Zeng 2022 | 32(16:16) | TG: 50.13 ± 2.13 CG: 50.31 ± 2.63 | Not reported | Heart-kidney noninteraction | EAHM vs. placebo EAHM | 1. PSQI 2. Kupperman Index 3. AE | TG: 0/16  CG: 0/16 |
| Zhang 2009 | 60(30:30) | TG: 49.5 ± 7.6 CG: 48.2 ± 7.3 | TG: 10.6 ± 4.2m CG: 9.9 ± 4.6m | Heart-kidney noninteraction | EAHM vs. sedative-hypnotics | 1. Athens Insomnia Scale 2. AE | TG: 0/30  CG: 0/30 |
| Zhang 2012 | 67(35:32) | TG: 48.26 ± 1.13 CG: 49.17 ± 0.9 | TG: 2.03 ± 0.42y CG: 2.15 ± 0.35y | Liver-kidney deficiency | EAHM vs. sedative-hypnotics | 1. PSQI 2. TER (insomnia) 3. AE | TG: 1/35  CG: 13/32 |
| Zhang 2020 | 116(58:58) | TG: 51 CG: 50 | TG: 12m CG: 10m | Kidney deficiency and liver stagnation | EAHM + sedative-hypnotics vs. sedative-hypnotics | 1. PSQI 2. AE | TG: 1/58  CG: 0/58 |
| Zhang 2021 | 80(40:40) | TG: 50.57 ± 7.01 (43-60) CG: 50.8 ± 7.64 (41-58) | TG: 3.15 ± 2.43m (1-6m) CG: 3.15 ± 2.51m (1-6.5m) | None | EAHM vs. sedative-hypnotics | 1. PSQI 2. TER (insomnia) | Not reported |
| Zhao 2017 | 120(62:58) | TG: 47.78 ± 4.42 CG: 48.56 ± 3.32 | TG: 13.8 ± 3.54m (1-37m) CG: 14 ± 3.24m (1-36m) | None | EAHM vs. sedative-hypnotics | 1. PSQI 2. TER (insomnia) 3. AE | TG: 4 cases  CG: 57 cases  (This refers to the total number of occurrences, not the number of participants who experienced an adverse reaction) |
| Zhao 2018 | 120(60:60) | TG: 51.06 ± 2.35 (45-55) CG: 50.13 ± 3.26 (46-55) | Not reported | None | EAHM vs. sedative-hypnotics | 1. TER (insomnia) | Not reported |
| Zheng 2016 | 60(30:30) | TG: 43.21 ± 7.67 CG: 41.97 ± 11.25 | TG: 19.43 ± 9.28m (12-96m) CG: 18.48 ± 7.69m (11-96m) | None | EAHM + sedative-hypnotics vs. sedative-hypnotics | 1. PSQI 2. TER (insomnia) | Not reported |
| Zheng 2020 | 100(50:50) | TG: 47.55 ± 2.87 (41-54) CG: 50.55 ± 2.72 (44-57) | TG: 2.07 ± 0.43y (1-3y) CG: 3.06 ± 0.36y (1-5y) | None | EAHM vs. sedative-hypnotics | 1. TER (insomnia) | Not reported |
| Zhu 2020 | 62(31:31) | TG: 48.6 ± 4.9 (41-60) CG: 48.3 ± 4.8 (41-60) | TG: 12.4 ± 5.5m (2-24m) CG: 12.3 ± 5.6m (3-24m) | None | EAHM vs. sedative-hypnotics | 1. PSQI 2. TER (insomnia) 3. AE | TG: 2/31  CG: 18/31 |

AE, adverse events; CG, control group; EAHM, East Asian herbal medicine; PSQI, Pittsburgh sleep quality index; SRSS, sleep state self-rating scale; TER, total effective rate; TG, treatment group.

Supplement 4. Details of herbal medicines and control group interventions for the included studies

| **Study ID** | **Comparison** | **HM name** | **Preparation form** | **EAHM composition** | **Control group** | **Pharmaceutical producer** | **Quality control measures reported** | **Chemical analysis reported** | **Administration period** | **Follow-up period** |
| --- | --- | --- | --- | --- | --- | --- | --- | --- | --- | --- |
| Bai 2014 | EAHM vs. sedative-hypnotics | Bushen Lemian Tang | Decoction | Rehmannia glutinosa (Gaertn.) DC. [Orobanchaceae; Rehmanniae Radix Preparata], Paeonia × suffruticosa Andrews [Paeoniaceae; Moutan Radicis Cortex] 20g, Cornus officinalis Siebold & Zucc. [Cornaceae; Corni Fructus], Zizyphus jujuba Miller var. spinosa Hu ex H. F. Chou [Rhamnaceae; Zizyphi Semen], Dioscorea oppositifolia L. [Dioscoreaceae; Dioscoreae Rhizoma], Albizzia julibrissin Durazzini [Leguminosae; Albizziae Cortex], Atractylodes lancea (Thunb.) DC. [Asteraceae; Atractylodis Rhizoma Alba] 15g, Epimedium sagittatum (Siebold & Zucc.) Maxim. [Berberidaceae; Epimedii Herba], Uncaria rhynchophylla (Miq.) Miq. [Rubiaceae; Uncariae Ramulus cum Uncus], Curculigo orchioides Gaertner [Amarylidaceae; Curculiginis Rhizoma], Gastrodia elata Blume [Orchidaceae; Gastrodiae Rhizoma], Polygala senega L. [Polygalaceae; Polygalae Radix], Cullen corylifolium (L.) Medik. [Fabaceae; Psoraleae Semen] 10g | Diazepam 1-2mg hs, Medroxyprogesterone Acetate Complex Capsule 2C qd | Not reported | Not reported | Not reported | 4 weeks | None |
| Cai 2019 | EAHM + sedative-hypnotics vs. sedative-hypnotics | modified Guizhi Gancao Longgu Muli Tang | Decoction | Neolitsea cassia (L.) Kosterm. [Lauraceae; Cinnamomi Ramulus] 15g, Ophiopogon japonicus (Thunb.) Ker Gawl. [Asparagaceae; Liriopis seu Ophiopogonis Tuber] 10g, Anemarrhena asphodeloides Bunge [Asparagaceae; Anemarrhenae Rhizoma], Ostrea gigas Thunberg [Ostreidae; Ostreae Testa], Polygala senega L. [Polygalaceae; Polygalae Radix], Zizyphus jujuba Miller var. spinosa Hu ex H. F. Chou [Rhamnaceae; Zizyphi Semen], Fossilia Ossis Mastodi 9g, Glycyrrhiza glabra L. [Fabaceae; Glycyrrhizae Radix et Rhizoma] 6g | Eszopiclone 3mg hs | Not reported | Not reported | Not reported | 4 weeks | None |
| Chen 2007 | EAHM vs. sedative-hypnotics | NR | Decoction | Reynoutria multiflora (Thunb.) Moldenke [Polygonaceae; Polygoni Multiflori Caulis] 30g, Chinemys reevesii Gray [Emydidae; Testudinis Chinemis Plastrum et Carapax], Ligustrum lucidum W.T.Aiton [Oleaceae; Ligustri Fructus], Eclipta prostrata Linné [Compositae; Ecliptae Herba], Rehmannia glutinosa (Gaertner) Liboschitz ex Steudel [Scrophulariaceae; Rehmanniae Radix Recens], Zao Pi [Rhamnaceae; Ziziphus jujuba mill. var. inermis (bge.) rehd.], Zizyphus jujuba Miller var. spinosa Hu ex H. F. Chou [Rhamnaceae; Zizyphi Semen] 20g, Lycium chinense Miller [Solanaceae; Lycii Fructus], Paeonia lactiflora Pall. [Paeoniaceae; Paeoniae Radix] 15g, Paeonia × suffruticosa Andrews [Paeoniaceae; Moutan Radicis Cortex], Poria cocos Wolf [Polyporaceae; Poria Sclertum Cum Pini Radix], Epimedium sagittatum (Siebold & Zucc.) Maxim. [Berberidaceae; Epimedii Herba], Anemarrhena asphodeloides Bunge [Asparagaceae; Anemarrhenae Rhizoma] 12g | Estazolam 1mg hs | Not reported | Not reported | Not reported | 2 months | 6 months |
| Chen 2012 | EAHM + sedative-hypnotics vs. sedative-hypnotics | Zuogui Pill | Decoction | Rehmannia glutinosa (Gaertn.) DC. [Orobanchaceae; Rehmanniae Radix Preparata], Poria cocos Wolf [Polyporaceae; Poria Sclertum Cum Pini Radix], Dioscorea oppositifolia L. [Dioscoreaceae; Dioscoreae Rhizoma], Cornus officinalis Siebold & Zucc. [Cornaceae; Corni Fructus], Lycium chinense Miller [Solanaceae; Lycii Fructus], Zizyphus jujuba Miller var. spinosa Hu ex H. F. Chou [Rhamnaceae; Zizyphi Semen], Reynoutria multiflora (Thunb.) Moldenke [Polygonaceae; Polygoni Multiflori Caulis], Ostrea gigas Thunberg [Ostreidae; Ostreae Testa], Fossilia Ossis Mastodi, Paeonia lactiflora Pall. [Paeoniaceae; Paeoniae Radix] 15g | Oryzanol 10-30mg tid, Diazepam 5-10mg hs | Not reported | Not reported | Not reported | 8 weeks | None |
| Chen 2019 | EAHM vs. sedative-hypnotics | (1st month) Chaihu Jia Longgu Muli Tang, (2nd-3rd month) modified Guipi Tang | Decoction | (1st month) Fossilia Ossis Mastodi, Ostrea gigas Thunberg [Ostreidae; Ostreae Testa] 30g, Codonopsis pilosula (Franch.) Nannf. [Campanulaceae; Codonopsis Pilosulae Radix] 20g, Poria cocos Wolf [Polyporaceae; Poria Sclerotium], Poria cocos Wolf [Polyporaceae; Poria Sclertum Cum Pini Radix] 15g, Neolitsea cassia (L.) Kosterm. [Lauraceae; Cinnamomi Ramulus] 12g, Bupleurum falcatum L. [Apiaceae; Bupleuri Radix], Angelica gigas Nakai [Apiaceae; Angelicae Gigantis Radix], Zizyphus jujuba Miller var. spinosa Hu ex H. F. Chou [Rhamnaceae; Zizyphi Semen], Pueraria montana var. lobata (Willd.) Maesen & S.M.Almeida ex Sanjappa & Predeep [Fabaceae; Puerariae Radix], Albizzia Julibrissin [Leguminosae; Albizziae Flos] 10g, Atractylodes lancea (Thunb.) DC. [Asteraceae; Atractylodis Rhizoma] 9g, Prunus persica (L.) Batsch [Rosaceae; Persicae Semen] 6g, Coptis chinensis Franch. [Ranunculaceae; Coptidis Rhizoma], Glycyrrhiza glabra L. [Fabaceae; Glycyrrhizae Radix et Rhizoma] 3g (2nd-3rd month) Astragalus mongholicus Bunge [Fabaceae; Astragali Radix], Poria cocos Wolf [Polyporaceae; Poria Sclerotium], Poria cocos Wolf [Polyporaceae; Poria Sclertum Cum Pini Radix] 15g, Angelica gigas Nakai [Apiaceae; Angelicae Gigantis Radix], Pueraria montana var. lobata (Willd.) Maesen & S.M.Almeida ex Sanjappa & Predeep [Fabaceae; Puerariae Radix], Albizzia Julibrissin [Leguminosae; Albizziae Flos] 10g, Codonopsis pilosula (Franch.) Nannf. [Campanulaceae; Codonopsis Pilosulae Radix], Bupleurum falcatum L. [Apiaceae; Bupleuri Radix], Citrus × aurantium L. [Rutaceae; Aurantii Fructus Immaturus], Cyperus rotundus L. [Cyperaceae; Cyperi Rhizoma], Atractylodes lancea (Thunb.) DC. [Asteraceae; Atractylodis Rhizoma] 9g, Prunus persica (L.) Batsch [Rosaceae; Persicae Semen], Polygala senega L. [Polygalaceae; Polygalae Radix], Dolomiaea costus (Falc.) Kasana & A.K.Pandey [Asteraceae; Aucklandiae Radix] 5g, Glycyrrhiza glabra L. [Fabaceae; Glycyrrhizae Radix et Rhizoma] 3g | Estazolam 2mg hs | Not reported | Not reported | Not reported | 3 months | None |
| Geng 2014 | EAHM vs. sedative-hypnotics | Danggui Liuhuang Tang | Decoction | Astragalus mongholicus Bunge [Fabaceae; Astragali Radix] 30g, Angelica gigas Nakai [Apiaceae; Angelicae Gigantis Radix] 15g, Phellodendron amurense Rupr. [Rutaceae; Phellodendri Cortex], Scutellaria baicalensis Georgi [Lamiaceae; Scutellariae Radix], Rehmannia glutinosa (Gaertner) Liboschitz ex Steudel [Scrophulariaceae; Rehmanniae Radix Recens] 12g, Coptis chinensis Franch. [Ranunculaceae; Coptidis Rhizoma], Rehmannia glutinosa (Gaertn.) DC. [Orobanchaceae; Rehmanniae Radix Preparata] 10g | Diazepam 1-4mg hs | Not reported | Not reported | Not reported | 4 weeks | None |
| Guo 2016 | EAHM vs. sedative-hypnotics | Anshen Tang | Decoction | Ostrea gigas Thunberg [Ostreidae; Ostreae Testa], Reynoutria multiflora (Thunb.) Moldenke [Polygonaceae; Polygoni Multiflori Caulis], Fossilia Ossis Mastodi, Zizyphus jujuba Miller var. spinosa Hu ex H. F. Chou [Rhamnaceae; Zizyphi Semen] 30g, Albizzia julibrissin Durazzini [Leguminosae; Albizziae Cortex], Salvia miltiorrhiza Bunge [Lamiaceae; Salviae Miltiorrhizae Radix], Rosa rugosa Thunberg [Rosaceae; Rosae Rugosae Flos] 20g, Scutellaria baicalensis Georgi [Lamiaceae; Scutellariae Radix], Bupleurum falcatum L. [Apiaceae; Bupleuri Radix], Neolitsea cassia (L.) Kosterm. [Lauraceae; Cinnamomi Ramulus], Pinellia ternata (Thunb.) Makino [Araceae; Pinelliae Tuber] 10g, Glycyrrhiza glabra L. [Fabaceae; Glycyrrhizae Radix et Rhizoma] 6g, Rheum officinale Baill. [Polygonaceae; Rhei Radix et Rhizoma] 3g | Estazolam 1-2mg hs | Not reported | Not reported | Not reported | 30 days | None |
| Hu 2019 | EAHM vs. sedative-hypnotics | Huanglian Ejiao Tang plus Ganmai Dazao Tang | Decoction | Triticum aestivum Linné [Gramineae; Tritici Fructus Levis] 30g, Scutellaria baicalensis Georgi [Lamiaceae; Scutellariae Radix], Paeonia lactiflora Pall. [Paeoniaceae; Paeoniae Radix], Poria cocos Wolf [Polyporaceae; Poria Sclerotium], Albizzia julibrissin Durazzini [Leguminosae; Albizziae Cortex] 15g, Equus asinus Linne [Equidae; Asini Corii Colla], Glycine max Merrill [Leguminosae; Glycine Semen Preparata], Gardenia jasminoides J.Ellis [Rubiaceae; Gardeniae Fructus], Glycyrrhiza glabra L. [Fabaceae; Glycyrrhizae Radix et Rhizoma] 10g, Coptis chinensis Franch. [Ranunculaceae; Coptidis Rhizoma] 6g, Gallus domesticus [Phasianidae; Galli Vitellus] 1 piece, Ziziphus jujuba Mill. [Rhamnaceae; Zizyphi Fructus] 5 pieces | Diazepam 2.5g bid | Not reported | Not reported | Not reported | 1 month | None |
| Hu 2020 | EAHM + sedative-hypnotics vs. sedative-hypnotics | Sheng Yu Tang | Decoction | Astragalus mongholicus Bunge [Fabaceae; Astragali Radix] 30g, Codonopsis pilosula (Franch.) Nannf. [Campanulaceae; Codonopsis Pilosulae Radix] 15g, Angelica gigas Nakai [Apiaceae; Angelicae Gigantis Radix], Rehmannia glutinosa (Gaertner) Liboschitz ex Steudel [Scrophulariaceae; Rehmanniae Radix Recens], Conioselinum anthriscoides 'Chuanxiong' [Apiaceae; Cnidii Rhizoma], Paeonia lactiflora Pall. [Paeoniaceae; Paeoniae Radix], Gardenia jasminoides J.Ellis [Rubiaceae; Gardeniae Fructus], Paeonia × suffruticosa Andrews [Paeoniaceae; Moutan Radicis Cortex] 10g | Zopiclone 7.5mg qd | Not reported | Not reported | Not reported | 4 weeks | None |
| Hua 2017 | EAHM vs. sedative-hypnotics | Jieyu Anshen Tang | Decoction | Reynoutria multiflora (Thunb.) Moldenke [Polygonaceae; Polygoni Multiflori Caulis], Ostrea gigas Thunberg [Ostreidae; Ostreae Testa], Fossilia Ossis Mastodi, Zizyphus jujuba Miller var. spinosa Hu ex H. F. Chou [Rhamnaceae; Zizyphi Semen] 30g, Albizzia julibrissin Durazzini [Leguminosae; Albizziae Cortex], Rosa rugosa Thunberg [Rosaceae; Rosae Rugosae Flos], Salvia miltiorrhiza Bunge [Lamiaceae; Salviae Miltiorrhizae Radix] 20g, Neolitsea cassia (L.) Kosterm. [Lauraceae; Cinnamomi Ramulus], Pinellia ternata (Thunb.) Makino [Araceae; Pinelliae Tuber], Scutellaria baicalensis Georgi [Lamiaceae; Scutellariae Radix], Bupleurum falcatum L. [Apiaceae; Bupleuri Radix] 10g, Glycyrrhiza glabra L. [Fabaceae; Glycyrrhizae Radix et Rhizoma] 6g, Rheum officinale Baill. [Polygonaceae; Rhei Radix et Rhizoma] 3g | Zopiclone 7.5mg hs | Not reported | Not reported | Not reported | NR | None |
| Huang 2020 | EAHM + sedative-hypnotics vs. sedative-hypnotics | Hei Xiaoyao San | Decoction | Rehmannia glutinosa (Gaertn.) DC. [Orobanchaceae; Rehmanniae Radix Preparata], Paeonia lactiflora Pall. [Paeoniaceae; Paeoniae Radix] 20g, Angelica gigas Nakai [Apiaceae; Angelicae Gigantis Radix], Poria cocos Wolf [Polyporaceae; Poria Sclerotium], Atractylodes lancea (Thunb.) DC. [Asteraceae; Atractylodis Rhizoma Alba] 15g, Bupleurum falcatum L. [Apiaceae; Bupleuri Radix] 10g, Glycyrrhiza glabra L. [Fabaceae; Glycyrrhizae Radix et Rhizoma] 6g | Estazolam 2mg hs | Not reported | Not reported | Not reported | 4 weeks | None |
| Jia 2008 | EAHM + antidepressants vs. antidepressants | Gengnian Anshen Tang | Decoction | Zizyphus jujuba Miller var. spinosa Hu ex H. F. Chou [Rhamnaceae; Zizyphi Semen] 30g, Rehmannia glutinosa (Gaertner) Liboschitz ex Steudel [Scrophulariaceae; Rehmanniae Radix Recens], Ophiopogon japonicus (Thunb.) Ker Gawl. [Asparagaceae; Liriopis seu Ophiopogonis Tuber], Cornus officinalis Siebold & Zucc. [Cornaceae; Corni Fructus], Poria cocos Wolf [Polyporaceae; Poria Sclertum Cum Pini Radix] 15g, Cuscuta chinensis Lam. [Convolvulaceae; Cuscutae Semen], Epimedium sagittatum (Siebold & Zucc.) Maxim. [Berberidaceae; Epimedii Herba], Schisandra chinensis (Turcz.) Baill. [Schisandraceae; Schisandrae Fructus] 10g, Polygala senega L. [Polygalaceae; Polygalae Radix], Bupleurum falcatum L. [Apiaceae; Bupleuri Radix] 6g, Glycyrrhiza glabra L. [Fabaceae; Glycyrrhizae Radix et Rhizoma] 5g, Coptis chinensis Franch. [Ranunculaceae; Coptidis Rhizoma] 3g | Mirtazapine 30mg/d to 45mg/d | Not reported | Not reported | Not reported | 4-8 weeks | None |
| Jia 2014 | EAHM + sedative-hypnotics vs. sedative-hypnotics | NR | Decoction | Poria cocos Wolf [Polyporaceae; Poria Sclerotium], Rehmannia glutinosa (Gaertn.) DC. [Orobanchaceae; Rehmanniae Radix Preparata], Dioscorea oppositifolia L. [Dioscoreaceae; Dioscoreae Rhizoma], Cornus officinalis Siebold & Zucc. [Cornaceae; Corni Fructus], Fossilia Ossis Mastodi, Ostrea gigas Thunberg [Ostreidae; Ostreae Testa] 15g, Citrus trifoliata L. [Rutaceae; Ponciri Fructus Immaturus] 12g, Pinellia ternata (Thunb.) Makino [Araceae; Pinelliae Tuber], Citrus × aurantium f. deliciosa (Ten.) M.Hiroe [Rutaceae; Citri Unshius Pericarpium], Phyllostachys nigra Munro var. henonsis Stapf [Gramineae; Phyllostachyos Caulis in Taeniam], Schisandra chinensis (Turcz.) Baill. [Schisandraceae; Schisandrae Fructus], Lycium chinense Miller [Solanaceae; Lycii Fructus] 9g, Conioselinum anthriscoides 'Chuanxiong' [Apiaceae; Cnidii Rhizoma], Glycyrrhiza glabra L. [Fabaceae; Glycyrrhizae Radix et Rhizoma] 6g | Alprazolam 0.4mg hs | Not reported | Not reported | Not reported | 1 month | None |
| Jia 2023 | EAHM vs. sedative-hypnotics | Geng Xin Tang | Granule | Fossilia Ossis Mastodi, Ostrea gigas Thunberg [Ostreidae; Ostreae Testa] 30g, Ligustrum lucidum W.T.Aiton [Oleaceae; Ligustri Fructus], Eclipta prostrata Linné [Compositae; Ecliptae Herba], Rehmannia glutinosa (Gaertner) Liboschitz ex Steudel [Scrophulariaceae; Rehmanniae Radix Recens], Paeonia lactiflora Pall. [Paeoniaceae; Paeoniae Radix], Lycium chinense Miller [Solanaceae; Lycii Fructus], Lilium lancifolium Thunb. [Liliaceae; Lilii Bulbus], Salvia miltiorrhiza Bunge [Lamiaceae; Salviae Miltiorrhizae Radix], Astragalus complanatus R. Brown [Leguminosae; Astragali Complanati Semen], Albizzia julibrissin Durazzini [Leguminosae; Albizziae Cortex], Reynoutria multiflora (Thunb.) Moldenke [Polygonaceae; Polygoni Multiflori Caulis] 15g, Anemarrhena asphodeloides Bunge [Asparagaceae; Anemarrhenae Rhizoma] 12g, Tribulus terrestris [Zygophyllaceae; Tribuli Fructus] 10g, Nelumbo nucifera [Nymphaeaceae; Nelumbinis Plumula] 3g | Zopiclone 7.5mg hs | Department of Pharmacy, Dongzhimen Hospital, Beijing University of Chinese Medicine | Not reported | Not reported | 4 weeks | None |
| Lai 2011 | EAHM vs. sedative-hypnotics | Diankuang Mengxing Tang | Decoction | Prunus persica (L.) Batsch [Rosaceae; Persicae Semen] 24g, Areca catechu L. [Arecaceae; Arecae Pericarpium], Cuscuta chinensis Lam. [Convolvulaceae; Cuscutae Semen] 20g, Morus alba L. [Moraceae; Mori Radicis Cortex], Rehmannia glutinosa (Gaertn.) DC. [Orobanchaceae; Rehmanniae Radix Preparata] 15g, Perilla frutescens (L.) Britton [Lamiaceae; Perillae Fructus] 12g, Bupleurum falcatum L. [Apiaceae; Bupleuri Radix], Paeonia lactiflora Pall. [Paeoniaceae; Radix Paeoniae Rubra], Cyperus rotundus L. [Cyperaceae; Cyperi Rhizoma], Pinellia ternata (Thunb.) Makino [Araceae; Pinelliae Tuber] 10g, Akebia quinata Decaisne [Lardizabalaceae; Akebiae Caulis], Citrus × aurantium f. deliciosa (Ten.) M.Hiroe [Rutaceae; Citri Unshius Pericarpium] 9g, Glycyrrhiza glabra L. [Fabaceae; Glycyrrhizae Radix et Rhizoma], Citrus × aurantium f. deliciosa (Ten.) M.Hiroe [Rutaceae; Citri Unshius Pericarpium Immaturus] 6g | Alprazolam 5mg hs | Not reported | Not reported | Not reported | 60 days | None |
| Lai 2022 | EAHM + sedative-hypnotics vs. sedative-hypnotics | Zishui Bugan Tang | Decoction | Poria cocos Wolf [Polyporaceae; Poria Sclertum Cum Pini Radix], Zizyphus jujuba Miller var. spinosa Hu ex H. F. Chou [Rhamnaceae; Zizyphi Semen] 30g, Rehmannia glutinosa (Gaertner) Liboschitz ex Steudel [Scrophulariaceae; Rehmanniae Radix Recens] 20g, Cornus officinalis Siebold & Zucc. [Cornaceae; Corni Fructus], Lycium chinense Miller [Solanaceae; Lycii Fructus], Angelica gigas Nakai [Apiaceae; Angelicae Gigantis Radix], Paeonia lactiflora Pall. [Paeoniaceae; Paeoniae Radix], Lilium lancifolium Thunb. [Liliaceae; Lilii Bulbus], Ganoderma lucidum Karsten [Polyporaceae; Ganoderma], Albizzia julibrissin Durazzini [Leguminosae; Albizziae Cortex] 15g, Conioselinum anthriscoides 'Chuanxiong' [Apiaceae; Cnidii Rhizoma], Schisandra chinensis (Turcz.) Baill. [Schisandraceae; Schisandrae Fructus] 10g, Caulis Perillae [Lamiaceae; Perilla Frutescens] 3g | Eszopiclone 3mg hs | Not reported | Not reported | Not reported | 4 weeks | None |
| Lan 2020 | EAHM vs. sedative-hypnotics | Xiaochaihu Tang | Decoction | Reynoutria multiflora (Thunb.) Moldenke [Polygonaceae; Polygoni Multiflori Caulis], Fossilia Ossis Mastodi, Ostrea gigas Thunberg [Ostreidae; Ostreae Testa] 30g, Curcuma longa L. [Zingiberaceae; Curcumae Radix], Zizyphus jujuba Miller var. spinosa Hu ex H. F. Chou [Rhamnaceae; Zizyphi Semen], Schisandra chinensis (Turcz.) Baill. [Schisandraceae; Schisandrae Fructus] 15g, Pinellia ternata (Thunb.) Makino [Araceae; Pinelliae Tuber], Bupleurum falcatum L. [Apiaceae; Bupleuri Radix], Scutellaria baicalensis Georgi [Lamiaceae; Scutellariae Radix], Codonopsis pilosula (Franch.) Nannf. [Campanulaceae; Codonopsis Pilosulae Radix], Poria cocos Wolf [Polyporaceae; Poria Sclerotium], Paeonia lactiflora Pall. [Paeoniaceae; Paeoniae Radix] 10g, Neolitsea cassia (L.) Kosterm. [Lauraceae; Cinnamomi Ramulus], Ziziphus jujuba Mill. [Rhamnaceae; Zizyphi Fructus], Glycyrrhiza glabra L. [Fabaceae; Glycyrrhizae Radix et Rhizoma] 6g | Zopiclone 7.5mg hs | Not reported | Not reported | Not reported | 2 weeks | None |
| Lei 2013 | EAHM vs. sedative-hypnotics | Liuwei Dihuang Pills and Xiaoyao San | Decoction | Dioscorea oppositifolia L. [Dioscoreaceae; Dioscoreae Rhizoma], Poria cocos Wolf [Polyporaceae; Poria Sclerotium], Ligustrum lucidum W.T.Aiton [Oleaceae; Ligustri Fructus], Paeonia lactiflora Pall. [Paeoniaceae; Paeoniae Radix], Curcuma longa L. [Zingiberaceae; Curcumae Radix], Zizyphus jujuba Miller var. spinosa Hu ex H. F. Chou [Rhamnaceae; Zizyphi Semen], Albizzia julibrissin Durazzini [Leguminosae; Albizziae Cortex] 15g, Rehmannia glutinosa (Gaertn.) DC. [Orobanchaceae; Rehmanniae Radix Preparata], Alisma plantago-aquatica subsp. orientale (Sam.) Sam. [Alismataceae; Alismatis Rhizoma] 12g, Cornus officinalis Siebold & Zucc. [Cornaceae; Corni Fructus], Paeonia × suffruticosa Andrews [Paeoniaceae; Moutan Radicis Cortex], Bupleurum falcatum L. [Apiaceae; Bupleuri Radix] 10g | Estazolam 1mg hs | Not reported | Not reported | Not reported | 14 days | None |
| Li 2015 | EAHM vs. sedative-hypnotics | Gengnian Anshen Mixture | Mixture | crystal sugar 300g, Chinemys reevesii Gray [Emydidae; Testudinis Chinemis Plastrum et Carapax] 250g, Zizyphus jujuba Miller var. spinosa Hu ex H. F. Chou [Rhamnaceae; Zizyphi Semen], Thuja orientalis Linné [Cupressaceae; Thujae Semen], Reynoutria multiflora (Thunb.) Moldenke [Polygonaceae; Polygoni Multiflori Caulis], Albizzia Julibrissin [Leguminosae; Albizziae Flos], Lilium lancifolium Thunb. [Liliaceae; Lilii Bulbus], Salvia miltiorrhiza Bunge [Lamiaceae; Salviae Miltiorrhizae Radix], Leonurus japonicus Houttuyn [Labiatae; Leonuri Herba], Lu Lu Tong [Liquidamberis Fructus], Magenetitum, Elephas maximus [Elephantidae; Fossilia Mastodi Dentis], Zhen Zhu Mu [Pteridae; Margaritifera Usta Concha] 120g, Rehmannia glutinosa (Gaertner) Liboschitz ex Steudel [Scrophulariaceae; Rehmanniae Radix Recens], Rehmannia glutinosa (Gaertn.) DC. [Orobanchaceae; Rehmanniae Radix Preparata], Cornus officinalis Siebold & Zucc. [Cornaceae; Corni Fructus], Polygonum multiflorum Thunberg [Polygonaceae; Polygoni Multiflori Radix], Eclipta prostrata Linné [Compositae; Ecliptae Herba], Ligustrum lucidum W.T.Aiton [Oleaceae; Ligustri Fructus], Anemarrhena asphodeloides Bunge [Asparagaceae; Anemarrhenae Rhizoma], Phellodendron amurense Rupr. [Rutaceae; Phellodendri Cortex], Scrophularia ningpoensis Hemsl. [Scrophulariaceae; Scrophulariae Radix], Ophiopogon japonicus (Thunb.) Ker Gawl. [Asparagaceae; Liriopis seu Ophiopogonis Tuber], Pueraria montana var. lobata (Willd.) Maesen & S.M.Almeida ex Sanjappa & Predeep [Fabaceae; Puerariae Radix], Galanthus nivalis L. [Amaryllidaceae; Lumbricus] 90g, Conioselinum anthriscoides 'Chuanxiong' [Apiaceae; Cnidii Rhizoma], Prunus persica (L.) Batsch [Rosaceae; Persicae Semen], Carthamus tinctorius Linné [Compositae; Carthami Flos], Paeonia lactiflora Pall. [Paeoniaceae; Radix Paeoniae Rubra], Paeonia × suffruticosa Andrews [Paeoniaceae; Moutan Radicis Cortex], Bupleurum falcatum L. [Apiaceae; Bupleuri Radix], Paeonia lactiflora Pall. [Paeoniaceae; Paeoniae Radix], Citrus × aurantium L. [Rutaceae; Aurantii Fructus Immaturus] 60g, Coptis chinensis Franch. [Ranunculaceae; Coptidis Rhizoma], Cinnamomum cassia Presl [Lauraceae; Cinnamomi Cortex] 30g | Estazolam 1mg hs | Not reported | Not reported | Not reported | 1 month | 3 months |
| Li 2016 | EAHM vs. sedative-hypnotics | Suanzaoren Tang | Decoction | Zizyphus jujuba Miller var. spinosa Hu ex H. F. Chou [Rhamnaceae; Zizyphi Semen] 30g, Poria cocos Wolf [Polyporaceae; Poria Sclerotium] 15g, Conioselinum anthriscoides 'Chuanxiong' [Apiaceae; Cnidii Rhizoma], Anemarrhena asphodeloides Bunge [Asparagaceae; Anemarrhenae Rhizoma], Glycyrrhiza glabra L. [Fabaceae; Glycyrrhizae Radix et Rhizoma] 10g | Estazolam 2mg hs | Not reported | Not reported | Not reported | 30 days | None |
| Li 2018 | EAHM vs. sedative-hypnotics | Qiju Dihuang Pill | Pill | Lycium chinense Miller [Solanaceae; Lycii Fructus], Chrysanthemum × morifolium (Ramat.) Hemsl. [Asteraceae; Chrysanthmi Flos], Rehmannia glutinosa (Gaertn.) DC. [Orobanchaceae; Rehmanniae Radix Preparata], Cornus officinalis Siebold & Zucc. [Cornaceae; Corni Fructus], Paeonia × suffruticosa Andrews [Paeoniaceae; Moutan Radicis Cortex], Dioscorea oppositifolia L. [Dioscoreaceae; Dioscoreae Rhizoma], Poria cocos Wolf [Polyporaceae; Poria Sclerotium], Alisma plantago-aquatica subsp. orientale (Sam.) Sam. [Alismataceae; Alismatis Rhizoma] | Estazolam 1mg qd, Losartan potassium tablet 50mg qd | Henan Wanxi Pharmaceutical Co., Ltd. | Not reported | Not reported | 4 weeks | None |
| Li 2021 | EAHM vs. oryzanol | Ganmai Dazao Tang plus Baihe Dihuang Tang | Decoction | Triticum aestivum Linné [Gramineae; Tritici Fructus Levis] 30g, Rehmannia glutinosa (Gaertner) Liboschitz ex Steudel [Scrophulariaceae; Rehmanniae Radix Recens] 20g, Lilium lancifolium Thunb. [Liliaceae; Lilii Bulbus] 15g, Glycyrrhiza glabra L. [Fabaceae; Glycyrrhizae Radix et Rhizoma] 10g, Ziziphus jujuba Mill. [Rhamnaceae; Zizyphi Fructus] 10 pieces | Oryzanol 20mg tid | Not reported | Not reported | Not reported | 14 days | None |
| Li 2022 | EAHM vs. sedative-hypnotics | Kuntai Capsule | Capsule | Rehmannia glutinosa (Gaertn.) DC. [Orobanchaceae; Rehmanniae Radix Preparata], Coptis chinensis Franch. [Ranunculaceae; Coptidis Rhizoma], Paeonia lactiflora Pall. [Paeoniaceae; Paeoniae Radix], Scutellaria baicalensis Georgi [Lamiaceae; Scutellariae Radix], Equus asinus Linne [Equidae; Asini Corii Colla], Poria cocos Wolf [Polyporaceae; Poria Sclerotium] | Estazolam 2mg hs | Guiyang Xintian Medicine Co., Ltd. | Not reported | Not reported | 4 weeks | None |
| Li(a) 2020 | EAHM vs. sedative-hypnotics | Xiaochaihu Tang | Decoction | Zizyphus jujuba Miller var. spinosa Hu ex H. F. Chou [Rhamnaceae; Zizyphi Semen], Ostrea gigas Thunberg [Ostreidae; Ostreae Testa], Fossilia Ossis Mastodi 30g, Bupleurum falcatum L. [Apiaceae; Bupleuri Radix], Poria cocos Wolf [Polyporaceae; Poria Sclertum Cum Pini Radix] 20g, Codonopsis pilosula (Franch.) Nannf. [Campanulaceae; Codonopsis Pilosulae Radix] 12g, Curcuma longa L. [Zingiberaceae; Curcumae Radix], Pinellia ternata (Thunb.) Makino [Araceae; Pinelliae Tuber] 10g, Scutellaria baicalensis Georgi [Lamiaceae; Scutellariae Radix], Glycyrrhiza glabra L. [Fabaceae; Glycyrrhizae Radix et Rhizoma] 6g | Estazolam 1mg hs | Not reported | Not reported | Not reported | 4 weeks | None |
| Li(b) 2020 | EAHM + estradiol vs. estradiol | Kuntai Capsule | Capsule | Rehmannia glutinosa (Gaertn.) DC. [Orobanchaceae; Rehmanniae Radix Preparata], Coptis chinensis Franch. [Ranunculaceae; Coptidis Rhizoma], Paeonia lactiflora Pall. [Paeoniaceae; Paeoniae Radix], Scutellaria baicalensis Georgi [Lamiaceae; Scutellariae Radix], Equus asinus Linne [Equidae; Asini Corii Colla], Poria cocos Wolf [Polyporaceae; Poria Sclerotium] | (1st-2nd week) Estradiol 1mg qd, (3rd-4th week) Estradiol 1mg qd, Estradiol dydrogesterone 10md qd | Guiyang Xintian Medicine Co., Ltd. | Not reported | Not reported | 12 weeks | None |
| Liu 2021 | EAHM vs. sedative-hypnotics | Anshen Yangxin Mixture | Mixture | xylitol 25g, Rehmannia glutinosa (Gaertn.) DC. [Orobanchaceae; Rehmanniae Radix Preparata], Poria cocos Wolf [Polyporaceae; Poria Sclerotium], Reynoutria multiflora (Thunb.) Moldenke [Polygonaceae; Polygoni Multiflori Caulis], Pelodiscus sinensis (Wiegmann) [Trionychidae; Pelodiscis Carapax] 20g, Zizyphus jujuba Miller var. spinosa Hu ex H. F. Chou [Rhamnaceae; Zizyphi Semen], Triticum aestivum Linné [Gramineae; Tritici Fructus Levis], Polygonatum sibiricum Redouté [Asparagaceae; Polygonati Rhizoma] 15g, Asparagus cochinchinensis (Lour.) Merr. [Asparagaceae; Asparagi Tuber], Ophiopogon japonicus (Thunb.) Ker Gawl. [Asparagaceae; Liriopis seu Ophiopogonis Tuber], Pseudostellaria heterophylla (Miq.) Pax [Caryophyllaceae; Pseudostellariae Radix], Salvia miltiorrhiza Bunge [Lamiaceae; Salviae Miltiorrhizae Radix], Paeonia lactiflora Pall. [Paeoniaceae; Paeoniae Radix], Ligustrum lucidum W.T.Aiton [Oleaceae; Ligustri Fructus], Cornus officinalis Siebold & Zucc. [Cornaceae; Corni Fructus], Morus alba Linné [Moraceae; Mori Fructus], Lilium lancifolium Thunb. [Liliaceae; Lilii Bulbus], Polygala senega L. [Polygalaceae; Polygalae Radix], Polygonatum odoratum (Mill.) Druce [Asparagaceae; Polygonati Odorati Rhizoma], Atractylodes lancea (Thunb.) DC. [Asteraceae; Atractylodis Rhizoma Alba], Astragalus mongholicus Bunge [Fabaceae; Astragali Radix], Equus asinus Linne [Equidae; Asini Corii Colla] 10g, Citrus × aurantium f. deliciosa (Ten.) M.Hiroe [Rutaceae; Citri Unshius Pericarpium] 5g | Estazolam 1mg tid | Not reported | Not reported | Not reported | 4 weeks | None |
| Lu 2007 | EAHM vs. oryzanol | modified Ganmai Dazao Tang | Decoction | Triticum aestivum L. [Poaceae; Tritici Fructus Levis] 40g, Gardenia jasminoides J.Ellis [Rubiaceae; Gardeniae Fructus], Rehmannia glutinosa (Gaertn.) DC. [Orobanchaceae; Rehmanniae Radix Preparata] 20g, Zizyphus jujuba Miller var. spinosa Hu ex H. F. Chou [Rhamnaceae; Zizyphi Semen] 15g, Ziziphus jujuba Mill. [Rhamnaceae; Zizyphi Fructus], Paeonia lactiflora Pall. [Paeoniaceae; Paeoniae Radix], Ostrea gigas Thunberg [Ostreidae; Ostreae Testa], Zhen Zhu Mu [Pteridae; Margaritifera Usta Concha], Curcuma longa L. [Zingiberaceae; Curcumae Radix] 10g, Glycyrrhiza glabra L. [Fabaceae; Glycyrrhizae Radix et Rhizoma] 6g | Oryzanol 20mg tid, Vitamin B3 120mg tid | Not reported | Not reported | Not reported | 30 days | None |
| Lu 2019 | EAHM vs. sedative-hypnotics | modified Guizhi Gancao Longgu Muli Tang | Decoction | Ostrea gigas Thunberg [Ostreidae; Ostreae Testa], Fossilia Ossis Mastodi 30g, Neolitsea cassia (L.) Kosterm. [Lauraceae; Cinnamomi Ramulus], Glycyrrhiza glabra L. [Fabaceae; Glycyrrhizae Radix et Rhizoma] 10g | Alprazolam 0.4mg tid | Not reported | Not reported | Not reported | 4 weeks | None |
| Luo 2021 | EAHM + antidepressants vs. antidepressants | Danzhi Xiaoyao Pill | Pill | Paeonia × suffruticosa Andrews [Paeoniaceae; Moutan Radicis Cortex], Gardenia jasminoides J.Ellis [Rubiaceae; Gardeniae Fructus], Bupleurum falcatum L. [Apiaceae; Bupleuri Radix], Paeonia lactiflora Pall. [Paeoniaceae; Paeoniae Radix], Angelica gigas Nakai [Apiaceae; Angelicae Gigantis Radix], Atractylodes lancea (Thunb.) DC. [Asteraceae; Atractylodis Rhizoma Alba], Poria cocos Wolf [Polyporaceae; Poria Sclerotium], Mentha canadensis L. [Lamiaceae; Menthae Herba], Glycyrrhiza glabra L. [Fabaceae; Glycyrrhizae Radix et Rhizoma] | Deanxit 10mg bid | Kunming Traditional Chinese Medicine Factory Co., Ltd. | Not reported | Not reported | 3 months | None |
| Ma 2018 | EAHM + sedative-hypnotics vs. sedative-hypnotics | Diaochong Anshen Tang | Decoction | Poria cocos Wolf [Polyporaceae; Poria Sclertum Cum Pini Radix] 20g, Rehmannia glutinosa (Gaertner) Liboschitz ex Steudel [Scrophulariaceae; Rehmanniae Radix Recens] 15g, Angelica gigas Nakai [Apiaceae; Angelicae Gigantis Radix], Leonurus japonicus Houttuyn [Labiatae; Leonuri Herba], Conioselinum anthriscoides 'Chuanxiong' [Apiaceae; Cnidii Rhizoma], Zizyphus jujuba Miller var. spinosa Hu ex H. F. Chou [Rhamnaceae; Zizyphi Semen], Desmanthus virgatus (Linn.) Willd. [Leguminosae; Desmanthus], Reynoutria multiflora (Thunb.) Moldenke [Polygonaceae; Polygoni Multiflori Caulis] 10g, Cyperus rotundus L. [Cyperaceae; Cyperi Rhizoma], Bupleurum falcatum L. [Apiaceae; Bupleuri Radix] 6g | Eszopiclone 2mg qd | Not reported | Not reported | Not reported | 1 month | None |
| Mahmoudi 2020 | EAHM vs. placebo EAHM | Jujube Capsule | Capsule | Zizyphus jujuba Miller var. spinosa Hu ex H. F. Chou [Rhamnaceae; Zizyphi Semen] 500mg | placebo EAHM | Laboratory of the Pharmacy faculty of Ahvaz Jundishapur University of Medical Sciences | Not reported | Not reported | 21 days | None |
| Mao 2020 | EAHM + sedative-hypnotics vs. sedative-hypnotics | modified Huanglian Ejiao Tang | Decoction | Fossilia Ossis Mastodi, Ostrea gigas Thunberg [Ostreidae; Ostreae Testa] 30g, Zizyphus jujuba Miller var. spinosa Hu ex H. F. Chou [Rhamnaceae; Zizyphi Semen] 20g, Equus asinus Linne [Equidae; Asini Corii Colla], Paeonia lactiflora Pall. [Paeoniaceae; Paeoniae Radix] 15g, Coptis chinensis Franch. [Ranunculaceae; Coptidis Rhizoma], Cornus officinalis Siebold & Zucc. [Cornaceae; Corni Fructus] 12g, Astragalus mongholicus Bunge [Fabaceae; Astragali Radix], Lycium chinense Miller [Solanaceae; Lycii Fructus] 10g, Gallus domesticus [Phasianidae; Galli Vitellus] 2 pieces | Alprazolam 0.4 qd | Not reported | Not reported | Not reported | 1 month | None |
| Mi 2022 | EAHM vs. placebo EAHM | Zishui Bugan Tang | Decoction | Poria cocos Wolf [Polyporaceae; Poria Sclertum Cum Pini Radix], Zizyphus jujuba Miller var. spinosa Hu ex H. F. Chou [Rhamnaceae; Zizyphi Semen] 30g, Rehmannia glutinosa (Gaertner) Liboschitz ex Steudel [Scrophulariaceae; Rehmanniae Radix Recens] 20g, Cornus officinalis Siebold & Zucc. [Cornaceae; Corni Fructus], Lycium chinense Miller [Solanaceae; Lycii Fructus], Angelica gigas Nakai [Apiaceae; Angelicae Gigantis Radix], Paeonia lactiflora Pall. [Paeoniaceae; Paeoniae Radix], Lilium lancifolium Thunb. [Liliaceae; Lilii Bulbus], Ganoderma lucidum Karsten [Polyporaceae; Ganoderma], Albizzia julibrissin Durazzini [Leguminosae; Albizziae Cortex] 15g, Conioselinum anthriscoides 'Chuanxiong' [Apiaceae; Cnidii Rhizoma], Schisandra chinensis (Turcz.) Baill. [Schisandraceae; Schisandrae Fructus] 10g, Caulis Perillae [Lamiaceae; Perilla Frutescens] 3g | placebo EAHM | Beijing Donghuayuan Medical Equipment Co., Ltd. | Not reported | Not reported | 3 weeks | None |
| Pan 2022 | EAHM + sedative-hypnotics vs. sedative-hypnotics | modified Chaihu Jia Longgu Muli Tang | Decoction | Fossilia Ossis Mastodi, Ostrea gigas Thunberg [Ostreidae; Ostreae Testa] 30g, Bupleurum falcatum L. [Apiaceae; Bupleuri Radix], Cyperus rotundus L. [Cyperaceae; Cyperi Rhizoma], Citrus × aurantium f. deliciosa (Ten.) M.Hiroe [Rutaceae; Citri Unshius Pericarpium], Poria cocos Wolf [Polyporaceae; Poria Sclertum Cum Pini Radix] 12g, Citrus × aurantium f. deliciosa (Ten.) M.Hiroe [Rutaceae; Citri Unshius Pericarpium Immaturus], Citrus trifoliata L. [Rutaceae; Ponciri Fructus Immaturus], Curcuma longa L. [Zingiberaceae; Curcumae Radix], Paeonia lactiflora Pall. [Paeoniaceae; Paeoniae Radix] 10g, Polygala senega L. [Polygalaceae; Polygalae Radix], Glycyrrhiza glabra L. [Fabaceae; Glycyrrhizae Radix et Rhizoma] 6g | Estazolam 2mg hs | Not reported | Not reported | Not reported | 6 weeks | None |
| Pang 2019 | EAHM + sedative-hypnotics vs. sedative-hypnotics | Anshen Pill | Pill | Zizyphus jujuba Miller var. spinosa Hu ex H. F. Chou [Rhamnaceae; Zizyphi Semen] 15g, Polygala senega L. [Polygalaceae; Polygalae Radix], Ostrea gigas Thunberg [Ostreidae; Ostreae Testa], Poria cocos Wolf [Polyporaceae; Poria Sclertum Cum Pini Radix], Angelica gigas Nakai [Apiaceae; Angelicae Gigantis Radix], Albizzia Julibrissin [Leguminosae; Albizziae Flos], Gardenia jasminoides J.Ellis [Rubiaceae; Gardeniae Fructus], Albizzia julibrissin Durazzini [Leguminosae; Albizziae Cortex], Conioselinum anthriscoides 'Chuanxiong' [Apiaceae; Cnidii Rhizoma], Reynoutria multiflora (Thunb.) Moldenke [Polygonaceae; Polygoni Multiflori Caulis], Anemarrhena asphodeloides Bunge [Asparagaceae; Anemarrhenae Rhizoma], Curcuma longa L. [Zingiberaceae; Curcumae Radix], Elephas maximus [Elephantidae; Fossilia Mastodi Dentis], Lilium lancifolium Thunb. [Liliaceae; Lilii Bulbus] 10g, Epimedium sagittatum (Siebold & Zucc.) Maxim. [Berberidaceae; Epimedii Herba], Fossilia Ossis Mastodi 6g | Estazolam 1-2mg hs | Not reported | Not reported | Not reported | 1 month | None |
| Pu 2019 | EAHM + sedative-hypnotics vs. sedative-hypnotics | modified Buxin Xiaoyao Yin | Decoction | Paeonia lactiflora Pall. [Paeoniaceae; Paeoniae Radix], Lilium lancifolium Thunb. [Liliaceae; Lilii Bulbus], Zizyphus jujuba Miller var. spinosa Hu ex H. F. Chou [Rhamnaceae; Zizyphi Semen], 20g, Bupleurum falcatum L. [Apiaceae; Bupleuri Radix], Haematitum, Albizzia julibrissin Durazzini [Leguminosae; Albizziae Cortex] 15g, Angelica gigas Nakai [Apiaceae; Angelicae Gigantis Radix] 12g, Atractylodes lancea (Thunb.) DC. [Asteraceae; Atractylodis Rhizoma Alba], Inula japonica Thunb. [Asteraceae; Inulae Flos], Lophatherum gracile Bronghiart [Gramineae; Lophatheri Herba], Glycine max Merrill [Leguminosae; Glycine Semen Preparata], Poria cocos Wolf [Polyporaceae; Poria Sclerotium], Triticum aestivum Linné [Gramineae; Tritici Fructus Levis], Scrophularia ningpoensis Hemsl. [Scrophulariaceae; Scrophulariae Radix] 10g, Glycyrrhiza glabra L. [Fabaceae; Glycyrrhizae Radix et Rhizoma] 6g | Eszopiclone 3mg hs | Not reported | Not reported | Not reported | 4 weeks | None |
| Qiao 2021 | EAHM + sedative-hypnotics vs. sedative-hypnotics | Tianwang Buxin Pill plus Jiaotai Pill | Decoction | Rehmannia glutinosa (Gaertner) Liboschitz ex Steudel [Scrophulariaceae; Rehmanniae Radix Recens] 45g, Zizyphus jujuba Miller var. spinosa Hu ex H. F. Chou [Rhamnaceae; Zizyphi Semen] 30g, Thuja orientalis Linné [Cupressaceae; Thujae Semen], Coptis chinensis Franch. [Ranunculaceae; Coptidis Rhizoma] 25g, Angelica gigas Nakai [Apiaceae; Angelicae Gigantis Radix], Schisandra chinensis (Turcz.) Baill. [Schisandraceae; Schisandrae Fructus], Ophiopogon japonicus (Thunb.) Ker Gawl. [Asparagaceae; Liriopis seu Ophiopogonis Tuber], Asparagus cochinchinensis (Lour.) Merr. [Asparagaceae; Asparagi Tuber] 20g, Panax ginseng C.A.Mey. [Araliaceae; Ginseng Radix], Poria cocos Wolf [Polyporaceae; Poria Sclerotium], Scrophularia ningpoensis Hemsl. [Scrophulariaceae; Scrophulariae Radix], Salvia miltiorrhiza Bunge [Lamiaceae; Salviae Miltiorrhizae Radix], Platycodon grandiflorus (Jacq.) A.DC. [Campanulaceae; Platycodonis Radix], Polygala senega L. [Polygalaceae; Polygalae Radix], Cinnamomum cassia Presl [Lauraceae; Cinnamomi Cortex] 15g | Estradiol valerate/estradiol cyproterone 1C qd, Metoprolol tartrate 50mg qd, Estazolam 2mg hs | Traditional Chinese Medicine Pharmacy of the Second Affiliated Hospital of Heilongjiang University of Traditional Chinese Medicine | Not reported | Not reported | 4 weeks | 2 weeks |
| Qin 2023 | EAHM + sedative-hypnotics vs. sedative-hypnotics | modified Wendan Tang plus Chaihu Shugan San | Decoction | Paeonia lactiflora Pall. [Paeoniaceae; Paeoniae Radix] 15g, Citrus × aurantium f. deliciosa (Ten.) M.Hiroe [Rutaceae; Citri Unshius Pericarpium] 12g, Poria cocos Wolf [Polyporaceae; Poria Sclerotium], Pinellia ternata (Thunb.) Makino [Araceae; Pinelliae Tuber], Phyllostachys nigra Munro var. henonsis Stapf [Gramineae; Phyllostachyos Caulis in Taeniam], Citrus trifoliata L. [Rutaceae; Ponciri Fructus Immaturus] 9g, Bupleurum falcatum L. [Apiaceae; Bupleuri Radix], Conioselinum anthriscoides 'Chuanxiong' [Apiaceae; Cnidii Rhizoma], Cyperus rotundus L. [Cyperaceae; Cyperi Rhizoma], Glycyrrhiza glabra L. [Fabaceae; Glycyrrhizae Radix et Rhizoma] 6g | Estazolam 1mg hs | Not reported | Not reported | Not reported | 2 months | None |
| Rui 2017 | EAHM + sedative-hypnotics vs. sedative-hypnotics | modified Qinggan Xiexin Tang | Decoction | Bupleurum falcatum L. [Apiaceae; Bupleuri Radix], Gardenia jasminoides J.Ellis [Rubiaceae; Gardeniae Fructus], Coptis chinensis Franch. [Ranunculaceae; Coptidis Rhizoma], Scutellaria baicalensis Georgi [Lamiaceae; Scutellariae Radix], Lilium lancifolium Thunb. [Liliaceae; Lilii Bulbus], Rehmannia glutinosa (Gaertner) Liboschitz ex Steudel [Scrophulariaceae; Rehmanniae Radix Recens], Anemarrhena asphodeloides Bunge [Asparagaceae; Anemarrhenae Rhizoma], Pollen | Zopiclone 7.5-15mg hs | Not reported | Not reported | Not reported | 4 weeks | None |
| Shan 2020 | EAHM + sedative-hypnotics vs. sedative-hypnotics | modified Yanghe Tang | Decoction | Rehmannia glutinosa (Gaertn.) DC. [Orobanchaceae; Rehmanniae Radix Preparata], Epimedium sagittatum (Siebold & Zucc.) Maxim. [Berberidaceae; Epimedii Herba] 30g, Aconitum carmichaelii Debeaux [Ranunculaceae; Aconiti Lateralis Radix Preparata] 15g, Sinapis alba L. [Brassicaceae; Sinapis Semen Alba] 6g, Equus asinus Linne [Equidae; Asini Corii Colla], Glycyrrhiza glabra L. [Fabaceae; Glycyrrhizae Radix et Rhizoma] 5g, Cinnamomum cassia Presl [Lauraceae; Cinnamomi Cortex], Neolitsea cassia (L.) Kosterm. [Lauraceae; Cinnamomi Ramulus] 3g | Estazolam 2mg qd | Not reported | Not reported | Not reported | 4 weeks | None |
| Shen 2018 | EAHM + sedative-hypnotics vs. sedative-hypnotics | Shugan Jianpi Tang | Decoction | Poria cocos Wolf [Polyporaceae; Poria Sclerotium], Rehmannia glutinosa (Gaertner) Liboschitz ex Steudel [Scrophulariaceae; Rehmanniae Radix Recens], Lilium lancifolium Thunb. [Liliaceae; Lilii Bulbus], Codonopsis pilosula (Franch.) Nannf. [Campanulaceae; Codonopsis Pilosulae Radix], Fructus Akebiae [Akebia; Akebia Fruit], Albizzia julibrissin Durazzini [Leguminosae; Albizziae Cortex] 15g, Bupleurum falcatum L. [Apiaceae; Bupleuri Radix], Paeonia lactiflora Pall. [Paeoniaceae; Paeoniae Radix], Angelica gigas Nakai [Apiaceae; Angelicae Gigantis Radix], Atractylodes lancea (Thunb.) DC. [Asteraceae; Atractylodis Rhizoma Alba], Conioselinum anthriscoides 'Chuanxiong' [Apiaceae; Cnidii Rhizoma], Curcuma longa L. [Zingiberaceae; Curcumae Radix], Citrus × aurantium L. [Rutaceae; Aurantii Fructus Immaturus] 10g, Glycyrrhiza glabra L. [Fabaceae; Glycyrrhizae Radix et Rhizoma], Polygala senega L. [Polygalaceae; Polygalae Radix], Prunus mume [Rosaceae; Mume Flos] 6g | Alprazolam 0.4mg qd | Not reported | Not reported | Not reported | 4 weeks | None |
| Shi 2018 | EAHM + sedative-hypnotics vs. sedative-hypnotics | Zishen Jieyu Ningxin Recipe Fang | Decoction | Reynoutria multiflora (Thunb.) Moldenke [Polygonaceae; Polygoni Multiflori Caulis] 30g, Albizzia julibrissin Durazzini [Leguminosae; Albizziae Cortex] 20g, Ligustrum lucidum W.T.Aiton [Oleaceae; Ligustri Fructus], Salvia miltiorrhiza Bunge [Lamiaceae; Salviae Miltiorrhizae Radix], Zizyphus jujuba Miller var. spinosa Hu ex H. F. Chou [Rhamnaceae; Zizyphi Semen] 15g, Cornus officinalis Siebold & Zucc. [Cornaceae; Corni Fructus], Angelica gigas Nakai [Apiaceae; Angelicae Gigantis Radix], Rehmannia glutinosa (Gaertn.) DC. [Orobanchaceae; Rehmanniae Radix Preparata], Paeonia lactiflora Pall. [Paeoniaceae; Radix Paeoniae Rubra], Gardenia jasminoides J.Ellis [Rubiaceae; Gardeniae Fructus], Paeonia lactiflora Pall. [Paeoniaceae; Paeoniae Radix], Paeonia × suffruticosa Andrews [Paeoniaceae; Moutan Radicis Cortex] 10g | Estazolam 2-4mg hs | Not reported | Not reported | Not reported | 2 months | None |
| Su 2021 | EAHM + sedative-hypnotics vs. sedative-hypnotics | modified Erxian Tang | Decoction | Curculigo orchioides Gaertner [Amarylidaceae; Curculiginis Rhizoma], Fossilia Ossis Mastodi, Ostrea gigas Thunberg [Ostreidae; Ostreae Testa] 30g, Epimedium sagittatum (Siebold & Zucc.) Maxim. [Berberidaceae; Epimedii Herba] 15g, Morinda officinalis How [Rubiaceae; Morindae Radix], Zhīmíng, Phellodendron amurense Rupr. [Rutaceae; Phellodendri Cortex], Angelica gigas Nakai [Apiaceae; Angelicae Gigantis Radix], Zizyphus jujuba Miller var. spinosa Hu ex H. F. Chou [Rhamnaceae; Zizyphi Semen], Albizzia julibrissin Durazzini [Leguminosae; Albizziae Cortex] 10g | Alprazolam 0.4mg hs | Not reported | Not reported | Not reported | 1 month | None |
| Sun 2016 | EAHM vs. sedative-hypnotics | modified Wendan Tang | Decoction | Poria cocos Wolf [Polyporaceae; Poria Sclerotium], Zhen Zhu Mu [Pteridae; Margaritifera Usta Concha] 30g, Angelica gigas Nakai [Apiaceae; Angelicae Gigantis Radix], Zizyphus jujuba Miller var. spinosa Hu ex H. F. Chou [Rhamnaceae; Zizyphi Semen], Citrus trifoliata L. [Rutaceae; Ponciri Fructus Immaturus], Magnolia officinalis Rehder & E.H.Wilson [Magnoliaceae; Magnoliae Cortex], Arnebia guttata Bunge [Boraginaceae; Lithospermi Radix] 15g, Alisma plantago-aquatica subsp. orientale (Sam.) Sam. [Alismataceae; Alismatis Rhizoma] 12g, Phyllostachys nigra Munro var. henonsis Stapf [Gramineae; Phyllostachyos Caulis in Taeniam], Ligustrum lucidum W.T.Aiton [Oleaceae; Ligustri Fructus], Paeonia × suffruticosa Andrews [Paeoniaceae; Moutan Radicis Cortex] 8g, Coptis chinensis Franch. [Ranunculaceae; Coptidis Rhizoma], Gardenia jasminoides J.Ellis [Rubiaceae; Gardeniae Fructus], Glycyrrhiza glabra L. [Fabaceae; Glycyrrhizae Radix et Rhizoma] 6g | Estazolam 1mg hs | Not reported | Not reported | Not reported | 30 days | None |
| Sun 2020 | EAHM vs. sedative-hypnotics | Suanzaoren Tang | Decoction | Zizyphus jujuba Miller var. spinosa Hu ex H. F. Chou [Rhamnaceae; Zizyphi Semen] 30g, Poria cocos Wolf [Polyporaceae; Poria Sclerotium], Paeonia lactiflora Pall. [Paeoniaceae; Paeoniae Radix] 15g, Conioselinum anthriscoides 'Chuanxiong' [Apiaceae; Cnidii Rhizoma], Anemarrhena asphodeloides Bunge [Asparagaceae; Anemarrhenae Rhizoma], Astragalus mongholicus Bunge [Fabaceae; Astragali Radix] 10g, Glycyrrhiza glabra L. [Fabaceae; Glycyrrhizae Radix et Rhizoma] 5g | Estazolam 1-2mg hs | Not reported | Not reported | Not reported | 3 weeks | None |
| Wang 2014 | EAHM vs. sedative-hypnotics | Suanzaoren Tang | Decoction | Anemarrhena asphodeloides Bunge [Asparagaceae; Anemarrhenae Rhizoma], Poria cocos Wolf [Polyporaceae; Poria Sclerotium], Ophiopogon japonicus (Thunb.) Ker Gawl. [Asparagaceae; Liriopis seu Ophiopogonis Tuber] 15g, Conioselinum anthriscoides 'Chuanxiong' [Apiaceae; Cnidii Rhizoma] 10g, Glycyrrhiza glabra L. [Fabaceae; Glycyrrhizae Radix et Rhizoma] 6g, Schisandra chinensis (Turcz.) Baill. [Schisandraceae; Schisandrae Fructus] 5g | Diazepam 5-10mg hs, Vitamin E 100mg tid, Oryzanol 20mg tid | Not reported | Not reported | Not reported | 12 days | None |
| Wang 2023 | EAHM vs. estradiol | modified Liuwei Dihuang Tang and Xiaoyao San | Decoction | Rehmannia glutinosa (Gaertn.) DC. [Orobanchaceae; Rehmanniae Radix Preparata], Dioscorea oppositifolia L. [Dioscoreaceae; Dioscoreae Rhizoma], Paeonia × suffruticosa Andrews [Paeoniaceae; Moutan Radicis Cortex], Poria cocos Wolf [Polyporaceae; Poria Sclerotium], Paeonia lactiflora Pall. [Paeoniaceae; Paeoniae Radix], Atractylodes lancea (Thunb.) DC. [Asteraceae; Atractylodis Rhizoma Alba] 15g, Cornus officinalis Siebold & Zucc. [Cornaceae; Corni Fructus] 12g, Bupleurum falcatum L. [Apiaceae; Bupleuri Radix], Angelica gigas Nakai [Apiaceae; Angelicae Gigantis Radix], Glycyrrhiza glabra L. [Fabaceae; Glycyrrhizae Radix et Rhizoma] 10g, Alisma plantago-aquatica subsp. orientale (Sam.) Sam. [Alismataceae; Alismatis Rhizoma] 9g | (5th-10th day of menstruation) Estradiol valerate 1mg qd (11th-21th day of menstruation) Estradiol valerate 1mg qd + Dydrogesterone 20mg qd | Not reported | Not reported | Not reported | 3 months | None |
| Wang(a) 2018 | EAHM + antidepressants vs. antidepressants | Qingxin Zhengan Tang | Decoction | Zizyphus jujuba Miller var. spinosa Hu ex H. F. Chou [Rhamnaceae; Zizyphi Semen] 30g, Fossilia Ossis Mastodi, Ostrea gigas Thunberg [Ostreidae; Ostreae Testa], Angelica gigas Nakai [Apiaceae; Angelicae Gigantis Radix], Poria cocos Wolf [Polyporaceae; Poria Sclerotium] 20g, Coptis chinensis Franch. [Ranunculaceae; Coptidis Rhizoma], Scutellaria baicalensis Georgi [Lamiaceae; Scutellariae Radix], Gardenia jasminoides J.Ellis [Rubiaceae; Gardeniae Fructus], Glycine max Merrill [Leguminosae; Glycine Semen Preparata], Zhen Zhu Mu [Pteridae; Margaritifera Usta Concha], Rehmannia glutinosa (Gaertner) Liboschitz ex Steudel [Scrophulariaceae; Rehmanniae Radix Recens], Acorus gramineus Aiton [Acoraceae; Acori Graminei Rhizoma], Polygala senega L. [Polygalaceae; Polygalae Radix], Poria cocos Wolf [Polyporaceae; Poria Sclertum Cum Pini Radix] 12g | Deanxit (Dosage was not listed) | Not reported | Not reported | Not reported | 30 days | None |
| Wang(a) 2021 | EAHM vs. sedative-hypnotics | Chaihu Jia Longgu Muli Tang | Decoction | Codonopsis pilosula (Franch.) Nannf. [Campanulaceae; Codonopsis Pilosulae Radix] 20g, Fossilia Ossis Mastodi, Ostrea gigas Thunberg [Ostreidae; Ostreae Testa] 18g, Bupleurum falcatum L. [Apiaceae; Bupleuri Radix] 12g, Pinellia ternata (Thunb.) Makino [Araceae; Pinelliae Tuber] 10g, Neolitsea cassia (L.) Kosterm. [Lauraceae; Cinnamomi Ramulus] 8g, Scutellaria baicalensis Georgi [Lamiaceae; Scutellariae Radix], Poria cocos Wolf [Polyporaceae; Poria Sclerotium] 6g, Zingiber officinale Roscoe [Zingiberaceae; Zingiberis Rhizoma Recens], Rheum officinale Baill. [Polygonaceae; Rhei Radix et Rhizoma] 4g | Estazolam 0.2mg qd | Not reported | Not reported | Not reported | 30 days | None |
| Wang(a) 2022 | EAHM vs. sedative-hypnotics | Ziyin Xiexin Tang | Decoction | Thuja orientalis Linné [Cupressaceae; Thujae Semen], Ostrea gigas Thunberg [Ostreidae; Ostreae Testa], Fossilia Ossis Mastodi, Zizyphus jujuba Miller var. spinosa Hu ex H. F. Chou [Rhamnaceae; Zizyphi Semen] 30g, Ophiopogon japonicus (Thunb.) Ker Gawl. [Asparagaceae; Liriopis seu Ophiopogonis Tuber], Scrophularia ningpoensis Hemsl. [Scrophulariaceae; Scrophulariae Radix], Rehmannia glutinosa (Gaertner) Liboschitz ex Steudel [Scrophulariaceae; Rehmanniae Radix Recens], Achyranthes bidentata Blume [Amaranthaceae; Achyranthis Radix] 15g, Angelica gigas Nakai [Apiaceae; Angelicae Gigantis Radix], Coptis chinensis Franch. [Ranunculaceae; Coptidis Rhizoma], Poria cocos Wolf [Polyporaceae; Poria Sclertum Cum Pini Radix] 12g, Cinnamomum cassia Presl [Lauraceae; Cinnamomi Cortex] 2g | Zolpidem tartrate 10mg hs | Not reported | Not reported | Not reported | 4 weeks | 4 weeks |
| Wang(b) 2018 | EAHM vs. sedative-hypnotics | modified Ganmai Dazao Tang | Decoction | Triticum aestivum Linné [Gramineae; Tritici Fructus Levis] 18g, Zizyphus jujuba Miller var. spinosa Hu ex H. F. Chou [Rhamnaceae; Zizyphi Semen], Cornus officinalis Siebold & Zucc. [Cornaceae; Corni Fructus], Rehmannia glutinosa (Gaertner) Liboschitz ex Steudel [Scrophulariaceae; Rehmanniae Radix Recens], Rehmannia glutinosa (Gaertn.) DC. [Orobanchaceae; Rehmanniae Radix Preparata] 15g, Glycyrrhiza glabra L. [Fabaceae; Glycyrrhizae Radix et Rhizoma], Coptis chinensis Franch. [Ranunculaceae; Coptidis Rhizoma] 12g, Cinnamomum cassia Presl [Lauraceae; Cinnamomi Cortex] 6g | Estazolam 1mg hs, Oryzanol 20mg tid | Not reported | Not reported | Not reported | 3 months | None |
| Wang(b) 2021 | EAHM + sedative-hypnotics vs. sedative-hypnotics | modified Yiganxue Suanzaoren Tang | Decoction | Zizyphus jujuba Miller var. spinosa Hu ex H. F. Chou [Rhamnaceae; Zizyphi Semen] 20g, Conioselinum anthriscoides 'Chuanxiong' [Apiaceae; Cnidii Rhizoma], Bupleurum falcatum L. [Apiaceae; Bupleuri Radix], Ophiopogon japonicus (Thunb.) Ker Gawl. [Asparagaceae; Liriopis seu Ophiopogonis Tuber], Angelica gigas Nakai [Apiaceae; Angelicae Gigantis Radix] 15g, Poria cocos Wolf [Polyporaceae; Poria Sclerotium], Pinellia ternata (Thunb.) Makino [Araceae; Pinelliae Tuber] 12g, Anemarrhena asphodeloides Bunge [Asparagaceae; Anemarrhenae Rhizoma] 10g, Schisandra chinensis (Turcz.) Baill. [Schisandraceae; Schisandrae Fructus], Glycyrrhiza glabra L. [Fabaceae; Glycyrrhizae Radix et Rhizoma] 6g | Estazolam 1mg qd | Department of Traditional Chinese Medicine of the First Affiliated Hospital of Anhui University of Traditional Chinese Medicine | Not reported | Not reported | 2 weeks | None |
| Wang(b) 2022 | EAHM + sedative-hypnotics vs. sedative-hypnotics | modified Chaihu Jia Longgu Muli Tang | Decoction | Fossilia Ossis Mastodi, Ostrea gigas Thunberg [Ostreidae; Ostreae Testa] 30g, Cuscuta chinensis Lam. [Convolvulaceae; Cuscutae Semen], Dioscorea oppositifolia L. [Dioscoreaceae; Dioscoreae Rhizoma], Zizyphus jujuba Miller var. spinosa Hu ex H. F. Chou [Rhamnaceae; Zizyphi Semen] 20g, Bupleurum falcatum L. [Apiaceae; Bupleuri Radix], Albizzia julibrissin Durazzini [Leguminosae; Albizziae Cortex], Curcuma longa L. [Zingiberaceae; Curcumae Radix], Reynoutria multiflora (Thunb.) Moldenke [Polygonaceae; Polygoni Multiflori Caulis] 15g, Neolitsea cassia (L.) Kosterm. [Lauraceae; Cinnamomi Ramulus], Poria cocos Wolf [Polyporaceae; Poria Sclerotium] 10g, Conioselinum anthriscoides 'Chuanxiong' [Apiaceae; Cnidii Rhizoma] 9g, Glycyrrhiza glabra L. [Fabaceae; Glycyrrhizae Radix et Rhizoma] 6g | Estazolam 1mg hs | Not reported | Not reported | Not reported | 8 weeks | None |
| Wei 2020 | EAHM vs. sedative-hypnotics | modified Chaihu Guizhi Longgu Muli Tang | Decoction | Fossilia Ossis Mastodi, Ostrea gigas Thunberg [Ostreidae; Ostreae Testa] 30g, Rehmannia glutinosa (Gaertner) Liboschitz ex Steudel [Scrophulariaceae; Rehmanniae Radix Recens], Dioscorea oppositifolia L. [Dioscoreaceae; Dioscoreae Rhizoma] 15g, Bupleurum falcatum L. [Apiaceae; Bupleuri Radix], Scutellaria baicalensis Georgi [Lamiaceae; Scutellariae Radix], Curcuma longa L. [Zingiberaceae; Curcumae Radix] 12g, Neolitsea cassia (L.) Kosterm. [Lauraceae; Cinnamomi Ramulus], Pinellia ternata (Thunb.) Makino [Araceae; Pinelliae Tuber] 9g | Estazolam 1mg qd | Not reported | Not reported | Not reported | 4 weeks | None |
| Weng 2016 | EAHM vs. sedative-hypnotics | Suanzao Baihe Tang | Decoction | Fossilia Ossis Mastodi, Ostrea gigas Thunberg [Ostreidae; Ostreae Testa] 30g, Lilium lancifolium Thunb. [Liliaceae; Lilii Bulbus] 25g, Zizyphus jujuba Miller var. spinosa Hu ex H. F. Chou [Rhamnaceae; Zizyphi Semen], Achyranthes bidentata Blume [Amaranthaceae; Achyranthis Radix] 15g, Paeonia lactiflora Pall. [Paeoniaceae; Paeoniae Radix] 12g, Ophiopogon japonicus (Thunb.) Ker Gawl. [Asparagaceae; Liriopis seu Ophiopogonis Tuber], Anemarrhena asphodeloides Bunge [Asparagaceae; Anemarrhenae Rhizoma] 10g, Glycyrrhiza glabra L. [Fabaceae; Glycyrrhizae Radix et Rhizoma] 8g | Estazolam 1-2mg hs | Not reported | Not reported | Not reported | 6 weeks | 1 month |
| Xing 2018 | EAHM vs. sedative-hypnotics | modified Yokukansan | Decoction | Reynoutria multiflora (Thunb.) Moldenke [Polygonaceae; Polygoni Multiflori Caulis] 30g, Zizyphus jujuba Miller var. spinosa Hu ex H. F. Chou [Rhamnaceae; Zizyphi Semen] 18g, Uncaria rhynchophylla (Miq.) Miq. [Rubiaceae; Uncariae Ramulus cum Uncus], Albizzia julibrissin Durazzini [Leguminosae; Albizziae Cortex], Poria cocos Wolf [Polyporaceae; Poria Sclertum Cum Pini Radix] 15g, Atractylodes lancea (Thunb.) DC. [Asteraceae; Atractylodis Rhizoma Alba], Conioselinum anthriscoides 'Chuanxiong' [Apiaceae; Cnidii Rhizoma], Angelica gigas Nakai [Apiaceae; Angelicae Gigantis Radix] 12g, Lycium chinense Miller [Solanaceae; Lycii Fructus] 10g, Bupleurum falcatum L. [Apiaceae; Bupleuri Radix], Glycyrrhiza glabra L. [Fabaceae; Glycyrrhizae Radix et Rhizoma] 6g | Estazolam 2-4mg hs | Not reported | Not reported | Not reported | 30 days | 20 days |
| Xu 2023 | EAHM vs. sedative-hypnotics | NR | Decoction | Coptis chinensis Franch. [Ranunculaceae; Coptidis Rhizoma] 6g, Cinnamomum cassia Presl [Lauraceae; Cinnamomi Cortex] 2g, Zizyphus jujuba Miller var. spinosa Hu ex H. F. Chou [Rhamnaceae; Zizyphi Semen], Ganoderma lucidum Karsten [Polyporaceae; Ganoderma], Bupleurum falcatum L. [Apiaceae; Bupleuri Radix], Corydalis ternata Nakai [Papaveraceae; Corydalis Tuber], Poria cocos Wolf [Polyporaceae; Poria Sclertum Cum Pini Radix] 10g | Estazolam 1mg qd | Not reported | Not reported | Not reported | 12 weeks | None |
| Yao 2014 | EAHM + losartan potassium vs. losartan potassium | Guide Baihe Tang | Decoction | Zhen Zhu Mu [Pteridae; Margaritifera Usta Concha], Ostrea gigas Thunberg [Ostreidae; Ostreae Testa] 30g, Chinemys reevesii Gray [Emydidae; Testudinis Chinemis Plastrum et Carapax], Rehmannia glutinosa (Gaertner) Liboschitz ex Steudel [Scrophulariaceae; Rehmanniae Radix Recens], Salvia miltiorrhiza Bunge [Lamiaceae; Salviae Miltiorrhizae Radix], Reynoutria multiflora (Thunb.) Moldenke [Polygonaceae; Polygoni Multiflori Caulis] 20g, Lilium lancifolium Thunb. [Liliaceae; Lilii Bulbus], Poria cocos Wolf [Polyporaceae; Poria Sclertum Cum Pini Radix] 15g, Zizyphus jujuba Miller var. spinosa Hu ex H. F. Chou [Rhamnaceae; Zizyphi Semen] 10g | Losartan Potassium Tablet 50mg qd | Not reported | Not reported | Not reported | 8 weeks | None |
| Ye 2015 | EAHM vs. sedative-hypnotics | modified Jiayi Guizang Tang | Decoction | Zhen Zhu Mu [Pteridae; Margaritifera Usta Concha], Fossilia Ossis Mastodi, Salvia miltiorrhiza Bunge [Lamiaceae; Salviae Miltiorrhizae Radix], Albizzia Julibrissin [Leguminosae; Albizziae Flos], Reynoutria multiflora (Thunb.) Moldenke [Polygonaceae; Polygoni Multiflori Caulis] 30g, Curculigo orchioides Gaertner [Amarylidaceae; Curculiginis Rhizoma], Epimedium sagittatum (Siebold & Zucc.) Maxim. [Berberidaceae; Epimedii Herba], Paeonia lactiflora Pall. [Paeoniaceae; Paeoniae Radix], Thuja orientalis Linné [Cupressaceae; Thujae Semen], Ziziphus jujuba Mill. [Rhamnaceae; Zizyphi Fructus] 15g, Rehmannia glutinosa (Gaertner) Liboschitz ex Steudel [Scrophulariaceae; Rehmanniae Radix Recens] 12g, Angelica gigas Nakai [Apiaceae; Angelicae Gigantis Radix], Bupleurum falcatum L. [Apiaceae; Bupleuri Radix] 10g, Mentha canadensis L. [Lamiaceae; Menthae Herba] 8g, Aquilaria malaccensis Lam. [Thymelaeaceae; Aquilariae Lignum] 6g | Zolpidem tartrate 10mg hs | Not reported | Not reported | Not reported | 4 weeks | None |
| You 2022 | EAHM + sedative-hypnotics vs. sedative-hypnotics | Zigui Diaogeng Anshen Tang | Decoction | Rehmannia glutinosa (Gaertner) Liboschitz ex Steudel [Scrophulariaceae; Rehmanniae Radix Recens], Dioscorea oppositifolia L. [Dioscoreaceae; Dioscoreae Rhizoma], Lilium lancifolium Thunb. [Liliaceae; Lilii Bulbus], Elephas maximus [Elephantidae; Fossilia Mastodi Dentis], Reynoutria multiflora (Thunb.) Moldenke [Polygonaceae; Polygoni Multiflori Caulis] 15g, Cornus officinalis Siebold & Zucc. [Cornaceae; Corni Fructus], Zizyphus jujuba Miller var. spinosa Hu ex H. F. Chou [Rhamnaceae; Zizyphi Semen], Conioselinum anthriscoides 'Chuanxiong' [Apiaceae; Cnidii Rhizoma], Morus alba Linné [Moraceae; Mori Fructus], Zhen Zhu Mu [Pteridae; Margaritifera Usta Concha], Anemarrhena asphodeloides Bunge [Asparagaceae; Anemarrhenae Rhizoma], Albizzia julibrissin Durazzini [Leguminosae; Albizziae Cortex], Poria cocos Wolf [Polyporaceae; Poria Sclertum Cum Pini Radix] 10g, Glycyrrhiza glabra L. [Fabaceae; Glycyrrhizae Radix et Rhizoma] 5g | Estradiol valerate 0.6mg qd, Estazolam 0.4mg hs | Traditional Chinese Medicine Pharmacy of the Maternal and Child Health Hospital of Tonglu County, Zhejiang Province | Not reported | Not reported | 8 weeks | None |
| Zeng 2022 | EAHM vs. placebo EAHM | Jiaotai Pill | Granule | Coptis chinensis Franch. [Ranunculaceae; Coptidis Rhizoma], Cinnamomum cassia Presl [Lauraceae; Cinnamomi Cortex] | placebo EAHM | Not reported | Not reported | Not reported | 1 week | None |
| Zhang 2009 | EAHM vs. sedative-hypnotics | Guyuan Ningshen Tang | Decoction | Triticum aestivum L. [Poaceae; Tritici Fructus Levis], Zhen Zhu Mu [Pteridae; Margaritifera Usta Concha] 30g, Astragalus mongholicus Bunge [Fabaceae; Astragali Radix] 24g, Rehmannia glutinosa (Gaertner) Liboschitz ex Steudel [Scrophulariaceae; Rehmanniae Radix Recens], Alpinia oxyphylla Miq. [Zingiberaceae; Alpiniae Oxyphyllae Fructus], Epimedium sagittatum (Siebold & Zucc.) Maxim. [Berberidaceae; Epimedii Herba], Phellodendron amurense Rupr. [Rutaceae; Phellodendri Cortex], Zizyphus jujuba Miller var. spinosa Hu ex H. F. Chou [Rhamnaceae; Zizyphi Semen] 12g, Anemarrhena asphodeloides Bunge [Asparagaceae; Anemarrhenae Rhizoma], Acorus gramineus Aiton [Acoraceae; Acori Graminei Rhizoma] 9g, Cinnamomum cassia Presl [Lauraceae; Cinnamomi Cortex] 3g | Diazepam 2.5mg hs | Not reported | Not reported | Not reported | 28 days | None |
| Zhang 2012 | EAHM vs. sedative-hypnotics | Wuwei Shenping Tang | Decoction | Rehmannia glutinosa (Gaertn.) DC. [Orobanchaceae; Rehmanniae Radix Preparata], Eucommia ulmoides Oliver [Eucommiaceae; Eucommiae Cortex], Ophiopogon japonicus (Thunb.) Ker Gawl. [Asparagaceae; Liriopis seu Ophiopogonis Tuber] 15g, Cornus officinalis Siebold & Zucc. [Cornaceae; Corni Fructus] 12g, Cervus canadensis Erxleben [Cervidae; Cervi Cornu] 10g, Schisandra chinensis (Turcz.) Baill. [Schisandraceae; Schisandrae Fructus], Morinda officinalis How [Rubiaceae; Morindae Radix], Atractylodes lancea (Thunb.) DC. [Asteraceae; Atractylodis Rhizoma Alba], Paeonia lactiflora Pall. [Paeoniaceae; Paeoniae Radix] 9g, Aconitum carmichaelii Debeaux [Ranunculaceae; Aconiti Lateralis Radix Preparata], Polygala senega L. [Polygalaceae; Polygalae Radix], Pterocarpus santalinus Linné [Leguminosae; Santalini Lignum Rubrum], Glycyrrhiza glabra L. [Fabaceae; Glycyrrhizae Radix et Rhizoma] 6g, Zingiber officinale Roscoe [Zingiberaceae; Zingiberis Rhizoma Recens] 6 pieces | Estazolam 1mg hs | Not reported | Not reported | Not reported | 4 weeks | None |
| Zhang 2020 | EAHM + sedative-hypnotics vs. sedative-hypnotics | Songyu Yinxu Fang | Decoction | Rehmannia glutinosa (Gaertner) Liboschitz ex Steudel [Scrophulariaceae; Rehmanniae Radix Recens] 20g, Cornus officinalis Siebold & Zucc. [Cornaceae; Corni Fructus], Paeonia lactiflora Pall. [Paeoniaceae; Paeoniae Radix], Curcuma longa L. [Zingiberaceae; Curcumae Radix], Albizzia julibrissin Durazzini [Leguminosae; Albizziae Cortex], Alisma plantago-aquatica subsp. orientale (Sam.) Sam. [Alismataceae; Alismatis Rhizoma] 15g, Adenophora triphylla (Thunb.) A.DC. [Campanulaceae; Adenophorae Radix], Nardostachys chinensis Batal [Valerianaceae; Nardostachyos Radix et Rhizoma], Citrus × aurantium f. deliciosa (Ten.) M.Hiroe [Rutaceae; Citri Unshius Pericarpium Immaturus], Stellaria dichotoma Linné var. lanceolata Bge [Caryophyllaceae; Stellariae seu Gypsophilae Radix], Lycium barbarum L. [Solanaceae; Lycii Radicis Cortex] 10g, Glycyrrhiza glabra L. [Fabaceae; Glycyrrhizae Radix et Rhizoma] 6g | Estazolam 1mg hs | The Second Affiliated People's Hospital of Fujian University of Traditional Chinese Medicine | Not reported | Not reported | 4 weeks | None |
| Zhang 2021 | EAHM vs. sedative-hypnotics | Bushen Shugan Ningxin Tang | Decoction | Cornus officinalis Siebold & Zucc. [Cornaceae; Corni Fructus], Ligustrum lucidum W.T.Aiton [Oleaceae; Ligustri Fructus], Reynoutria multiflora (Thunb.) Moldenke [Polygonaceae; Polygoni Multiflori Caulis], Rehmannia glutinosa (Gaertn.) DC. [Orobanchaceae; Rehmanniae Radix Preparata], Angelica gigas Nakai [Apiaceae; Angelicae Gigantis Radix] 15g, Zizyphus jujuba Miller var. spinosa Hu ex H. F. Chou [Rhamnaceae; Zizyphi Semen], Albizzia julibrissin Durazzini [Leguminosae; Albizziae Cortex], Paeonia lactiflora Pall. [Paeoniaceae; Paeoniae Radix] 12g, Polygala senega L. [Polygalaceae; Polygalae Radix] 6g, Coptis chinensis Franch. [Ranunculaceae; Coptidis Rhizoma], Cinnamomum cassia Presl [Lauraceae; Cinnamomi Cortex] 3g | Estazolam 1mg hs | Not reported | Not reported | Not reported | 1 month | None |
| Zhao 2017 | EAHM vs. sedative-hypnotics | modified Suanzaoren Tang | Decoction | Zizyphus jujuba Miller var. spinosa Hu ex H. F. Chou [Rhamnaceae; Zizyphi Semen] 15g, Anemarrhena asphodeloides Bunge [Asparagaceae; Anemarrhenae Rhizoma], Poria cocos Wolf [Polyporaceae; Poria Sclerotium], Conioselinum anthriscoides 'Chuanxiong' [Apiaceae; Cnidii Rhizoma] 12g, Glycyrrhiza glabra L. [Fabaceae; Glycyrrhizae Radix et Rhizoma] 6g | Alprazolam 0.4mg hs | Not reported | Not reported | Not reported | 14 days | None |
| Zhao 2018 | EAHM vs. sedative-hypnotics | modified Suanzaoren Tang | Decoction | Zizyphus jujuba Miller var. spinosa Hu ex H. F. Chou [Rhamnaceae; Zizyphi Semen] 30g, Anemarrhena asphodeloides Bunge [Asparagaceae; Anemarrhenae Rhizoma], Poria cocos Wolf [Polyporaceae; Poria Sclertum Cum Pini Radix], Ophiopogon japonicus (Thunb.) Ker Gawl. [Asparagaceae; Liriopis seu Ophiopogonis Tuber] 15g, Conioselinum anthriscoides 'Chuanxiong' [Apiaceae; Cnidii Rhizoma] 10g, Glycyrrhiza glabra L. [Fabaceae; Glycyrrhizae Radix et Rhizoma] 6g, Schisandra chinensis (Turcz.) Baill. [Schisandraceae; Schisandrae Fructus] 5g | Estazolam 5-10mg hs | Not reported | Not reported | Not reported | 12 days | None |
| Zheng 2016 | EAHM + sedative-hypnotics vs. sedative-hypnotics | modified Guizhi Jia Longgu Muli Tang | Decoction | Fossilia Ossis Mastodi, Ostrea gigas Thunberg [Ostreidae; Ostreae Testa], Zizyphus jujuba Miller var. spinosa Hu ex H. F. Chou [Rhamnaceae; Zizyphi Semen], Reynoutria multiflora (Thunb.) Moldenke [Polygonaceae; Polygoni Multiflori Caulis] 20g, Neolitsea cassia (L.) Kosterm. [Lauraceae; Cinnamomi Ramulus], Paeonia lactiflora Pall. [Paeoniaceae; Paeoniae Radix], Glycyrrhiza glabra L. [Fabaceae; Glycyrrhizae Radix et Rhizoma], Bupleurum falcatum L. [Apiaceae; Bupleuri Radix], Scutellaria baicalensis Georgi [Lamiaceae; Scutellariae Radix], Citrus trifoliata L. [Rutaceae; Ponciri Fructus Immaturus], Poria cocos Wolf [Polyporaceae; Poria Sclerotium] 10g, Zingiber officinale Roscoe [Zingiberaceae; Zingiberis Rhizoma Recens] 3g, Ziziphus jujuba Mill. [Rhamnaceae; Zizyphi Fructus] 5 pieces | Alprazolam 0.4mg hs | Not reported | Not reported | Not reported | 4 weeks | None |
| Zheng 2020 | EAHM vs. sedative-hypnotics | Huanglian Ejiao Tang plus Ganmai Dazao Tang | Decoction | Triticum aestivum Linné [Gramineae; Tritici Fructus Levis] 30g, Scutellaria baicalensis Georgi [Lamiaceae; Scutellariae Radix], Paeonia lactiflora Pall. [Paeoniaceae; Paeoniae Radix], Poria cocos Wolf [Polyporaceae; Poria Sclerotium], Albizzia julibrissin Durazzini [Leguminosae; Albizziae Cortex] 15g, Equus asinus Linne [Equidae; Asini Corii Colla], Glycine max Merrill [Leguminosae; Glycine Semen Preparata], Gardenia jasminoides J.Ellis [Rubiaceae; Gardeniae Fructus], Glycyrrhiza glabra L. [Fabaceae; Glycyrrhizae Radix et Rhizoma] 10g, Coptis chinensis Franch. [Ranunculaceae; Coptidis Rhizoma] 6g, Gallus domesticus [Phasianidae; Galli Vitellus] 1 piece, Ziziphus jujuba Mill. [Rhamnaceae; Zizyphi Fructus] 5 pieces | Diazepam 2.5g bid | Not reported | Not reported | Not reported | 1 month | None |
| Zhu 2020 | EAHM vs. sedative-hypnotics | modified Buxin Xiaoyao Yin | Decoction | Paeonia lactiflora Pall. [Paeoniaceae; Paeoniae Radix], Fossilia Ossis Mastodi, Ostrea gigas Thunberg [Ostreidae; Ostreae Testa] 20g, Atractylodes lancea (Thunb.) DC. [Asteraceae; Atractylodis Rhizoma Alba] 19g, Haematitum 15g, Bupleurum falcatum L. [Apiaceae; Bupleuri Radix], Angelica gigas Nakai [Apiaceae; Angelicae Gigantis Radix] 12g, Poria cocos Wolf [Polyporaceae; Poria Sclerotium], Inula japonica Thunb. [Asteraceae; Inulae Flos], Glycine max Merrill [Leguminosae; Glycine Semen Preparata], Lophatherum gracile Bronghiart [Gramineae; Lophatheri Herba] 10g, Glycyrrhiza glabra L. [Fabaceae; Glycyrrhizae Radix et Rhizoma] 6g | Eszopiclone 3mg hs | Not reported | Not reported | Not reported | 4 weeks | None |

EAHM, East Asian herbal medicine; NR, not recorded.

**Supplement 5. Top 20 most frequently used botanical drugs**

| **Rank** | **Frequency** | **Name** |
| --- | --- | --- |
| **1** | **42** | *Zizyphus jujuba Miller var. spinosa Hu ex H. F. Chou* [Rhamnaceae; Zizyphi Semen] |
| **2** | **39** | *Glycyrrhiza glabra L.* [Fabaceae; Glycyrrhizae Radix et Rhizoma] |
| **3** | **32** | *Paeonia lactiflora Pall.* [Paeoniaceae; Paeoniae Radix] |
| **4** | **29** | *Poria cocos Wolf* [Polyporaceae; Poria Sclerotium] |
| **5** | **28** | *Bupleurum falcatum L.* [Apiaceae; Bupleuri Radix] |
| **6** | **24** | *Angelica gigas Nakai* [Apiaceae; Angelicae Gigantis Radix] |
| **7** | **24** | *Ostrea gigas Thunberg* [Ostreidae; Ostreae Testa] |
| **8** | **23** | *Fossilia Ossis Mastodi* |
| **9** | **22** | *Rehmannia glutinosa (Gaertner) Liboschitz ex Steudel* [Scrophulariaceae; Rehmanniae Radix Recens] |
| **10** | **19** | *Rehmannia glutinosa (Gaertn.) DC.* [Orobanchaceae; Rehmanniae Radix Preparata] |
| **11** | **19** | *Albizzia julibrissin Durazzini* [Leguminosae; Albizziae Cortex] |
| **12** | **18** | *Poria cocos Wolf* [Polyporaceae; Poria Sclertum Cum Pini Radix] |
| **13** | **18** | *Cornus officinalis Siebold & Zucc.* [Cornaceae; Corni Fructus] |
| **14** | **18** | *Reynoutria multiflora (Thunb.) Moldenke* [Polygonaceae; Polygoni Multiflori Caulis] |
| **15** | **18** | *Conioselinum anthriscoides 'Chuanxiong'* [Apiaceae; Cnidii Rhizoma] |
| **16** | **18** | *Coptis chinensis Franch.* [Ranunculaceae; Coptidis Rhizoma] |
| **17** | **15** | *Anemarrhena asphodeloides Bunge* [Asparagaceae; Anemarrhenae Rhizoma] |
| **18** | **14** | *Scutellaria baicalensis Georgi* [Lamiaceae; Scutellariae Radix] |
| **19** | **13** | *Lilium lancifolium Thunb.* [Liliaceae; Lilii Bulbus] |
| **20** | **12** | *Polygala senega L.* [Polygalaceae; Polygalae Radix] |

**Supplement 6.** **Risk of bias summary**

**- Risk of bias tool**

| 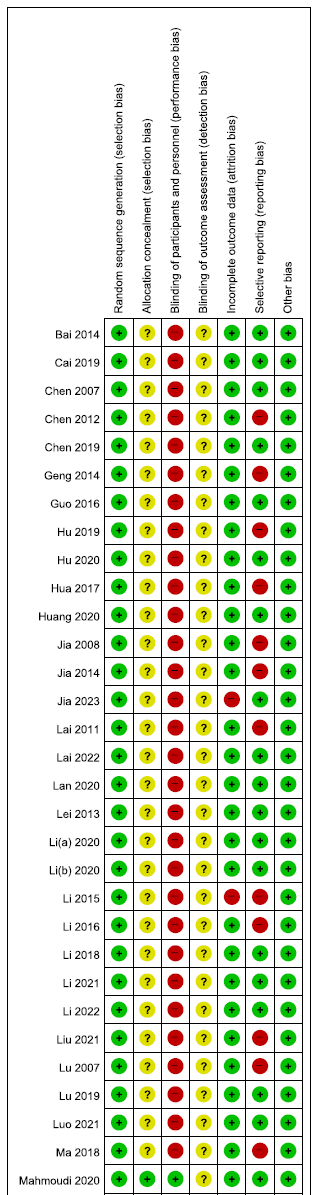 | 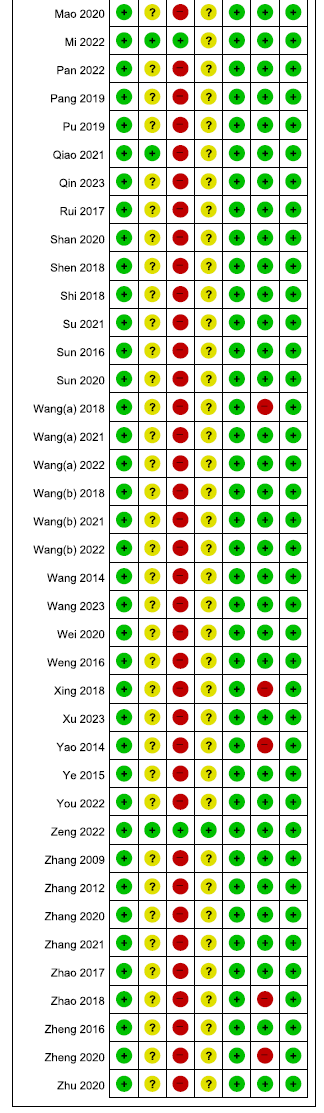 |
| --- | --- |

Low, unclear, and high risk, respectively, are represented with the following symbols: “+”, “?”, and “-”.

**- Revised risk of bias tool (RoB 2)**

| **Study ID** | **Bias arising from the randomization process** | **Bias due to deviations from intended interventions (effect of assignment to intervention)** | **Bias due to missing outcome data** | **Bias in measurement of the outcome** | **Bias in selection of the reported result** | **Overall risk of bias** |
| --- | --- | --- | --- | --- | --- | --- |
| Bai 2014 | Some concerns | Some concerns | Low risk of bias | Some concerns | Some concerns | Some concerns |
| Cai 2019 | Some concerns | Some concerns | Low risk of bias | Some concerns | Some concerns | Some concerns |
| Chen 2007 | Some concerns | Some concerns | Low risk of bias | Some concerns | Some concerns | Some concerns |
| Chen 2012 | Some concerns | Some concerns | Low risk of bias | Some concerns | Some concerns | Some concerns |
| Chen 2019 | Some concerns | Some concerns | Low risk of bias | Some concerns | Some concerns | Some concerns |
| Geng 2014 | Some concerns | Some concerns | Low risk of bias | Some concerns | Some concerns | Some concerns |
| Guo 2016 | Some concerns | Some concerns | Low risk of bias | Some concerns | Some concerns | Some concerns |
| Hu 2019 | Some concerns | Some concerns | Low risk of bias | Some concerns | Some concerns | Some concerns |
| Hu 2020 | Some concerns | Some concerns | Low risk of bias | Some concerns | Some concerns | Some concerns |
| Hua 2017 | Some concerns | Some concerns | Low risk of bias | Some concerns | Some concerns | Some concerns |
| Huang 2020 | Some concerns | Some concerns | Low risk of bias | Some concerns | Some concerns | Some concerns |
| Jia 2008 | Some concerns | Some concerns | Low risk of bias | Some concerns | Some concerns | Some concerns |
| Jia 2014 | Some concerns | Some concerns | Low risk of bias | Some concerns | Some concerns | Some concerns |
| Jia 2023 | Some concerns | Some concerns | Some concerns | Some concerns | Some concerns | Some concerns |
| Lai 2011 | Some concerns | Some concerns | Low risk of bias | Some concerns | Some concerns | Some concerns |
| Lai 2022 | Some concerns | Some concerns | Low risk of bias | Some concerns | Some concerns | Some concerns |
| Lan 2020 | Some concerns | Some concerns | Low risk of bias | Some concerns | Some concerns | Some concerns |
| Lei 2013 | Some concerns | Some concerns | Low risk of bias | Some concerns | Some concerns | Some concerns |
| Li 2015 | Some concerns | Some concerns | Some concerns | Some concerns | Some concerns | Some concerns |
| Li 2016 | Some concerns | Some concerns | Low risk of bias | Some concerns | Some concerns | Some concerns |
| Li 2018 | Some concerns | Some concerns | Low risk of bias | Some concerns | Some concerns | Some concerns |
| Li 2021 | Some concerns | Some concerns | Low risk of bias | Some concerns | Some concerns | Some concerns |
| Li 2022 | Some concerns | Some concerns | Low risk of bias | Some concerns | Some concerns | Some concerns |
| Li(a) 2020 | Some concerns | Some concerns | Low risk of bias | Some concerns | Some concerns | Some concerns |
| Li(b) 2020 | Some concerns | Some concerns | Low risk of bias | Some concerns | Some concerns | Some concerns |
| Liu 2021 | Some concerns | Some concerns | Low risk of bias | Some concerns | Some concerns | Some concerns |
| Lu 2007 | Some concerns | Some concerns | Low risk of bias | Some concerns | Some concerns | Some concerns |
| Lu 2019 | Some concerns | Some concerns | Low risk of bias | Some concerns | Some concerns | Some concerns |
| Luo 2021 | Some concerns | Some concerns | Low risk of bias | Some concerns | Some concerns | Some concerns |
| Ma 2018 | Some concerns | Some concerns | Low risk of bias | Some concerns | Some concerns | Some concerns |
| Mahmoudi 2020 | Low risk of bias | Low risk of bias | Low risk of bias | Some concerns | Some concerns | Some concerns |
| Mao 2020 | Some concerns | Some concerns | Low risk of bias | Some concerns | Some concerns | Some concerns |
| Mi 2022 | Low risk of bias | Some concerns | Low risk of bias | Some concerns | Some concerns | Some concerns |
| Pan 2022 | Some concerns | Some concerns | Low risk of bias | Some concerns | Some concerns | Some concerns |
| Pang 2019 | Some concerns | Some concerns | Low risk of bias | Some concerns | Some concerns | Some concerns |
| Pu 2019 | Some concerns | Some concerns | Low risk of bias | Some concerns | Some concerns | Some concerns |
| Qiao 2021 | Low risk of bias | Some concerns | Low risk of bias | Some concerns | Some concerns | Some concerns |
| Qin 2023 | Some concerns | Some concerns | Low risk of bias | Some concerns | Some concerns | Some concerns |
| Rui 2017 | Some concerns | Some concerns | Low risk of bias | Some concerns | Some concerns | Some concerns |
| Shan 2020 | Some concerns | Some concerns | Low risk of bias | Some concerns | Some concerns | Some concerns |
| Shen 2018 | Some concerns | Some concerns | Low risk of bias | Some concerns | Some concerns | Some concerns |
| Shi 2018 | Some concerns | Some concerns | Low risk of bias | Some concerns | Some concerns | Some concerns |
| Su 2021 | Some concerns | Some concerns | Low risk of bias | Some concerns | Some concerns | Some concerns |
| Sun 2016 | Some concerns | Some concerns | Low risk of bias | Some concerns | Some concerns | Some concerns |
| Sun 2020 | Some concerns | Some concerns | Low risk of bias | Some concerns | Some concerns | Some concerns |
| Wang 2014 | Some concerns | Some concerns | Low risk of bias | Some concerns | Some concerns | Some concerns |
| Wang 2023 | Some concerns | Some concerns | Low risk of bias | Some concerns | Some concerns | Some concerns |
| Wang(a) 2018 | Some concerns | Some concerns | Low risk of bias | Some concerns | Some concerns | Some concerns |
| Wang(a) 2021 | Some concerns | Some concerns | Low risk of bias | Some concerns | Some concerns | Some concerns |
| Wang(a) 2022 | Some concerns | Some concerns | Low risk of bias | Some concerns | Some concerns | Some concerns |
| Wang(b) 2018 | Some concerns | Some concerns | Low risk of bias | Some concerns | Some concerns | Some concerns |
| Wang(b) 2021 | Some concerns | Some concerns | Low risk of bias | Some concerns | Some concerns | Some concerns |
| Wang(b) 2022 | Some concerns | Some concerns | Low risk of bias | Some concerns | Some concerns | Some concerns |
| Wei 2020 | Some concerns | Some concerns | Low risk of bias | Some concerns | Some concerns | Some concerns |
| Weng 2016 | Some concerns | Some concerns | Low risk of bias | Some concerns | Some concerns | Some concerns |
| Xing 2018 | Some concerns | Some concerns | Low risk of bias | Some concerns | Some concerns | Some concerns |
| Xu 2023 | Some concerns | Some concerns | Low risk of bias | Some concerns | Some concerns | Some concerns |
| Yao 2014 | Some concerns | Some concerns | Low risk of bias | Some concerns | Some concerns | Some concerns |
| Ye 2015 | Some concerns | Some concerns | Low risk of bias | Some concerns | Some concerns | Some concerns |
| You 2022 | Some concerns | Some concerns | Low risk of bias | Some concerns | Some concerns | Some concerns |
| Zeng 2022 | Low risk of bias | Low risk of bias | Low risk of bias | Low risk of bias | Some concerns | Some concerns |
| Zhang 2009 | Some concerns | Some concerns | Low risk of bias | Some concerns | Some concerns | Some concerns |
| Zhang 2012 | Some concerns | Some concerns | Low risk of bias | Some concerns | Some concerns | Some concerns |
| Zhang 2020 | Some concerns | Some concerns | Low risk of bias | Some concerns | Some concerns | Some concerns |
| Zhang 2021 | Some concerns | Some concerns | Low risk of bias | Some concerns | Some concerns | Some concerns |
| Zhao 2017 | Some concerns | Some concerns | Low risk of bias | Some concerns | Some concerns | Some concerns |
| Zhao 2018 | Some concerns | Some concerns | Low risk of bias | Some concerns | Some concerns | Some concerns |
| Zheng 2016 | Some concerns | Some concerns | Low risk of bias | Some concerns | Some concerns | Some concerns |
| Zheng 2020 | Some concerns | Some concerns | Low risk of bias | Some concerns | Some concerns | Some concerns |
| Zhu 2020 | Some concerns | Some concerns | Low risk of bias | Some concerns | Some concerns | Some concerns |

**Supplement 7. Funnel plots**

1. EAHM compared with sedative-hypnotics: PSQI


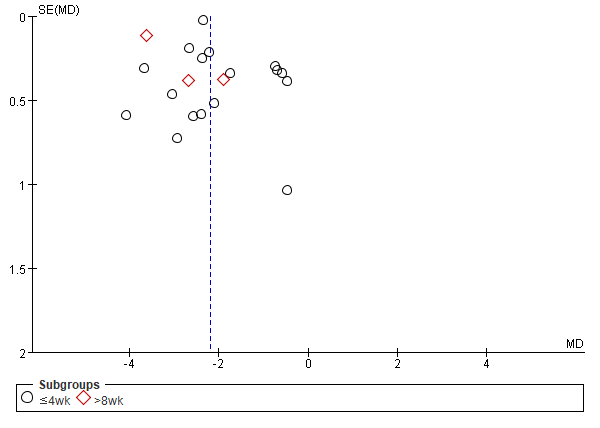


1. EAHM compared with sedative-hypnotics: TER based on insomnia symptom


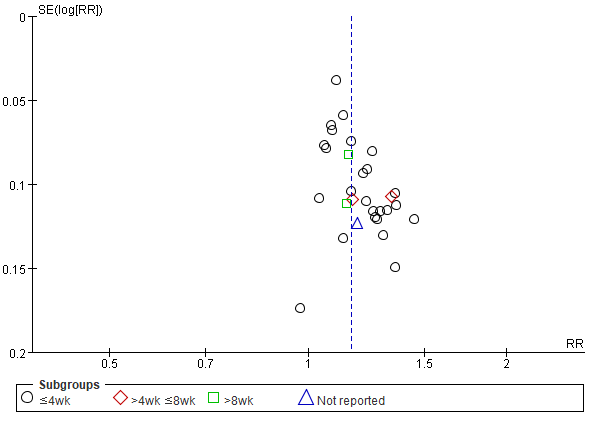


1. EAHM compared with sedative-hypnotics: Adverse events


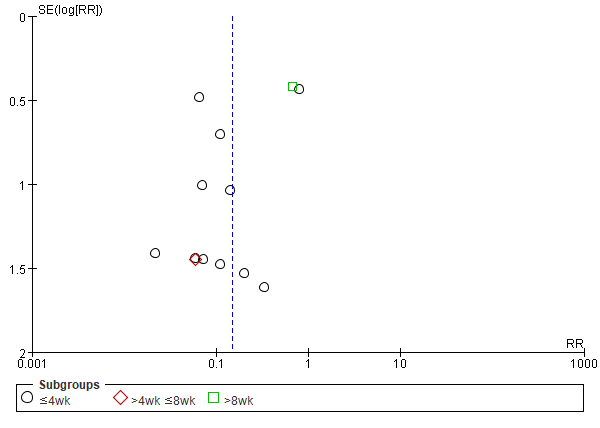


1. EAHM plus sedative-hypnotics compared with sedative-hypnotics alone: PSQI


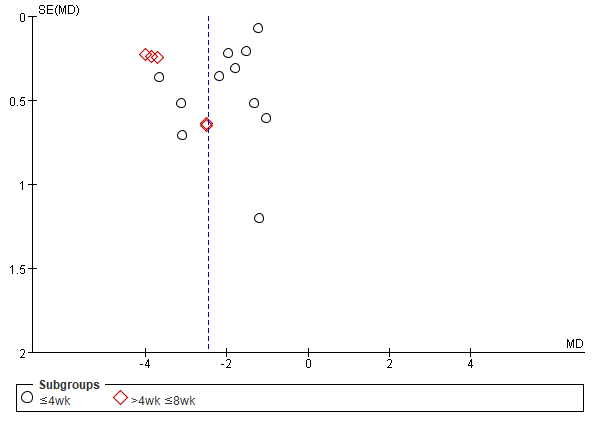


1. EAHM plus sedative-hypnotics compared with sedative-hypnotics alone: TER based on insomnia symptom


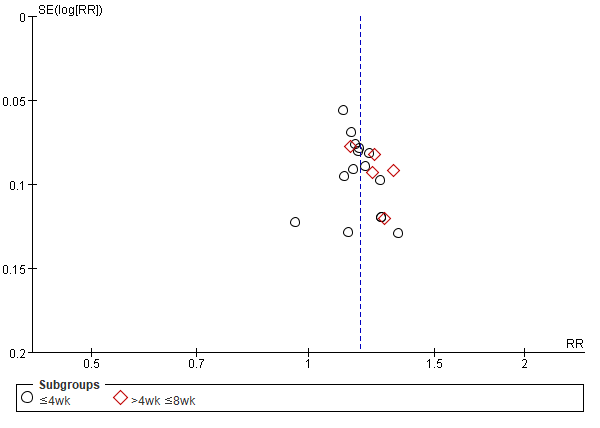


1. EAHM plus sedative-hypnotics compared with sedative-hypnotics alone: Adverse events


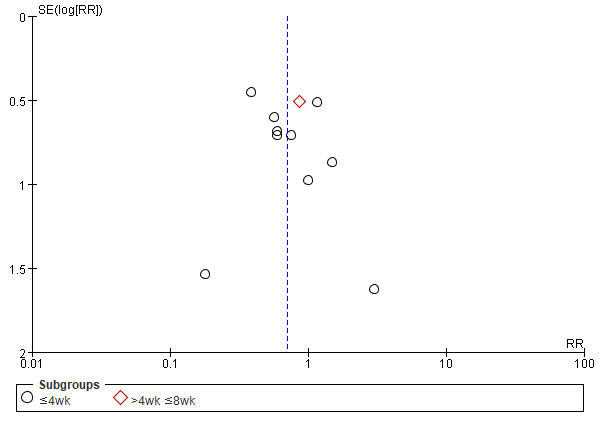

Supplement: Supplementary file 1 [file DataSheet1.docx]
